# Community-based interventions to support aging in place and functional independence in older adults: a systematic review of randomized controlled trials

**DOI:** 10.3389/fpubh.2026.1828271

**Published:** 2026-05-15

**Authors:** Adnan Kisa, Sezer Kisa

**Affiliations:** 1 School of Health Sciences, Kristiania University of Applied Sciences, Oslo, Norway; 2 Department of International Health and Sustainable Development, Tulane University, New Orleans, LA, United States; 3 Nursing and Health Promotion, Faculty of Health Sciences, Oslo Metropolitan University, Oslo, Norway

**Keywords:** aging in place, community-based interventions, healthy aging, independence, older adults, public health

## Abstract

**Background:**

As the global population ages, ensuring the autonomy and wellbeing of older adults living at home has become increasingly important. Supporting independence is central to aging in place, which requires access to appropriate health and social resources. In this context, community-based public health interventions may play an important role in addressing physical, cognitive, and psychosocial challenges associated with aging.

**Objective:**

This systematic review aimed to synthesize evidence from randomized controlled trials examining community-based public health interventions that promote independence among older adults and to identify key components associated with successful aging in place.

**Methods:**

A systematic review of randomized controlled trials was conducted, including 91 publications representing 85 independent randomized controlled trials, retrieved from MEDLINE/PubMed, Embase, Web of Science, CINAHL, and PsycINFO. Community-based public health interventions were defined as structured programs delivered in non-institutional settings to support health, functional ability, and autonomy among community-dwelling older adults. Data extraction included intervention characteristics, outcomes related to independence, and levels of community engagement. Outcomes were categorized as primary independence measures (e.g., ADL/IADL performance, disability status), functional capacity proxies (e.g., gait speed, balance), and psychosocial enablers of independence (e.g., self-efficacy, depressive symptoms) to support interpretive clarity. Methodological quality was assessed using the Joanna Briggs Institute checklist for randomized controlled trials. Findings were analyzed using thematic synthesis.

**Results:**

Across 91 publications representing 85 independent randomized controlled trials, 45% of publications were classified as low risk of bias, 31% as moderate, and 24% as high. Community-based interventions such as physical activity programs, cognitive and psychological support, multidomain models, nutrition interventions, social engagement initiatives, and rehabilitation strategies were generally associated with improvements in direct and indirect independence-related outcomes, including functional ability, mobility, cognitive performance, mental well-being, and social participation, although the magnitude and consistency of effects varied across intervention types and outcome domains. Interventions integrating multiple components and tailored delivery formats were frequently associated with improvements in functional outcomes and psychosocial enablers related to independence.

**Conclusion:**

Community-based public health interventions that address the multidimensional needs of older adults may support the maintenance of functional ability and independence in later life. Structured, scalable models that integrate physical, cognitive, and social components may strengthen aging-in-place strategies and inform future public health planning.

**Systematic review registration:**

https://www.crd.york.ac.uk/prospero/display_record.php?ID=CRD42024596045. The study protocol was registered with PROSPERO (CRD42024596045).

## Graphical abstract

**Figure:**
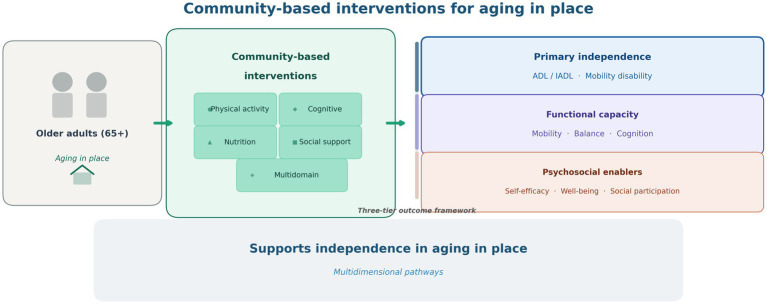

## 1 Introduction

The global population is undergoing a profound demographic shift, with the number of people aged 65 and older expected to double from 800 million in 2022 to nearly 1.6 billion by 2050 (1). In the United States (U.S.) alone, individuals over 65 are projected to make up 23% of the population by 2060, compared to 16% in 2020 (2). As life expectancy is projected to reach 19.6 years for 65-year-olds by 2050 (3) there is a need to develop strategies that promote independence for those who prefer aging in place. Yet, disparities in access to community support, rehabilitation services, and preventive care remain pronounced across regions and income levels. Older adults living in socioeconomically disadvantaged areas or belonging to minority groups often face barriers to health-promoting resources, digital access, and social participation. Addressing these inequities is central to achieving equitable health outcomes in aging populations and aligns with global public health goals under the SDG framework.

Aging in place refers to the ability to live in one’s home or community with access to health and social support (4, 5). Within this framework, independence may be understood as the capacity to maintain functional ability, autonomy in decision making, and self-management in daily life, including ADL and IADL (6, 7). Supporting independence, therefore, represents a central outcome and enabling condition of aging in place. This ability helps the well-being of older adults and reduces the strain on healthcare systems and social services by preventing unnecessary hospitalization and institutional care (8, 9). As the old-age dependency ratio (the proportion of people aged 65 and above to the working-age population) continues to rise globally (1), promoting independence among older adults becomes ever more vital for maintaining public health systems. In the U. S., Medicare spending is expected to increase from $829 billion in 2021 to $1.8 trillion by 2031 (10), driven largely by the aging population. These projections underscore the importance of cost-effective, community-based public health interventions that support independence while reducing long-term healthcare costs.

Community-based interventions that enhance physical, mental, and social well-being have been reported to improve functional status, mobility, and participation among older adults. However, such programs vary in design, delivery, and scope, and access to them differs across settings (11, 12). In low-resource or rural communities, limited infrastructure, underfunded primary care, and workforce shortages may restrict availability and participation. These programs emphasize prevention and aim to reduce the risk of chronic conditions and hospitalizations (13). For example, home modification programs, structured physical activity initiatives, and access to preventive healthcare have been shown to improve mobility, mental health, and social participation while reducing falls and related injuries (14, 15). In addition, interventions that promote social connectivity may reduce isolation, depressive symptoms, and cognitive decline among older adults (16–18). As community-based programs expand, identifying scalable and sustainable models remains critical for informing public health planning and implementation (19). For the purpose of this review, community-based public health interventions are defined as structured, non-institutional programs delivered in community settings such as homes, senior centers, or primary care linked community services, designed to promote health, prevent functional decline, or support autonomy among community-dwelling older adults. Thus, this systematic review of randomized controlled trials (RCTs) aims to synthesize evidence on community-based public health interventions that promote independence and support aging in place among older adults. The review addresses the following research questions:

- What types of community-based public health interventions have been evaluated to support independence among older adults?
- What independence-related outcomes are reported across these interventions?
- What factors influence the scalability and sustainability of these interventions in community settings?

While previous reviews have examined aging or health promotion programs more broadly, most have focused on single intervention domains or general healthy aging frameworks. Few have synthesized RCTs across diverse community-based interventions with independence as the central outcome among community-dwelling older adults. In addition, evidence regarding the long-term scalability and sustainability of such interventions remains dispersed across intervention areas. This systematic review addresses these gaps by rigorously synthesizing RCT evidence from diverse community-based public health interventions to identify common components, mechanisms, and implementation features associated with maintaining independence. The findings aim to inform public health planning and support the development of structured interventions that enable older adults to live independently and sustain functional ability within their communities.

## 2 Methods

### 2.1 Study design

This study was conducted as a systematic review of RCTs to synthesize evidence on community-based public health interventions promoting independence among older adults aged 65 years and over. The review was conducted in accordance with the Preferred Reporting Items for Systematic Reviews and Meta Analyses 2020 guidelines (20). The protocol was prospectively registered in PROSPERO (CRD42024596045).

### 2.2 Inclusion and exclusion criteria

Studies were eligible if they met the following criteria:

1. RCT design;
2. Participants aged 65 years or older living in community settings, or study populations primarily comprising older adults, defined as having a mean age of 65 years or above or clearly representing a later-life population;
3. Evaluation of community-based public health interventions delivered in non-institutional settings; and.
4. Reporting of outcomes related to independence.

Independence was operationalized *a priori* as maintenance or improvement in functional capacity, including activities of daily living (ADL), instrumental activities of daily living (IADL), mobility, cognitive capacity relevant to daily functioning, and psychosocial determinants influencing autonomous living, consistent with the World Health Organization’s framework of healthy ageing, which emphasizes functional ability as the core determinant of autonomy in later life (5, 15, 21, 22). Accordingly, outcomes reflecting independence included measures of physical function, ADL/IADL performance, mobility, frailty status, and related psychosocial indicators reported across trials.

Interventions included domains such as physical activity, cognitive support, nutrition, social support, rehabilitation, or multidomain approaches delivered in community settings. These interventions were considered public health relevant because they were delivered at the community level, emphasized prevention or preservation of functional ability, and were designed for implementation beyond specialized clinical settings. Many were embedded within scalable platforms such as senior centers, primary care linked services, community health worker programs, or structured home visit models. In the context of population ageing and aging-in-place policy frameworks, such interventions aim to maintain autonomy, delay functional decline, and reduce reliance on institutional care at the systems level.

Purely clinical rehabilitation programs delivered exclusively in hospital or institutional settings were excluded from this review. Eligible studies, published in English up to September 30, 2024, reported outcomes related to independence, including ADL, IADL, physical functioning, quality of life, mental well-being, or related functional outcomes. Studies were excluded if they were conducted in institutional or hospital settings, used non-randomized designs, or did not report independence-related outcomes.

### 2.3 Databases and search strategy

A comprehensive search strategy was developed to identify relevant literature across multiple electronic databases, including MEDLINE via PubMed, Embase, Web of Science, CINAHL, and PsycINFO. All databases were searched from inception to September 30, 2024, with the final search conducted on 1 October 2024 to ensure completeness. The search strategy combined controlled vocabulary and free-text terms using Boolean operators (AND, OR) and medical subject headings (MeSH) where applicable. Search terms were organized around three core concepts: population, intervention, and outcomes. To maximize sensitivity and minimize the risk of missing eligible studies, the search strategy was intentionally designed to be broad. Search terms were refined and expanded by examining terminology used in relevant RCTs and related systematic reviews identified during preliminary scoping searches.

In MEDLINE via PubMed, the built-in RCT publication type filter was applied during the database search to restrict results to RCTs. In databases where a validated RCT filter was not available, potential RCTs were identified using design descriptors such as “randomized,” “trial,” or “controlled trial,” and study design eligibility was subsequently confirmed during full-text review by verifying explicit random allocation to intervention and comparison groups. Title and abstract screening and full-text eligibility assessment were conducted independently by two reviewers. Disagreements at each stage were resolved through discussion and consensus.

The complete electronic search strategy for MEDLINE via PubMed, including all search terms and applied filters, is provided in Supplementary Table 6. Examples of the main search terms included:

- Population terms: (“elderly” OR “aged” OR “older” OR “geriatric” OR
- “elderly people” OR “old people” OR “seniors” OR “aging population”)
- Intervention terms: (“public health initiatives” OR “social care initiatives” OR “community-based approaches” OR “health programs” OR “aging in place” OR “home modifications” OR “social support networks” OR “health education” OR “preventive care programs”)
- Outcome terms: (“independence” OR “autonomy” OR “quality of life” OR “well-being” OR “health outcomes” OR “physical health” OR “mental health” OR “social connectivity” OR “mobility” OR “functional independence” OR “disability” OR “cognitive function” OR “loneliness” OR “social isolation”)

### 2.4 Data extraction strategy

To enhance conceptual clarity and ensure consistent outcome classification during data extraction, independent outcomes were categorized into three hierarchical levels. Primary independence outcomes included direct measures of functional autonomy such as ADL or IADL performance, disability status, institutionalization, or MMD, consistent with prior functional independence trials (5, 7, 15, 23). Functional capacity proxies included objective indicators of physical performance such as the Short Physical Performance Battery (SPPB) scores, gait speed, balance measures, falls, and strength assessments (22, 24–26). Enablers of independence included psychosocial and behavioral determinants such as self-efficacy, depressive symptoms, loneliness, social participation, and health literacy, which may influence an individual’s capacity to maintain autonomy in daily life (16, 27–30).

A structured data extraction form was developed and piloted prior to full extraction. The following key elements were extracted from each included publication: study characteristics (author(s), year, country, study design, and population characteristics); intervention characteristics (type of intervention, duration, delivery method, level of community involvement, and core components); outcomes (primary and secondary outcomes related to independence, quality of life, physical functioning, mental well-being, and reported scalability or sustainability considerations); and results (direction and magnitude of effects, outcome measures used, and reported implementation challenges). Two reviewers independently conducted data extraction using the predefined form. Discrepancies were resolved through discussion and consensus. Formal inter-rater reliability statistics were not calculated; however, the use of predefined extraction criteria, piloting of the data extraction form, and independent review by two reviewers were applied to enhance consistency and reduce subjective bias.

When multiple publications reported results from the same RCT, they were linked to a single underlying trial to avoid double counting. Some included articles represented secondary analyses of the same RCT (e.g., the Lifestyle Interventions and Independence for Elders [LIFE] study) and were retained as separate publications because they reported distinct outcomes. For synthesis and counting purposes, these publications were treated as one trial, and the review therefore includes 91 publications representing 85 independent RCTs (11, 22, 25).

### 2.5 Quality assessment

Methodological quality was assessed using the Joanna Briggs Institute (JBI) Critical Appraisal Checklist for RCTs (31). The checklist evaluates key domains of risk of bias, including random sequence generation, allocation concealment, baseline comparability, blinding of participants, personnel and outcome assessors, consistency and reliability of outcome measurement, completeness of follow-up, intention-to-treat analysis, and appropriateness of statistical analysis. Two reviewers independently assessed the risk of bias for each included publication using the predefined JBI criteria, and disagreements were resolved through discussion and consensus. Formal inter-rater reliability statistics were not calculated, but independent appraisal and consensus procedures were used to improve consistency in publication-level judgments. Detailed appraisal results for each included publication are presented in Supplementary Table 5.

Overall risk of bias was determined using a prespecified decision rule. Because blinding of participants and personnel is typically not feasible in behavioral and community-based interventions, domains D4 and D5 were retained in domain-level reporting but were not weighted as decisive in the overall study-level classification. This approach was based on methodological guidance for non-pharmacological and behavioral interventions, in which blinding of participants and personnel is often structurally difficult or not feasible, and these domains may therefore require cautious interpretation in overall study-level appraisal (32, 33). Studies were classified as low risk of bias if all remaining domains were rated as low risk, moderate risk of bias if at least one remaining domain was rated as unclear risk but none as high risk, and high risk of bias if one or more remaining domains were rated as high risk. Risk-of-bias assessments were conducted at the publication level because some RCTs were reported in multiple publications.

### 2.6 Data synthesis

Given the substantial heterogeneity in intervention types, outcome measures, reporting formats, and follow-up durations, a meta-analysis was not conducted. Outcome measures, intervention designs, and follow-up durations varied substantially across trials, with studies reporting diverse instruments, including SPPB, ADL/IADL scales, frailty indices, cognitive assessments, and quality-of-life measures. Because outcomes were measured using heterogeneous scales and reported at different follow-up intervals, quantitative pooling or meta-analysis was considered methodologically inappropriate. Across trials, more than 20 distinct outcome instruments were used, and follow-up durations ranged from several weeks to 4 years, which further limited statistical comparability and precluded meaningful pooled estimates. In addition, studies reported mixed directions and magnitudes of effect across intervention categories and outcome domains, further limiting the validity and interpretability of pooled summary estimates. Instead, findings were synthesized thematically using the approach described by Thomas and Harden (2008) (34), which involves line-by-line coding of extracted findings, the development of descriptive themes, and the generation of higher-order analytical themes through iterative comparison. Line-by-line coding was applied to the extracted text describing intervention characteristics, outcome patterns, and implementation-related observations reported in the included studies. Coding was primarily inductive in identifying recurrent findings across studies, however, was also informed deductively by the review objectives and predefined domains of independence, intervention type, scalability, and sustainability. Initial codes were grouped into descriptive themes corresponding to intervention domains and outcome patterns. These themes were then compared and refined iteratively across studies to generate higher-order analytical themes capturing common mechanisms, implementation features, and barriers relevant to independence. Two reviewers independently coded the extracted findings and discussed discrepancies until consensus was reached. Final themes were agreed upon through iterative review of the coding structure and alignment with the study aims. When reporting intervention effects, references to statistically significant or non-significant findings reflect the results reported by the original trial authors rather than pooled statistical analyses conducted in this review.

A wide range of instruments were used to assess independence-related outcomes, including the SPPB, ADL and IADL scales, frailty indices, cognitive assessments, and quality-of-life measures. Studies were therefore grouped deductively according to intervention type and outcome domains related to independence and functional ability. The analysis focused on identifying recurring intervention components and implementation features associated with reported improvements in independence-related outcomes, with particular attention to factors influencing feasibility, sustainability, and scalability across different community contexts.

For analytical clarity, interventions were categorized into seven groups: physical activity; cognitive and psychological interventions; multidomain approaches; health education; nutrition; social support and community engagement; and rehabilitation and supportive care. For synthesis purposes, related intervention types reported in Supplementary Table 2 were grouped into these seven broader analytical categories to facilitate thematic comparison across studies.

For the purposes of this review, scalability was defined as the potential for an intervention to be expanded, transferred, or implemented more broadly across community settings, populations, or service systems without substantial loss of feasibility or effectiveness, consistent with conceptualizations of scaling-up in public health implementation research (21, 35). Sustainability was defined as the capacity of an intervention to be maintained over time within routine community or health service delivery, including continued delivery, workforce support, participant engagement, and alignment with available organizational or financial resources (36). Few included studies explicitly evaluated scalability or sustainability as predefined implementation outcomes. Therefore, these dimensions were interpreted cautiously based on intervention delivery characteristics reported in the trials, including resource requirements, workforce involvement, integration with existing community or primary care systems, and delivery formats. Conceptual definitions of scalability and sustainability were informed by implementation science literature, including frameworks proposed by Milat et al. (35), Moore et al. (36), and the World Health Organization (21). Accordingly, interpretations related to scalability and sustainability should be understood as analytical inferences derived from intervention characteristics rather than as direct empirical outcomes reported by the trials. When available, information on scalability and sustainability was extracted from explicit descriptions provided by study authors regarding delivery models, workforce requirements, integration with existing services, follow-up structures, and reported implementation challenges.

## 3 Results

### 3.1 Characteristics of the studies

**Figure 1:**
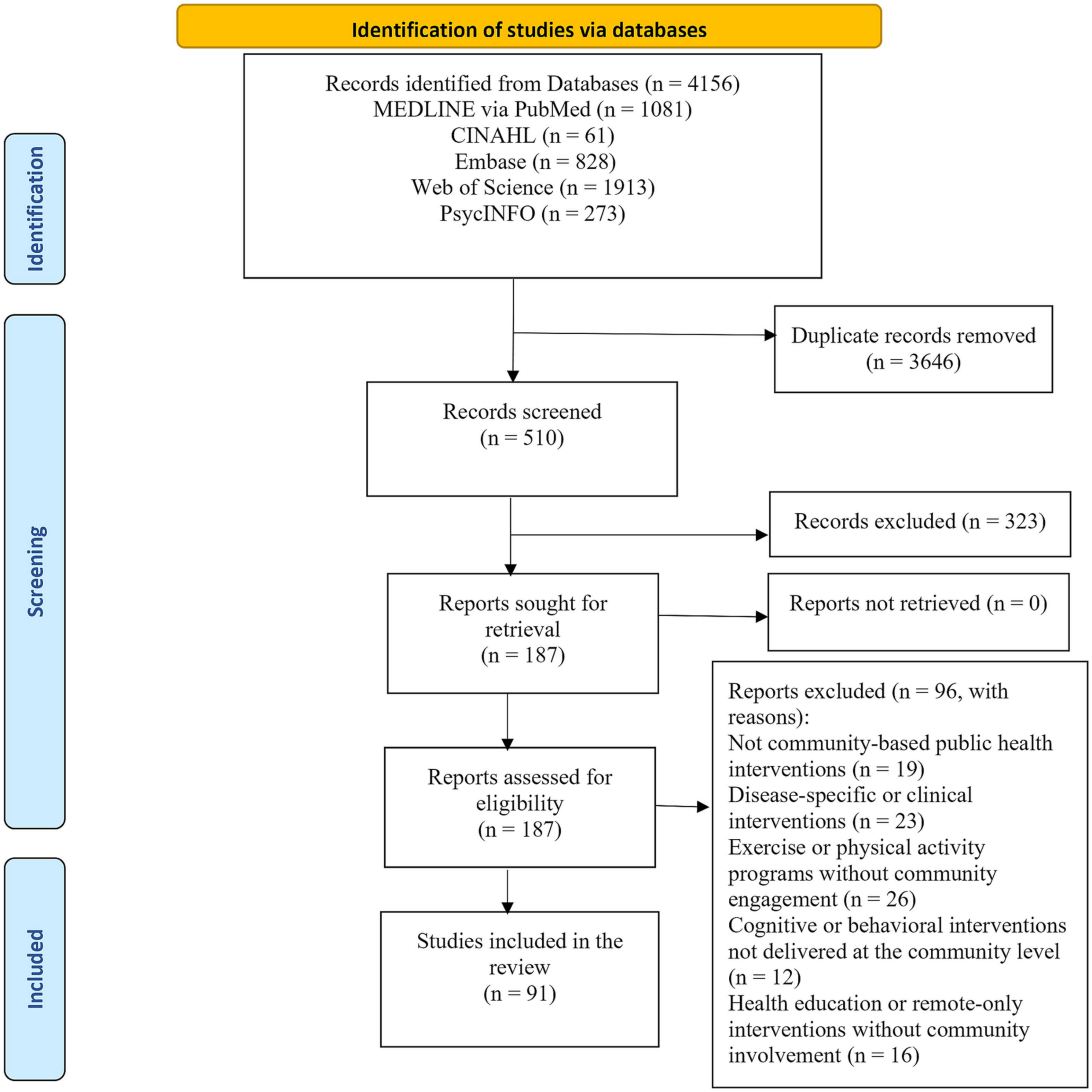
PRISMA 2020 flow diagram of the study selection process.

The study selection process is illustrated in the PRISMA flow diagram (Figure 1). The database search identified 4,156 records. After removing 3,646 duplicates, 510 records were screened based on titles and abstracts. A total of 187 full-text articles were assessed for eligibility, and 91 publications representing 85 independent RCTs were included in the final review (Table 1). The majority of studies were conducted in the United States (*n* = 38, 41.8%), followed by China (*n* = 9, 9.9%) and Japan (*n* = 6, 6.6%). Trials conducted in the United Kingdom, including those reported as England, accounted for 5.5% of studies (*n* = 5). Taiwan and Canada each contributed 4.4% (*n* = 4), while South Korea and Spain each represented 3.3% (*n* = 3). Sweden, Singapore, Hong Kong, and Germany each accounted for 2.2% of the included studies (*n* = 2). The remaining countries individually contributed 1.1% of studies (*n* = 1 each), including Australia, Switzerland, Portugal, Poland, the Philippines, New Zealand, the Netherlands, Malaysia, Israel, and Iran. One multisite trial conducted across China and Hong Kong (1.1%) was also included (Table 1). Because some publications evaluated multicomponent interventions, intervention categories were not mutually exclusive and are therefore presented as counts rather than percentages in Table 1. An increase in the number of published trials was observed in recent years. Detailed characteristics of the included RCTs are presented in Supplementary Table 1. Outcome measures and main reported results for each included study are presented in Supplementary Table 3.

**Table 1:** Summary characteristics of included RCTs.

**Characteristic**	**Details**
Total included studies	91 publications representing 85 independent RCTs
Total participants	47,625
Median sample size (range)	200 participants (25 to 9,803)
Median intervention duration (range)	6 months (1 to 48 months)
Geographic distribution	United States (*n* = 38, 41.8%); China (*n* = 9, 9.9%); Japan (*n* = 6, 6.6%); United Kingdom (including England) (*n* = 5, 5.5%); Canada (*n* = 4, 4.4%); Taiwan (*n* = 4, 4.4%); Spain (*n* = 3, 3.3%); South Korea (*n* = 3, 3.3%); Germany (*n* = 2, 2.2%); Hong Kong (*n* = 2, 2.2%); Singapore (*n* = 2, 2.2%); Sweden (*n* = 2, 2.2%); Australia (*n* = 1, 1.1%); Multisite study (China and Hong Kong) (*n* = 1, 1.1%); Iran (*n* = 1, 1.1%); Israel (*n* = 1, 1.1%); Malaysia (*n* = 1, 1.1%); Netherlands (*n* = 1, 1.1%); New Zealand (*n* = 1, 1.1%); Philippines (*n* = 1, 1.1%); Poland (*n* = 1, 1.1%); Portugal (*n* = 1, 1.1%); Switzerland (*n* = 1, 1.1%).
Intervention categories*	Physical activity or exercise (*n* = 29); Health education or self-management (*n* = 17); Multidomain or multicomponent (*n* = 13); Integrated or person-centered care (*n* = 10); Occupational therapy, home modification, or reablement (*n* = 9); Mind–body interventions including Tai Chi, mindfulness, or dance (*n* = 9); Cognitive training (*n* = 4); Nutritional interventions (*n* = 2)

*Intervention categories are not mutually exclusive; counts are based on the 91 included publications, and percentages are not presented because some publications were classified into more than one intervention category.

### 3.2 Risk of bias

Across studies, random sequence generation (D1) and baseline similarity between groups (D3) were most frequently rated as low risk of bias, indicating that most trials used appropriate randomization procedures and achieved comparable baseline characteristics. In contrast, blinding of participants (D4) and personnel delivering the intervention (D5) was commonly rated as high risk of bias, as expected in behavioral and community-based interventions, where blinding is often not feasible. Because blinding of participants and intervention providers is structurally impractical in non-pharmacological community interventions, domains D4 and D5 were retained in domain-level reporting but were not weighted as decisive in the overall study-level classification, consistent with established guidance for behavioral intervention reviews. Allocation concealment (D2) was frequently rated as unclear, reflecting incomplete reporting of allocation procedures in several trials. Some concerns were also observed in follow-up completeness (D10) in studies with longer intervention or follow-up periods. Based on the remaining domains, 45.1% of publications (41/91) were classified as low risk of bias, 30.8% (28/91) as moderate risk, and 24.2% (22/91) as high risk (Supplementary Table 5). Overall, these findings indicate variability in methodological rigor across the included studies.

### 3.3 Intervention characteristics and independence-related outcomes

**Figure 2:**
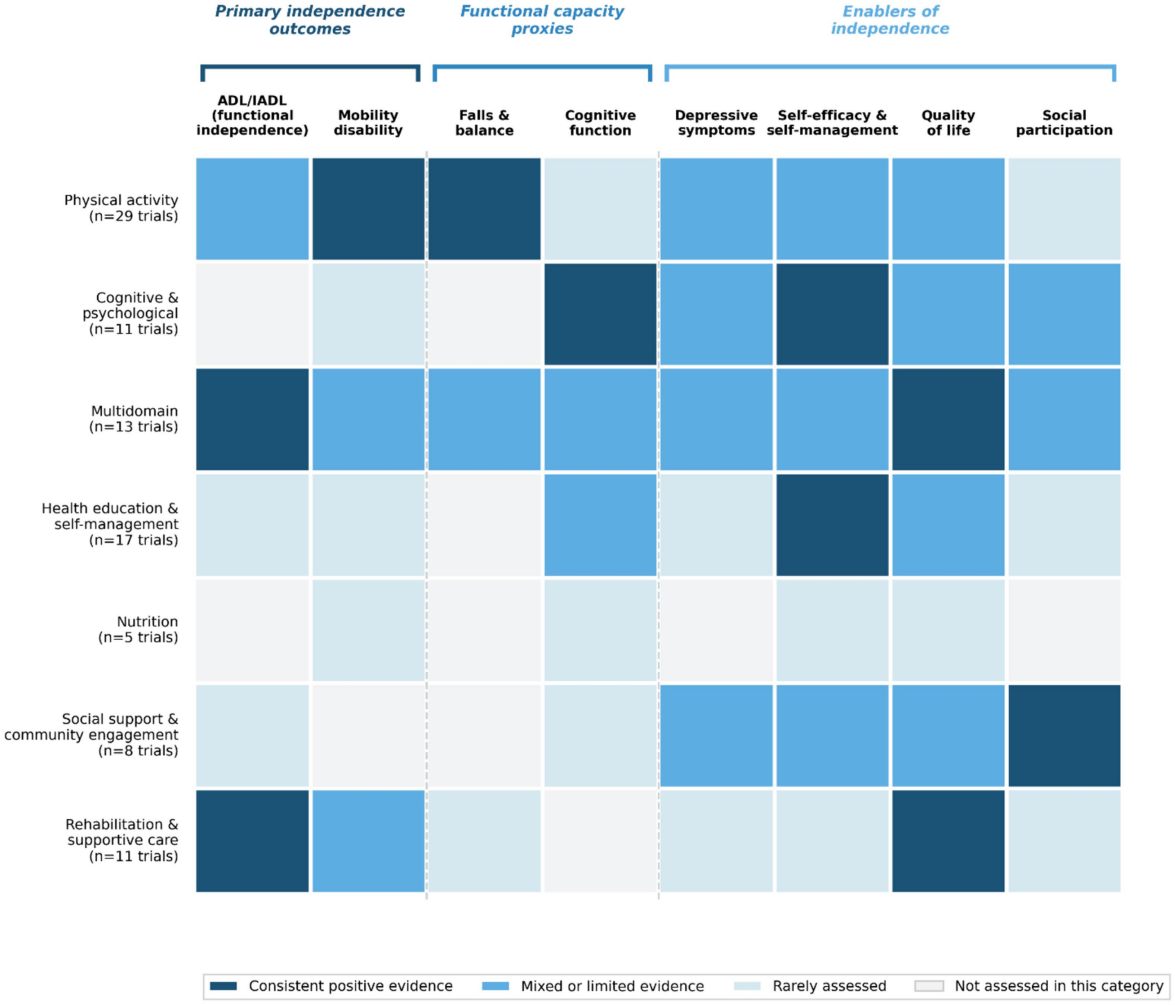
Mapping of community-based intervention categories to independence-related outcome domains across 85 RCTs. Outcome domains are organized according to the three-tier hierarchical framework: primary independence outcomes (ADL/IADL performance, mobility disability), functional capacity proxies (falls and balance, cognitive function), and enablers of independence (depressive symptoms, self-efficacy and self-management, quality of life, social participation). Evidence classification is based on the direction and consistency of reported findings across trials within each intervention category, as synthesized in the results (sections 3.3.1–3.3.7) and Supplementary Tables 2, 3. “Consistent positive evidence” indicates that most trials within that category reported improvements in the relevant outcome domain. “Mixed or limited evidence” indicates that some trials reported improvements while others reported null or inconsistent findings. “Rarely assessed” indicates that the outcome domain was evaluated in very few trials within that intervention category. “Not assessed” indicates that no included trials within the category evaluated that outcome domain.

Across 85 independent RCTs, seven categories of community-based interventions were identified: physical activity; cognitive and psychological interventions; multidomain interventions; health education and self-management; nutrition interventions; social support and community engagement; and rehabilitation and supportive care. For each category, intervention characteristics, outcome measures used, principal findings, and null or mixed results are reported below. Comparator conditions across studies included usual care, waiting lists, health education programs, fall prevention booklets, optometric assessments, general nutrition education, reminiscence therapy, sedentary activities, successful aging programs, and attention-control conditions such as relaxation CDs or structured non-exercise activities (4–9, 11, 12, 14–19, 22, 23, 25–27, 29, 30, 37–96) (Supplementary Table 2). The synthesis below places primary emphasis on direct measures of independence (e.g., ADL/IADL performance and disability status), while functional performance indicators and psychosocial outcomes are presented as indirect contributors within the hierarchical outcome framework described in the Methods. An overview of the distribution of intervention categories across independence-related outcome domains is presented in Figure 2.

#### 3.3.1 Physical activity interventions

Physical activity interventions included progressive resistance training, functional training, balance exercises, Tai Chi, structured walking programs, and combined aerobic and strength programs (8–18, 76–96). Outcome measures used across these trials included the SPPB, gait speed, quality-adjusted life years (QALYs), falls self-efficacy scales, fear of falling measures, and major mobility disability (MMD) assessments (6, 8, 11, 12, 22, 25, 37, 38, 40, 79, 80, 82, 83).

Across these trials, structured exercise programs were associated with improvements in strength, balance, and mobility through progressive resistance training, functional training, and flexibility components (6, 8, 24, 27, 37–43). Combined physical activity and health education programs reported improvements in physical function and reductions in MMD, including dose-dependent effects, while Tai Chi was associated with improvements in balance, strength, and flexibility among older adults with knee osteoarthritis (6, 25, 80). Mobility and fall-related outcomes were reported across long-term tailored programs and fall prevention interventions, including reductions in fall rates, improvements in gait, and gains in self-efficacy, particularly among participants with a history of falls (12, 26, 38, 40–43, 65, 82–88, 92, 94, 95). Regular physical activity was also linked to improvements in autonomy-related measures and reductions in frailty (41, 42, 89, 90), and among frail participants with mobility limitations, structured activity was associated with improvements in sleep quality and physical function (90, 94). Importantly, improvements in gait speed and balance were interpreted as functional capacity proxies supporting independence rather than direct measures of independence per se.

However, not all physical activity interventions demonstrated significant effects. The exercise arm of the ‘¡Caminemos!’ study did not produce significant improvements in depressive symptoms at all follow-up time points (27), and some fall prevention programs reported no significant reductions in fall rates in intention-to-treat analyses (26).

#### 3.3.2 Cognitive and psychological interventions

Cognitive and psychological interventions included cognitive training programs, dual-task training, mindfulness-based approaches, aerobic exercise targeting cognition, peer support, positive psychology, and self-management strategies (17, 28, 44–47, 49, 52, 53, 97, 98). Outcome measures included working memory assessments, executive function tests, psychomotor speed measures, sleep quality scales, depression inventories, self-efficacy instruments, and cognitive performance batteries (28, 46, 49, 53, 97).

Interventions focusing on cognition and psychological well-being reported improvements across several domains through cognitive training (44–46), social interaction (17, 47, 97), mindfulness-based approaches (98), and structured self-management strategies (28, 49, 52, 53). Specifically, improvements were reported in spatial working memory (97), global cognitive function (17), and reductions in depressive symptoms accompanied by gains in self-efficacy and social engagement (28, 47, 49). Cognitive stimulation approaches, including dual-task training, reported improvements in executive function, memory, and psychomotor speed (46, 53). Self-management programs were associated with improvements in cognitive performance and gait among at-risk older adults (44, 45). Psychosocial interventions also reported improvements in perceived cognitive change, satisfaction with social activities, and quality of life (52). Increases in resilience were observed in cognitive stimulation trials (21), while pain management and self-efficacy outcomes improved among older adults with chronic pain conditions (28). Programs targeting memory and executive functioning similarly reported gains relevant to everyday task performance and cognitive competence in daily activities (44).

Overall, cognitive and psychological interventions were frequently associated with improvements in executive function, mood, and perceived cognitive change, although the magnitude and persistence of these effects varied across trials. In many studies, these improvements appeared to support independence indirectly by strengthening executive functioning, cognitive control, and psychosocial factors such as self-efficacy and social engagement. Within the three-tier framework used in this review, these outcomes are interpreted primarily as functional capacity proxies and enablers of independence rather than direct indicators of functional autonomy.

However, some cognitive and psychological interventions did not demonstrate significant effects. The Aging Mastery Program, evaluated in a community-partnered trial, did not demonstrate statistically significant improvements in intention-to-treat analyses (16). Similarly, some self-management interventions showed limited effects on primary cognitive outcomes at longer follow-up (52).

#### 3.3.3 Multidomain interventions

Multidomain interventions combined two or more components such as physical activity, cognitive stimulation, social engagement, home modification, and nutritional support, and were delivered through tailored care plans, multidisciplinary team support, or technology-assisted platforms (5, 7, 14, 15, 18, 23, 48, 50, 51, 54–57, 99). Outcome measures included ADL and IADL dependence scales, frailty indices, life satisfaction measures, cognitive assessments, depressive symptom scales, and quality-of-life instruments (7, 14, 48, 55).

Multidomain interventions reported improvements in functional ability, independence, and health outcomes through tailored care plans (7, 14, 48, 50, 51), home modifications (15, 57, 99), and multidisciplinary team support (5, 18, 23, 54–56). Improvements were reported in life satisfaction, health perception, and quality of life (48), with mobility and fall prevention components associated with reductions in ADL and IADL limitations (14, 15). Gains in functional capacity and cognitive health were accompanied by reductions in dementia risk indicators and cognitive decline measures (18, 54, 55), while enhanced social engagement, reductions in frailty, and decreases in depressive symptoms were also observed (50, 51). Home modifications and environmental adaptations supported these gains by reducing ADL assistance needs, lowering disability risk, and enabling improvements in independence (5, 23, 51, 99). Improvements in health literacy further reinforced self-management behaviors and reductions in physical limitations across several trials (5, 18, 50, 56). Outcomes such as ADL and IADL performance constituted primary independence outcomes in these trials, while measures such as depressive symptoms and self-efficacy were interpreted as enablers of independence rather than direct functional autonomy indicators.

However, not all multidomain interventions demonstrated significant effects. The LoChro-Care intervention, a local collaborative and personalized care management program, reported no significant improvements in functional health or depressive symptoms over follow-up (50). The Embrace population-based, person-centered integrated care program showed no significant changes in well-being or ADL outcomes (56).

#### 3.3.4 Health education and self-management interventions

Health education and self-management interventions included programs targeting physical activity behavior, chronic disease management, hypertension control, dementia literacy, medication adherence, and elder abuse prevention (4, 29, 58–63, 66, 68, 100, 101). Outcome measures included blood pressure readings, health literacy scales, self-efficacy instruments, medication adherence rates, bone mineral density (BMD), and quality-of-life measures (4, 29, 58–63, 66, 68, 100, 101).

Health education interventions, including those focused on physical activity, reported improvements in health literacy, disease management, and self-care behaviors (58, 59, 63, 100), as well as in nutrition knowledge (60–62) and chronic disease management (4, 29, 66, 68, 101). Specifically, improvements were reported in hypertension management, blood pressure control, and lifestyle changes (61, 66), as well as in dementia literacy and cognitive health (29, 63). Programs also reported improvements in self-efficacy related to nutrition and elder abuse prevention (68, 100, 101). Targeted health education interventions were associated with improvements in quality of life and physical function in occupational therapy settings (4) and increased medication adherence (59, 60). Occupational therapy and physical education programs reported improvements in daily functioning and independence-related measures (4, 60, 66). Health education interventions targeting social engagement reported improvements in resilience and emotional well-being, while nutrition-focused programs reported improvements in BMD and quality of life (68). These outcomes were interpreted within the three-tier hierarchy: blood pressure control and medication adherence as enablers of independence, and improvements in daily functioning as primary independence outcomes.

Health education alone showed limited impact in preventing declines in ADL in several studies, whereas studies combining education with physical or cognitive training reported improvements across a broader range of functional outcomes (56, 58, 62).

#### 3.3.5 Nutrition interventions

Nutrition interventions included tailored nutritional counseling, dietary supplementation, and combined exercise and nutritional support programs targeting community-dwelling older adults (30, 51, 64, 65, 67). Outcome measures included dietary intake assessments, body composition measures, muscle strength tests, walking speed, aerobic capacity, cognitive function assessments, and healthcare cost analyses (30, 64, 65, 67).

Nutrition interventions reported improvements in dietary intake and health-related outcomes for chronic diseases through tailored nutritional counseling (30, 64, 67) and supplementation (51, 65). Enhanced nutritional intake and reductions in body fat were associated with improvements in physical mobility and health self-management measures (64, 65). Programs also reported increases in walking speed, aerobic capacity, and cognitive function (65, 67). Walking speed and aerobic capacity were interpreted as functional capacity proxies rather than direct independence measures, indicating meaningful gains relevant to functional autonomy. Nutritional interventions were more frequently associated with functional benefits when combined with structured exercise programs, particularly in addressing sarcopenic obesity (65).

#### 3.3.6 Social support and community engagement interventions

Social support and community engagement interventions included peer-led programs, community partnership models, group-based social activities, health-social partnership programs, and integrated community care approaches (9, 16, 72, 74, 102). Outcome measures included loneliness scales, social participation measures, functional independence assessments, quality-of-life instruments, mental health inventories, and injury-related cost analyses (9, 16, 72, 74, 102).

Social support and community engagement interventions were associated with enhanced social interaction, reduced isolation, greater community participation, improved functional independence, and improved quality of life (9, 16, 72–75, 102). Similar social participation gains were also reported in multidomain trials that incorporated explicit social engagement components (99). Reductions in loneliness and improvements in social participation were classified as enablers of independence, reflecting their role in sustaining psychosocial resources that support autonomous daily living. However, not all social support interventions demonstrated significant effects. The Aging Mastery Program, which combined social engagement with health education and goal-setting components, was also discussed in the cognitive and psychological intervention category because of its multidimensional design. In intention-to-treat analyses, the program did not demonstrate statistically significant improvements (16).

#### 3.3.7 Rehabilitation and supportive care interventions

Rehabilitation and supportive care interventions included individualized care plans, occupational therapy-led programs, home safety modifications, assistive device provision, caregiver-involved programs, palliative and supportive care, and self-management support for chronic conditions (13, 69–71, 76–78, 91, 93, 96, 103, 104). Outcome measures included functional independence scales, ADL performance assessments, hospital readmission rates, home safety evaluations, healthcare utilization data, caregiver burden measures, and quality-of-life instruments (13, 69–71, 76–78, 91, 93, 96, 103, 104).

Rehabilitation and supportive care interventions reported improvements in recovery outcomes, functional independence, and quality of life through individualized care plans (69–71, 78, 104), assistive devices (76, 77, 103), and home safety modifications (13, 91, 93, 96). Specifically, reductions in pain-related disability and improvements in mobility and quality of life were reported (91, 93). These interventions were also associated with reductions in hospital readmissions and long-term care utilization (77, 104). Programs addressing caregiver involvement reported improvements in quality of life and self-reported health (69, 76, 103), as well as reductions in caregiver burden and improvements in life satisfaction (13, 70, 71, 78). ADL performance and hospital readmission outcomes served as primary indicators of independence in these trials, while caregiver burden and quality of life were considered enablers of sustained independent living.

### 3.4 Mechanisms, scalability, sustainability, and implementation challenges

#### 3.4.1 Mechanisms through which community-based interventions support independence

Across intervention categories, the included studies indicate recurring mechanisms through which community-based interventions relevant to public health practice support independence among older adults. At the physical level, improvements in strength, balance, gait speed, and endurance were consistently linked with reduced mobility disability, lower falls risk, and better performance in daily activities across structured physical activity, balance, and multimodal programs (6, 11, 22, 25, 26, 37, 79, 95). These physical performance improvements represent functional capacity proxies through which greater primary independence is achieved and sustained. Cognitive and executive capacity was also strengthened through community-delivered cognitive training, dual-task approaches, and aerobic activity programs targeting cognition, with reported gains in executive function, memory, and cognitive performance relevant to functional competence (18, 44–46, 53, 54, 97, 105, 106). Psychosocial resources, including self-efficacy, reduced depressive symptoms, and social connectedness, which were classified as enablers of independence, were improved in interventions incorporating peer support, positive psychology, purpose-driven engagement, group-based activities, and culturally tailored programs. These approaches supported sustained participation and adaptive coping among older adults (16, 27, 28, 47, 49, 52, 72, 98). At the environmental level, home-based adaptations and occupational therapy-led modifications reduced functional barriers and supported aging in place through hazard reduction and assistive strategies, with reported reductions in functional difficulties and disability risk (9, 14, 15, 57, 77). Integration with community and primary care infrastructures, including person-centered care, health-social partnerships, and community health service delivery, was associated with improved continuity, adherence support, and maintenance of independence-related outcomes over follow-up (50, 56, 73–75, 78, 104).

#### 3.4.2 Scalability and sustainability

The observations below should be interpreted cautiously, as scalability and sustainability were not consistently evaluated as explicit trial outcomes and were often inferred from intervention characteristics, delivery models, and follow-up descriptions.

Physical activity interventions appeared more scalable when they used cost-conscious and adaptable delivery approaches. These included training visual rehabilitation officers (76), implementing minimal-equipment exercise programs (37), and utilizing standardized monitoring tools such as the SPPB (79). Sustainability of these interventions appeared more plausible when supported by stable funding mechanisms, trained personnel, and ongoing community engagement. Community-based programs, such as structured walking groups, reported maintenance of physical benefits over longer follow-up periods (38), while home-based exercise models were described as adaptable across diverse community settings (24) (Supplementary Table 4).

Cognitive and psychological interventions appeared more scalable when they leveraged existing community resources (97), incorporated non-professional staff (28), and adopted peer-led delivery models. These approaches may be particularly relevant for rural or resource-constrained areas, where low-cost and culturally relevant support structures are important (47, 49). Integration with primary healthcare or community health services also appeared to support scalability potential; however, longer-term sustainability seemed to depend on participant adherence, ongoing facilitator training, and continued community engagement (17, 98).

Healthcare-integrated multidomain interventions, including standardized care plans and coordinated case management models (50), appeared more scalable when supported by interdisciplinary collaboration and sustained community involvement (51). Telehealth platforms and digital delivery models (99), as well as low-cost technology-based education tools such as WeChat-based interventions (55), were described as potentially scalable alternatives, particularly when supported by both healthcare professionals and community stakeholders. Interdisciplinary coordination and cultural adaptation were recurrent features that appeared to improve feasibility across diverse contexts (18, 54).

Health education interventions appeared more scalable when delivered within established community infrastructures (66), using affordable educational materials (60), and standardized program formats (100). Sustainability appeared more likely when supported by facilitator training (61), interdisciplinary collaboration (63), and active community participation (101). The involvement of non-professional or community-based facilitators (4) and culturally tailored resources (62) was described as a potential strategy to reduce implementation costs and improve engagement.

Nutrition interventions appeared more scalable when embedded within existing healthcare or community service structures and delivered through relatively low-cost counseling models (64). Long-term sustainability appeared to depend on continued dietitian involvement (30), adherence monitoring, and structured follow-up systems (65). In rural settings, affordable and flexible delivery approaches appeared particularly important, with digital tools and community-based follow-up potentially supporting continued participation (67).

Rehabilitation and supportive care interventions appeared more scalable when integrated into community health service frameworks (70). Sustainability appeared more likely when supported by trained multidisciplinary teams (103), high participant satisfaction (71), and integration with existing care pathways. Programs incorporating low-cost assistive tools (e.g., Thera-Bands) and home-based adaptive modifications were described as feasible approaches to extend reach (58, 78, 91, 93, 96). Ongoing facilitator involvement and alignment with community health infrastructure were also described as potential contributors to longer-term sustainability.

#### 3.4.3 Implementation challenges

Community-based interventions relevant to public health practice reported several implementation challenges across categories. Participant engagement and adherence difficulties were among the most frequently reported barriers (4, 11, 12, 19, 27, 37, 41–43, 45, 46, 48, 53, 64, 69, 79, 80, 84–86, 90, 92, 97, 101, 103, 105). Resource and funding constraints limited the feasibility and sustainability of several programs (9, 24, 49, 54, 58, 60, 66, 100). Technological and infrastructure barriers were identified in interventions utilizing digital platforms, telehealth, or remote delivery formats (17, 30, 70, 75, 81, 83, 99). Cultural and community-specific challenges, including language barriers, low health literacy, and limited cultural tailoring, were reported across diverse implementation settings (8, 16, 18, 28, 40, 50, 51, 55, 56, 61, 62, 71, 74, 87, 88, 102). Challenges related to scalability and long-term sustainability were also reported across intervention categories (7, 13, 15, 23, 26, 29, 30, 44, 52, 57, 67, 68, 72, 77, 78, 89, 91, 93–96, 104).

## 4 Discussion

This systematic review synthesizes evidence suggesting that community-based interventions relevant to public health practice may support older adults’ independence and well-being across physical, cognitive, and psychosocial domains. Across countries and intervention types, the evidence generally indicates that physical activity interventions, including tai chi, structured aerobic exercise, resistance training, and multidomain activity models, play an important role in improving mobility, balance, gait, and overall physical functioning (8, 22, 25, 80, 95). These outcomes are not merely intermediate fitness indicators; they are closely linked to fall risk, mobility disability, and the capacity to perform ADL without assistance. The LIFE trials provide particularly robust evidence that structured physical activity can reduce MMD and improve physical performance among older adults at elevated risk, thereby supporting the view that physical activity may represent a central strategy for preserving autonomy in later life rather than a supplementary lifestyle option (6, 22, 90). Importantly, the translation of structured interventions into community settings with maintained functional benefits suggests that effectiveness may not be confined to highly controlled research environments and, in some contexts, may be sustained under real-world conditions (19).

Beyond physical capacity, cognitive and psychological interventions were associated with improvements in memory, executive function, global cognition, emotional well-being, and self-efficacy (18, 44, 45, 54, 55, 97, 105, 106). Mind, body, and resilience-oriented programs demonstrated reductions in depressive symptoms and improvements in psychological outcomes among community-dwelling older adults (27, 47, 59, 98). These findings reinforce a multidimensional conceptualization of independence. Autonomy in later life depends not only on preserved mobility but also on cognitive vitality, emotional regulation, and the perceived capacity to manage daily demands. Consistent with the three-tier hierarchical framework applied in this review, cognitive gains may primarily function as proxies for functional capacity and enablers of independence rather than as direct measures of functional autonomy. Interventions that explicitly combine physical and cognitive elements appear particularly aligned with the intertwined trajectories of motor and cognitive aging and may better support functional competence in complex, everyday contexts than single-domain approaches (18, 54, 55). Improvements in psychosocial outcomes and functional proxies should therefore be interpreted as indirect contributors rather than as direct evidence of functional independence.

Social support, rehabilitation, and integrated care models further contributed to independence by addressing functional recovery, symptom burden, and environmental constraints. Community-partnered and peer-delivered programs improved engagement and psychosocial outcomes, including self-efficacy and perceived functional change (16, 28, 49, 52). Multidisciplinary and home-based approaches, including supportive care models and comprehensive assessment linked to follow-up, demonstrated improvements in function and health-related quality of life among older adults with chronic conditions and frailty (5, 23, 70, 73, 74, 102). These findings suggest that independence may be supported through coordinated support structures that extend beyond individual capacity building. Community-based recruitment, delivery in familiar settings, tailored progression, and ongoing monitoring appear to function as enabling conditions that strengthen adherence and sustained participation, thereby supporting more durable functional gains. While physical activity interventions demonstrated the most consistent improvements in mobility-related outcomes, psychosocial and supportive care interventions appeared particularly important for sustaining engagement, emotional well-being, and longer-term participation in community-based programs.

From a comparative perspective, the intervention categories differed in both outcome profiles and implementation demands. Physical activity interventions most consistently reported improvements in mobility-related outcomes such as balance, gait speed, and MMD (6, 8, 25, 90). Multidomain interventions appeared to influence broader independence-related outcomes, including ADL/IADL performance, frailty, and overall functional ability (14, 18, 51, 54, 55). Cognitive and psychological interventions primarily contributed through improvements in executive function, mood, self-efficacy, and perceived cognitive change, suggesting a stronger role as enablers of independence rather than direct determinants of functional autonomy (28, 44–47, 49, 97, 98, 106). Social support and community engagement interventions were particularly relevant for maintaining participation, reducing loneliness, and supporting psychosocial conditions that sustain independent living (9, 16, 72–75). In terms of delivery, low-cost group-based programs, home-based models, and interventions embedded within existing community infrastructures appeared more scalable, whereas interdisciplinary or person-centered models offered broader support but required greater coordination, workforce capacity, and sustained implementation fidelity (4, 24, 50, 56, 100, 101).

These findings are broadly consistent with previous studies and systematic reviews indicating that multidimensional community-based interventions may play an important role in supporting functional ability and independence in later life (107, 108). Earlier systematic reviews have similarly reported that physical activity interventions are among the most effective strategies for improving mobility and preventing functional decline among community-dwelling older adults (109, 110). At the same time, evidence from multidomain intervention trials and related studies suggests that interventions addressing multiple domains, including physical activity, cognitive engagement, and social participation, may produce broader benefits for maintaining independence and quality of life (14, 18, 51, 54, 55, 111). Building on this body of evidence, the present review synthesizes findings across a broader range of community-based public health interventions and highlights how intervention delivery formats, community integration, and contextual conditions may influence implementation feasibility and longer-term sustainability. Recent evidence further supports these observations. A 2024 systematic review and network meta-analysis by Crocker et al. reported that community-based complex interventions may have small positive effects on sustaining independence, while also noting that evidence was insufficient to determine the most effective service models across intervention types overall; individualized care planning, including medicines optimization and regular follow-up reviews, was identified as the intervention most likely to sustain independence (112). A 2025 systematic review by Elhag et al. similarly found that community-based interventions may benefit physical health, psychological well-being, and social connections among older adults, consistent with the multidimensional pattern observed in the present review (113).

The cross-country distribution of evidence adds important contextual nuance. In high-income countries with developed primary care and municipal infrastructures, community interventions are frequently embedded within formal systems or linked to established research-to-practice pipelines. In the United States, the LIFE program and related analyses illustrate how well-resourced, protocolized physical activity interventions can be implemented over extended follow-up periods and evaluated against clinically meaningful mobility outcomes (6, 8, 22, 28). Hybrid models combining supervised center-based sessions with home-based activity targets were commonly employed, reflecting adaptation to functional limitations while maintaining structured progression (6, 8, 22). In European contexts, person-centered integrated care models and population-based service approaches explicitly emphasized self-management, self-determination, and coordinated multidisciplinary follow-up, aligning with broader aging-in-place frameworks and municipal health strategies (56, 69). Pragmatic falls prevention programs delivered through health service networks in the United Kingdom further suggest that even modest functional gains can be population-relevant when interventions are feasible for wide-scale deployment (13). In these settings, the central implementation challenge appears less related to efficacy and more to integration, commissioning, workforce training, and sustained financing.

In middle-income and rapidly aging settings, community delivery often functions as an access strategy designed to address affordability, service continuity, and workforce limitations. In China, community health management and integrated hypertension programs combined lifestyle counseling, monitoring, and follow-up within community health service structures, reflecting an emphasis on reach and continuity in everyday environments (75, 100). In Iran, empowerment-based education addressing safety and self-efficacy targeted psychosocial determinants that directly influence vulnerability and autonomy (101). Programs implemented in low-income housing settings in Malaysia demonstrate that group-based, resource-efficient formats can improve functional performance and quality of life among underserved populations (12). These models suggest that scalability in such contexts may depend more on strengthening community-level capacity, including training for community health workers and local service providers, than on specialist-intensive designs. Together, these patterns suggest that while high-income countries often prioritize integration within established health systems, interventions in middle-income settings more frequently emphasize accessibility, community delivery, and workforce adaptability.

Across both high and middle-income contexts, cultural adaptation and community embeddedness appear to function as substantive mechanisms rather than superficial modifications. Culturally tailored dance and attribution retraining programs among older Latino populations in the United States (84, 85, 89), culturally grounded tai chi and combined music and tai chi interventions in East Asian settings (47, 63, 80), and community health worker-led positive psychology interventions for underserved African American older adults (28) demonstrate that social relevance and contextual fit likely influence engagement, adherence, and ultimately functional outcomes. Because many interventions rely on sustained participation to produce mobility and cognitive benefits, cultural resonance and accessibility may be central to effectiveness.

From a systems perspective, the convergence of findings across intervention categories supports the potential integration of community-based strategies within existing health and social care infrastructures. Physical activity programs with structured progression can be delivered through municipal services, primary care partnerships, and community organizations (6, 8, 22). Cognitive and psychosocial programs can be embedded within senior centers or community networks, supported by trained facilitators and peer models (18, 28, 44, 49, 52). Health education and self-management initiatives appear most effective when interactive and contextually tailored, strengthening perceived control and sustained behavioral change that underpin independence (29, 60, 62, 66). Coordinated multidomain approaches that combine physical activity, cognitive stimulation, and social participation may provide greater resilience against functional decline than isolated single-component interventions, particularly among older adults with multimorbidity or early functional impairment (18, 54, 55, 102).

Despite the generally positive pattern of findings, several trials reported null or mixed outcomes. Null findings observed in trials such as LoChro-Care, Embrace, and the Aging Mastery Program highlight the complexity of implementing coordinated community-based interventions. These programs often involve multiple service components and rely heavily on sustained participant engagement and system-level coordination. Variability in implementation fidelity, intervention intensity, and participant adherence may partially explain why some theoretically strong interventions failed to produce measurable functional gains. Metzner et al. (2023) reported no significant improvement in functional health or depressive symptoms following the LoChro-Care intervention (50), and Spoorenberg et al. (2018) found no meaningful changes in well-being or ADL outcomes in the Embrace integrated care program (56). Similarly, Guerrero et al. (2020) observed no statistically significant effects in intention-to-treat analyses of the Aging Mastery Program (16). In addition, approximately one-quarter of the included studies were classified as high risk of bias, further limiting confidence in some reported effects. These findings suggest that although community-based interventions show considerable promise for supporting independence among older adults, their effectiveness may vary across intervention models, populations, and implementation contexts. Successful implementation, therefore, depends not only on intervention design but also on contextual fit, implementation fidelity, and sustained community engagement.

Scalability may depend on more than low-cost design. Although group-based formats that use existing community spaces and minimal equipment are promising for broader implementation (58, 63, 95), sustained benefits may require alignment with funding structures, workforce development, and integration with primary care and municipal planning (50, 56, 74). Interventions that cultivate local ownership and cross-sector partnerships appear better positioned to achieve continuity beyond initial implementation phases.

From a public health perspective, equity considerations are critical when designing community-based interventions for older adults. Structural barriers, such as transportation limitations, language differences, financial constraints, and unequal access to community services, may limit participation among vulnerable populations. Future interventions should therefore prioritize inclusive delivery models, culturally adapted programs, and partnerships with local community organizations to ensure that aging-in-place strategies reach socially disadvantaged groups. Older adults in rural areas, low-income communities, and culturally diverse populations face structural barriers to transportation, cost, language, and limited-service availability. Evidence from culturally tailored interventions, peer support models, and housing site delivery indicates that locally embedded approaches can enhance participation and outcomes among underserved groups (12, 28, 84, 85). Ensuring accessibility for individuals with multimorbidity, sensory impairment, or cognitive decline is critical to avoid reinforcing disparities in later life. From a policy perspective, integrating community-based interventions into primary care systems, municipal health services, and local aging-in-place strategies may represent a scalable approach to sustaining independence among aging populations. Prioritizing workforce training, referral pathways, community-health partnerships, and long-term funding mechanisms will be essential to achieving this integration sustainably. These findings suggest that community-based interventions relevant to public health practice may play an important role in maintaining independence among older adults, particularly when they are context-sensitive, culturally responsive, and integrated within existing health and social care systems.

### 4.1 Implications for practice

Findings emphasize the value of tailored community-based interventions in supporting physical, cognitive, and emotional well-being among older adults. Physical activity programs, including tai chi, cognitive training, dance-based interventions such as BAILAMOS© (84, 88, 95), and multidomain approaches, demonstrated reductions in fall risk, improvements in mobility, and benefits for mental health outcomes (7, 18, 41, 54, 55, 82). Programs that were low-cost, group-based, and embedded within existing community infrastructures appeared particularly feasible for broader implementation. Nutrition and health education interventions were more sustainable when they incorporated family involvement, cultural tailoring, and interactive or empowerment-oriented components, supporting sustained behavior change (30, 64, 65, 67). Similarly, social support initiatives, including peer-led and community-based models, were associated with reductions in loneliness and increased engagement, particularly when locally embedded and culturally aligned (19, 77, 91, 93, 96, 103, 104). Rehabilitation and supportive care interventions delivered by multidisciplinary teams were associated with improvements in functional independence, reduced hospitalizations, and enhanced quality of life (13, 16, 69, 70, 72, 74, 75, 102). Their long-term sustainability may depend on integration into existing health and social care systems, stable funding mechanisms, and adequate workforce capacity.

### 4.2 Future research directions and recommendations

Across the included studies, several consistent research gaps were identified. In physical activity interventions, many studies recommended larger and longer trials to assess sustained effects on mobility, falls self-efficacy, and cognitive outcomes (11, 37, 79, 80). Several authors emphasized the need to integrate mental health components within physical activity programs and to examine subgroup differences, particularly among frailer adults and those with depressive symptoms (27, 81). Additional priorities included evaluating culturally adapted models, combined nutrition and exercise strategies for sarcopenic obesity (39, 41, 65), and community-based fall prevention approaches (26, 63).

In cognitive and psychological interventions, long-term follow-up was frequently recommended to determine the durability of memory and cognitive benefits, especially among populations with mild cognitive impairment and those at risk of dementia (97). Studies highlighted the need to combine cognitive and physical training, expand culturally adapted peer-delivered models, and test integrated cognitive behavioral approaches in underserved populations (16, 17, 28, 45, 49, 53). Multidomain interventions were often accompanied by calls for larger RCTs examining combined physical, cognitive, and social components over extended follow-up periods (18, 55).

Health education and chronic disease management interventions identified the need for further evaluation of healthcare utilization, sustained behavior change, and culturally adapted or peer-led delivery models (4, 58, 66, 68, 100). Several trials also recommended examining the role of digital tools in enhancing accessibility and adherence, including telehealth platforms, mobile applications, and blended delivery formats, with particular attention to family and community involvement (29, 59, 60, 62, 63, 101).

### 4.3 Limitations

This systematic review has several limitations. First, restricting inclusion to English-language publications may have introduced language bias and excluded relevant evidence published in other languages. Second, although limiting the review to RCTs strengthened internal validity, this decision excluded observational and qualitative studies that could have provided important contextual insights into implementation processes, participant experiences, and real-world feasibility. In addition, methodological quality varied across included studies, including potential risks related to allocation concealment, blinding, attrition, and selective outcome reporting, which may have influenced effect estimates. Relatedly, the decision to exclude blinding domains (D4 and D5) from the overall study-level risk-of-bias classification may have resulted in a more favorable quality profile than would have been obtained using stricter composite approaches, and this should be considered when interpreting the overall distribution of risk-of-bias ratings. Third, substantial heterogeneity in outcome measures, intervention designs, and follow-up durations posed challenges for synthesis and limited comparability across studies. Independence was operationalized in diverse ways, ranging from objective physical performance measures to self-reported quality-of-life and psychosocial indicators, complicating the direct aggregation and interpretation of findings. Publication bias also remains a concern because trials with statistically significant findings are more likely to be published. As a result, null or negative studies may be underrepresented, potentially inflating estimates of effectiveness. Fourth, the predominance of studies conducted in high-income countries limits the geographic diversity of the evidence base and may constrain transferability to low- and middle-income settings where community infrastructure, workforce capacity, service integration, and resource availability differ substantially. The relative underrepresentation of studies from lower-resource contexts restricts global generalizability, particularly in regions undergoing rapid demographic transition with distinct social support structures and health system configurations. In addition, many trials recruited community-dwelling older adults who were willing and able to participate in structured programs, which may reduce applicability to more socially isolated, cognitively impaired, or severely frail populations. Fifth, the limited follow-up duration in many studies constrains conclusions regarding the sustainability of intervention effects. Few trials assessed hard endpoints such as institutionalization, hospitalization, or mortality, thereby limiting insight into broader system-level impact. Sixth, variation in intervention intensity, dosage, delivery models, and contextual adaptations complicates the identification of optimal parameters for success. Limited reporting on implementation fidelity and adherence further constrains understanding of whether observed outcomes reflect the intervention design or delivery variability. Incomplete reporting of intervention components, community engagement processes, and resource requirements restricts replicability and limits assessment of cost-effectiveness and scalability in routine practice. In addition, conclusions regarding scalability and sustainability should be interpreted cautiously, as these dimensions were inconsistently or explicitly reported across trials and sometimes inferred from intervention characteristics, delivery models, and follow-up descriptions. Finally, underreporting of contextual and structural constraints may obscure barriers to implementation and limit understanding of how interventions perform across diverse real-world settings.

Considering these limitations, future research should prioritize pragmatic trials that evaluate long-term sustainability, cost-effectiveness, and the integration of community-based interventions within existing health and social care systems. In addition, greater attention is needed to evaluate the effectiveness of such interventions in low- and middle-income settings and among socially vulnerable older populations, where structural barriers may limit access to community-based services.

## 5 Conclusion

This systematic review synthesizes evidence from RCTs evaluating community-based interventions designed to support independence among older adults. Across diverse national contexts and intervention models, programs targeting physical activity, cognitive engagement, health education, nutrition, social participation, and restorative approaches were generally associated with improvements in functional capacity, psychological wellbeing, and social connectedness. Together, these domains may contribute to independence in later life, with functional capacity more directly related to autonomy and psychosocial outcomes acting mainly as indirect contributors.

Although several trials reported null or mixed findings, the overall pattern of evidence suggests that community-based approaches may support multiple determinants of independence in later life. The findings suggest that community-based delivery is not merely an alternative implementation setting but may provide a practical platform for integrating the physical, cognitive, and social dimensions of healthy aging. Interventions that were structured, culturally responsive, and embedded within existing community infrastructures appeared particularly adaptable across contexts. Multidomain models that combined physical, cognitive, and social components appeared especially aligned with the interconnected nature of functional decline and resilience in aging populations.

In the context of rapid global population aging, community-level public health strategies that preserve functional ability and participation may represent an important component of sustainable aging policy. Advancing implementation research, strengthening integration with primary care and municipal systems, and extending follow-up durations will be important for sustaining and extending gains relevant to later-life independence.

## Data Availability

All data generated or analyzed during this study are included in this published article.

## References

[1] United Nations DoEaSA, Population Division. World Population Prospects 2022: Summary of Results. New York: United Nations (2022). p. 2022.

[2] Bureau. USC. Demographic Turning Points for the United States: Population Projections for 2020 to 2060. Washington, DC: U.S. Department of Commerce (2020).

[3] United Nations DoEaSA, Population Division. World Population Ageing 2019: Highlights. New York: United Nations (2019).

[4] Clark F Azen SP Zemke R Jackson J Carlson M Mandel D . Occupational therapy for independent-living older adults. A randomized controlled trial. JAMA. (1997) 278:1321–6. 9343462

[5] Stuck AE Minder CE Peter-Wüest I Gillmann G Egli C Kesselring A . A randomized trial of in-home visits for disability prevention in community-dwelling older people at low and high risk for nursing home admission. Arch Intern Med. (2000) 160:977–86. doi: 10.1001/archinte.160.7.977, 10761963

[6] Fielding RA Rejeski WJ Blair S Church T Espeland MA Gill TM . The lifestyle interventions and Independence for elders study: design and methods. J Gerontol A Biol Sci Med Sci. (2011) 66:1226–37. doi: 10.1093/gerona/glr123, 21825283 PMC3193523

[7] Rejeski WJ Ambrosius WT Burdette JH Walkup MP Marsh AP. Community weight loss to combat obesity and disability in at-risk older adults. J Gerontol A Biol Sci Med Sci. (2017) 72:1547–53. doi: 10.1093/gerona/glw252, 28064148 PMC5861918

[8] Groessl EJ Kaplan RM Castro Sweet CM Church T Espeland MA Gill TM . Cost-effectiveness of the LIFE physical activity intervention for older adults at increased risk for mobility disability. J Gerontol A Biol Sci Med Sci. (2016) 71:656–62. doi: 10.1093/gerona/glw001, 26888433 PMC5007742

[9] Keall MD Pierse N Howden-Chapman P Guria J Cunningham CW Baker MG. Cost-benefit analysis of fall injuries prevented by a programme of home modifications: a cluster randomised controlled trial. Inj Prev. (2017) 23:22–6. doi: 10.1136/injuryprev-2015-041947, 27312961

[10] Cubanski J Neuman T. What to Know about Medicare Spending and Financing 2023 22.11.2024. Available online at: https://www.kff.org/medicare/issue-brief/what-to-know-about-medicare-spending-and-financing/ (Accessed October 1, 2025).

[11] Bann D Chen H Bonell C Glynn NW Fielding RA Manini T . Socioeconomic differences in the benefits of structured physical activity compared with health education on the prevention of major mobility disability in older adults: the LIFE study. J Epidemiol Community Health. (2016) 70:930–3. doi: 10.1136/jech-2016-207321, 27060177 PMC5013156

[12] Loh DA Hairi NN Choo WY Mohd Hairi F Peramalah D Kandiben S . Multicomponent exercise and theRApeutic lifestyle (CERgAS) intervention to improve physical performance and maintain independent living among urban poor older people--a cluster randomised controlled trial. BMC Geriatr. (2015) 15:8. doi: 10.1186/s12877-015-0002-7 25887235 PMC4334409

[13] Lamb SE Bruce J Hossain A Ji C Longo R Lall R . Screening and intervention to prevent falls and fractures in older people. N Engl J Med. (2020) 383:1848–59. doi: 10.1056/NEJMoa2001500, 33211928

[14] Gitlin LN Winter L Dennis MP Corcoran M Schinfeld S Hauck WW. A randomized trial of a multicomponent home intervention to reduce functional difficulties in older adults. J Am Geriatr Soc. (2006) 54:809–16. doi: 10.1111/j.1532-5415.2006.00703.x, 16696748

[15] Szanton SL Thorpe RJ Boyd C Tanner EK Leff B Agree E . Community aging in place, advancing better living for elders: a bio-behavioral-environmental intervention to improve function and health-related quality of life in disabled older adults. J Am Geriatr Soc. (2011) 59:2314–20. doi: 10.1111/j.1532-5415.2011.03698.x, 22091738 PMC3245364

[16] Guerrero LR Menkin JA Carrillo CA Reyes CE Trejo L Banks C . Community-partnered evaluation of the aging mastery program in Los Angeles area senior centers. Health Educ Behav. (2020) 47:57–66. doi: 10.1177/1090198119882992, 31630566 PMC7643363

[17] Feng L Romero-Garcia R Suckling J Tan J Larbi A Cheah I . Effects of choral singing versus health education on cognitive decline and aging: a randomized controlled trial. Aging (Albany NY). (2020) 12:24798–816. doi: 10.18632/aging.202374, 33346748 PMC7803497

[18] Lee S Harada K Bae S Harada K Makino K Anan Y . A non-pharmacological multidomain intervention of dual-task exercise and social activity affects the cognitive function in community-dwelling older adults with mild to moderate cognitive decline: a randomized controlled trial. Front Aging Neurosci. (2023) 15:1005410. doi: 10.3389/fnagi.2023.1005410, 36993908 PMC10040752

[19] Reid KF Laussen J Bhatia K Englund DA Kirn DR Price LL . Translating the lifestyle interventions and Independence for elders clinical trial to older adults in a real-world community-based setting. J Gerontol A Biol Sci Med Sci. (2019) 74:924–8. doi: 10.1093/gerona/gly152, 30010808 PMC6521918

[20] Page MJ McKenzie JE Bossuyt PM Boutron I Hoffmann TC Mulrow CD . The PRISMA 2020 statement: an updated guideline for reporting systematic reviews. BMJ. (2021) 372:n71. doi: 10.1136/bmj.n71, 33782057 PMC8005924

[21] ExpandNet. WHO. Nine steps for developing a scaling-up strategy. Geneva: World Health Organization. (2010). Available online at: https://www.who.int/publications/i/item/9789241500319 (Accessed October 1, 2025).

[22] Fielding RA Guralnik JM King AC Pahor M McDermott MM Tudor-Locke C . Dose of physical activity, physical functioning and disability risk in mobility-limited older adults: results from the LIFE study randomized trial. PLoS One. (2017) 12:e0182155. doi: 10.1371/journal.pone.0182155, 28820909 PMC5562326

[23] Stuck AE Aronow HU Steiner A Alessi CA Büla CJ Gold MN . A trial of annual in-home comprehensive geriatric assessments for elderly people living in the community. N Engl J Med. (1995) 333:1184–9. doi: 10.1056/NEJM199511023331805, 7565974

[24] Johnson S McLeod B Gupta S McLeod K. Impact of a home-based nutrition and exercise intervention in improving functional capacity associated with falls among rural seniors in Canada. Qual Ageing Older Adults. (2018) 19:261–72. doi: 10.1108/qaoa-11-2017-0044

[25] Pahor M Blair SN Espeland M Fielding R Gill TM Guralnik JM . Effects of a physical activity intervention on measures of physical performance: results of the lifestyle interventions and independence for elders pilot (LIFE-P) study. J Gerontol A Biol Sci Med Sci. (2006) 61:1157–65. doi: 10.1093/gerona/61.11.1157, 17167156

[26] Shumway-Cook A Silver IF LeMier M York S Cummings P Koepsell TD. Effectiveness of a community-based multifactorial intervention on falls and fall risk factors in community-living older adults: a randomized, controlled trial. J Gerontol A Biol Sci Med Sci. (2007) 62:1420–7. doi: 10.1093/gerona/62.12.1420, 18166695

[27] Hernandez R Andrade FCD Piedra LM Tabb KM Xu S Sarkisian C. The impact of exercise on depressive symptoms in older Hispanic/Latino adults: results from the '¡Caminemos!' study. Aging Ment Health. (2019) 23:680–5. doi: 10.1080/13607863.2018.1450833, 29608340 PMC6494707

[28] Janevic M Robinson-Lane SG Courser R Brines E Hassett AL. A community health worker-led positive psychology intervention for African American older adults with chronic pain. Gerontologist. (2022) 62:1369–80. doi: 10.1093/geront/gnac010, 35394525 PMC9579460

[29] Uemura K Yamada M Okamoto H. Effects of active learning on health literacy and behavior in older adults: a randomized controlled trial. J Am Geriatr Soc. (2018) 66:1721–9. doi: 10.1111/jgs.15458, 30019768

[30] Wu MP Wu SV Lee MC Peng LN Tsao LI Lee WJ. Health-promotion interventions enhance and maintain self-efficacy for adults at cardiometabolic risk: a randomized controlled trial. Arch Gerontol Geriatr. (2019) 82:61–6. doi: 10.1016/j.archger.2019.01.009, 30716679

[31] Barker TH Stone JC Sears K Klugar M Tufanaru C Leonardi-Bee J . The revised JBI critical appraisal tool for the assessment of risk of bias for randomized controlled trials. JBI Evid Synth. (2023) 21:494–506. doi: 10.11124/JBIES-22-00430, 36727247

[32] Boutron I Moher D Altman D Schulz K Ravaud P. Extending the CONSORT statement to randomized trials of nonpharmacologic treatment: explanation and elaboration. Ann Intern Med. (2008) 148:295–309. doi: 10.7326/0003-4819-148-4-200802190-00008, 18283207

[33] Higgins JP Page MJ Elbers RG Sterne JAC. Chapter 8: assessing risk of bias in a randomized trial [last updated October 2019]. Cochrane Handbook for Systematic Reviews of Interventions Version 65. Avaialble online at: https://www.cochrane.org/authors/handbooks-and-manuals/handbook/current/chapter-08 (Accessed October 1, 2025).

[34] Thomas J Harden A. Methods for the thematic synthesis of qualitative research in systematic reviews. BMC Med Res Methodol. (2008) 8:45. doi: 10.1186/1471-2288-8-45, 18616818 PMC2478656

[35] Milat AJ King L Bauman AE Redman S. The concept of scalability: increasing the scale and potential adoption of health promotion interventions into policy and practice. Health Promot Int. (2013) 28:285–98. doi: 10.1093/heapro/dar097, 22241853

[36] Moore JE Mascarenhas A Bain J Straus SE. Developing a comprehensive definition of sustainability. Implement Sci. (2017) 12:110. doi: 10.1186/s13012-017-0637-1, 28865479 PMC5581411

[37] Arai T Obuchi S Inaba Y Nagasawa H Shiba Y Watanabe S . The effects of short-term exercise intervention on falls self-efficacy and the relationship between changes in physical function and falls self-efficacy in Japanese older people: a randomized controlled trial. Am J Phys Med Rehabil. (2007) 86:133–41. doi: 10.1097/PHM.0b013e31802ef29d, 17251695

[38] Giné-Garriga M Guerra M Unnithan VB. The effect of functional circuit training on self-reported fear of falling and health status in a group of physically frail older individuals: a randomized controlled trial. Aging Clin Exp Res. (2013) 25:329–36. doi: 10.1007/s40520-013-0048-3, 23740589

[39] Kim H Suzuki T Saito K Kim M Kojima N Ishizaki T . Effectiveness of exercise with or without thermal therapy for community-dwelling elderly Japanese women with non-specific knee pain: a randomized controlled trial. Arch Gerontol Geriatr. (2013) 57:352–9. doi: 10.1016/j.archger.2013.06.008, 23849900

[40] King AC Salvo D Banda JA Ahn DK Chapman JE Gill TM . Preserving older adults' routine outdoor activities in contrasting neighborhood environments through a physical activity intervention. Prev Med. (2017) 96:87–93. doi: 10.1016/j.ypmed.2016.12.049, 28039068 PMC5328783

[41] Marconcin P Yázigi F Teles J Campos P Espanha M. The effectiveness of a randomised clinical trial of PLE(2)NO self-management and exercise programme for knee osteoarthritis to improve self-efficacy. Musculoskelet Care. (2022) 20:137–44. doi: 10.1002/msc.1573, 34077602

[42] Murphy SL Strasburg DM Lyden AK Smith DM Koliba JF Dadabhoy DP . Effects of activity strategy training on pain and physical activity in older adults with knee or hip osteoarthritis: a pilot study. Arthritis Rheum. (2008) 59:1480–7. doi: 10.1002/art.24105, 18821646 PMC3046422

[43] Rubenstein LZ Aronow HU Schloe M Steiner A Alessi CA Yuhas KE . A home-based geriatric assessment, follow-up and health promotion program: design, methods, and baseline findings from a 3-year randomized clinical trial. Aging (Milano). (1994) 6:105–20. doi: 10.1007/BF03324224, 7918729

[44] Smith-Ray RL Makowski-Woidan B Hughes SL. A randomized trial to measure the impact of a community-based cognitive training intervention on balance and gait in cognitively intact black older adults. Health Educ Behav. (2014) 41:62s–9s. doi: 10.1177/1090198114537068, 25274713 PMC4326003

[45] Song D Yu DSF. Effects of a moderate-intensity aerobic exercise programme on the cognitive function and quality of life of community-dwelling elderly people with mild cognitive impairment: a randomised controlled trial. Int J Nurs Stud. (2019) 93:97–105. doi: 10.1016/j.ijnurstu.2019.02.019, 30901716

[46] Zhao X Ji C Zhang C Huang C Zhou Y Wang L. Transferability and sustainability of process-based multi-task adaptive cognitive training in community-dwelling older adults with mild cognitive impairment: a randomized controlled trial. BMC Psychiatry. (2023) 23:418. doi: 10.1186/s12888-023-04917-3, 37308857 PMC10259063

[47] Liao SJ Tan MP Chong MC Chua YP. The impact of combined music and tai chi on depressive symptoms among community-dwelling older persons: a cluster randomized controlled trial. Issues Ment Health Nurs. (2018) 39:398–402. doi: 10.1080/01612840.2017.1417519, 29436896

[48] Clark PG Nigg CR Greene G Riebe D Saunders SD. The study of exercise and nutrition in older Rhode islanders (SENIOR): translating theory into research. Health Educ Res. (2002) 17:552–61. doi: 10.1093/her/17.5.552, 12408200

[49] Khodneva Y Richman J Andreae S Cherrington A Safford MM. Peer support intervention improves pain-related outcomes among rural adults with diabetes and chronic pain at 12-month follow-up. J Rural Health. (2021) 37:394–405. doi: 10.1111/jrh.12422, 32124499 PMC9724177

[50] Metzner G Horstmeier LM Bengel J Bitzer EM Dreher E Frank F . Local, collaborative, stepped, and personalized care management for older people with chronic diseases - results from the randomized controlled LoChro-trial. BMC Geriatr. (2023) 23:92. doi: 10.1186/s12877-023-03797-2, 36782119 PMC9924193

[51] Ng TP Nyunt MSZ Feng L Feng L Niti M Tan BY . Multi-domains lifestyle interventions reduces depressive symptoms among frail and pre-frail older persons: randomized controlled trial. J Nutr Health Aging. (2017) 21:918–26. doi: 10.1007/s12603-016-0867-y, 28972245 PMC12877681

[52] Piette JD Hampstead BM Marinec N Chen J Roberts JS. A pilot randomized trial of a purposeful and stimulating volunteer opportunity: program satisfaction and potential impacts on perceived cognitive change in a neurologically mixed sample of older adults. Alzheimer Dis Assoc Disord. (2023) 37:237–42. doi: 10.1097/WAD.0000000000000572, 37615487 PMC10454976

[53] Song D Yu D Liu T Wang J. Effect of an aerobic dancing program on sleep quality for older adults with mild cognitive impairment and poor sleep: a randomized controlled trial. J Am Med Dir Assoc. (2023). 25:494–9. doi: 10.1016/j.jamda.2023.09.020 39492163

[54] Liang CK Lee WJ Hwang AC Lin CS Chou MY Peng LN . Efficacy of multidomain intervention against physio-cognitive decline syndrome: a cluster-randomized trial. Arch Gerontol Geriatr. (2021) 95:104392. doi: 10.1016/j.archger.2021.104392, 33765656

[55] Meng X Su J Gao T Ma D Zhao Y Fang S . Multidomain interventions based on a life-course model to prevent dementia in at-risk Chinese older adults: a randomized controlled trial. Int J Nurs Stud. (2024) 152:104701. doi: 10.1016/j.ijnurstu.2024.104701, 38330865

[56] Spoorenberg SLW Wynia K Uittenbroek RJ Kremer HPH Reijneveld SA. Effects of a population-based, person-centred and integrated care service on health, wellbeing and self-management of community-living older adults: a randomised controlled trial on embrace. PLoS One. (2018) 13:e0190751. doi: 10.1371/journal.pone.0190751, 29351295 PMC5774687

[57] Szanton SL Wolff JW Leff B Thorpe RJ Tanner EK Boyd C . CAPABLE trial: a randomized controlled trial of nurse, occupational therapist and handyman to reduce disability among older adults: rationale and design. Contemp Clin Trials. (2014) 38:102–12. doi: 10.1016/j.cct.2014.03.005, 24685996 PMC4074085

[58] Ćwirlej-Sozańska A Wiśniowska-Szurlej A Wilmowska-Pietruszyńska A Drużbicki M Sozański B Wołoszyn N . Evaluation of the effect of 16 weeks of multifactorial exercises on the functional fitness and postural stability of a low-income elderly population. Top Geriatr Rehabil. (2018) 34:251–61. doi: 10.1097/tgr.0000000000000202

[59] Kohn JN Lobo JD Troyer EA Wilson KL Ang G Walker AL . Tai chi or health education for older adults with hypertension: effects on mental health and psychological resilience to COVID-19. Aging Ment Health. (2023) 27:496–504. doi: 10.1080/13607863.2022.2053836, 35311437 PMC9489818

[60] Mitchell RE Ash SL McClelland JW. Nutrition education among low-income older adults: a randomized intervention trial in congregate nutrition sites. Health Educ Behav. (2006) 33:374–92. doi: 10.1177/1090198105276212, 16699126

[61] Park YH Song M Cho BL Lim JY Song W Kim SH. The effects of an integrated health education and exercise program in community-dwelling older adults with hypertension: a randomized controlled trial. Patient Educ Couns. (2011) 82:133–7. doi: 10.1016/j.pec.2010.04.002, 20434864

[62] Sugiyama T Steers WN Wenger NS Duru OK Mangione CM. Effect of a community-based diabetes self-management empowerment program on mental health-related quality of life: a causal mediation analysis from a randomized controlled trial. BMC Health Serv Res. (2015) 15:115. doi: 10.1186/s12913-015-0779-2, 25880234 PMC4375843

[63] Woo SC Chen MY Chen LK Liu CY. Effectiveness of resistance band use in conjunction with tai chi among older adults with Prefrailty to improve functional fitness, quality of life, and heart rate variability. J Gerontol Nurs. (2024) 50:19–26. doi: 10.3928/00989134-20240416-04, 38691121

[64] Endevelt R Lemberger J Bregman J Kowen G Berger-Fecht I Lander H . Intensive dietary intervention by a dietitian as a case manager among community dwelling older adults: the EDIT study. J Nutr Health Aging. (2011) 15:624–30. doi: 10.1007/s12603-011-0074-9, 21968856 PMC12878861

[65] Kim H Kim M Kojima N Fujino K Hosoi E Kobayashi H . Exercise and nutritional supplementation on community-dwelling elderly Japanese women with Sarcopenic obesity: a randomized controlled trial. J Am Med Dir Assoc. (2016) 17:1011–9. doi: 10.1016/j.jamda.2016.06.016, 27544583

[66] Lu CH Tang ST Lei YX Zhang MQ Lin WQ Ding SH . Community-based interventions in hypertensive patients: a comparison of three health education strategies. BMC Public Health. (2015) 15:33. doi: 10.1186/s12889-015-1401-6, 25631224 PMC4314804

[67] Moore-Harrison TL Speer EM Johnson FT Cress ME. The effects of aerobic training and nutrition education on functional performance in low socioeconomic older adults. J Geriatr Phys Ther. (2008) 31:18–23. doi: 10.1519/00139143-200831010-00004, 18489804

[68] Wang L Xu X Zhang Y Hao H Chen L Su T . A model of health education and management for osteoporosis prevention. Exp Ther Med. (2016) 12:3797–805. doi: 10.3892/etm.2016.3822, 28105113 PMC5228464

[69] Ekelund C Eklund K. Longitudinal effects on self-determination in the RCT “continuum of care for frail elderly people”. Quality in Ageing and Older Adults. (2015) 16:165–76. doi: 10.1108/qaoa-12-2014-0045

[70] Evans CJ Bone AE Yi D Gao W Morgan M Taherzadeh S . Community-based short-term integrated palliative and supportive care reduces symptom distress for older people with chronic noncancer conditions compared with usual care: a randomised controlled single-blind mixed method trial. Int J Nurs Stud. (2021) 120:103978. doi: 10.1016/j.ijnurstu.2021.103978, 34146843

[71] González-Guerrero JL Alonso-Fernández T García-Mayolín N Gusi N Ribera-Casado JM. Effectiveness of a follow-up program for elderly heart failure patients after hospital discharge. A randomized controlled trial. Eur Geriatric Med. (2014) 5:252–7. doi: 10.1016/j.eurger.2014.05.005

[72] Shvedko AV Thompson JL Greig CA Whittaker AC. Physical activity intervention for loneliness (PAIL) in community-dwelling older adults: a randomised feasibility study. Pilot Feasibility Stud. (2020) 6:73. doi: 10.1186/s40814-020-00587-0, 32489675 PMC7245022

[73] Wong AKC Wong FKY Chow KKS Wong SM Bayuo J Ho AKY. Effect of a Mobile health application with nurse support on quality of life among community-dwelling older adults in Hong Kong: a randomized clinical trial. JAMA Netw Open. (2022) 5:e2241137. doi: 10.1001/jamanetworkopen.2022.41137, 36350651 PMC9647479

[74] Wong AKC Wong FKY Ngai JSC Hung SYK Li WC. Effectiveness of a health-social partnership program for discharged non-frail older adults: a pilot study. BMC Geriatr. (2020) 20:339. doi: 10.1186/s12877-020-01722-5, 32912218 PMC7488104

[75] Xu J Yang F Si L Qian D. Do integrated health care interventions improve well-being among older adults with hypertension? Evidence from rural China. Soc Indic Res. (2022) 160:825–43. doi: 10.1007/s11205-020-02482-w

[76] Acton JH Molik B Court H Margrain TH. Effect of a home visit-based Low vision rehabilitation intervention on visual function outcomes: an exploratory randomized controlled trial. Invest Ophthalmol Vis Sci. (2016) 57:6662–7. doi: 10.1167/iovs.16-19901, 27930779

[77] Nikolaus T Bach M. Preventing falls in community-dwelling frail older people using a home intervention team (HIT): results from the randomized Falls-HIT trial. J Am Geriatr Soc. (2003) 51:300–5. doi: 10.1046/j.1532-5415.2003.51102.x, 12588572

[78] Reed RL Roeger L Howard S Oliver-Baxter JM Battersby MW Bond M . A self-management support program for older Australians with multiple chronic conditions: a randomised controlled trial. Med J Aust. (2018) 208:69–74. doi: 10.5694/mja17.00127, 29385967

[79] Brown JD Lo-Ciganic WH Shao H Pahor M Manini TM. Trajectories of short physical performance battery are strongly associated with future major mobility disability: results from the LIFE study. J Clin Med. (2020) 9:2332. doi: 10.3390/jcm9082332, 32707877 PMC7465072

[80] Chen PY Song CY Yen HY Lin PC Chen SR Lu LH . Impacts of tai chi exercise on functional fitness in community-dwelling older adults with mild degenerative knee osteoarthritis: a randomized controlled clinical trial. BMC Geriatr. (2021) 21:449. doi: 10.1186/s12877-021-02390-9, 34332537 PMC8325845

[81] Jones CA Siever J Knuff K Van Bergen C Mick P Little J . Walk, talk and listen: a pilot randomised controlled trial targeting functional fitness and loneliness in older adults with hearing loss. BMJ Open. (2019) 9:e026169. doi: 10.1136/bmjopen-2018-026169, 30987987 PMC6500300

[82] King AC Campero MI Garcia D Blanco-Velazquez I Banchoff A Fierros F . Testing the effectiveness of community-engaged citizen science to promote physical activity, foster healthier neighborhood environments, and advance health equity in vulnerable communities: the steps for change randomized controlled trial design and methods. Contemp Clin Trials. (2021) 108:106526. doi: 10.1016/j.cct.2021.106526, 34371162 PMC8453124

[83] King AC Friedman R Marcus B Castro C Napolitano M Ahn D . Ongoing physical activity advice by humans versus computers: the community health advice by telephone (CHAT) trial. Health Psychol. (2007) 26:718–27. doi: 10.1037/0278-6133.26.6.718, 18020844

[84] Marquez DX Wilbur J Hughes SL Berbaum ML Wilson RS Buchner DM . B.A.I.L.a. - a Latin dance randomized controlled trial for older Spanish-speaking Latinos: rationale, design, and methods. Contemp Clin Trials. (2014) 38:397–408. doi: 10.1016/j.cct.2014.06.012, 24969395 PMC4123962

[85] Marquez DX Wilson R Aguiñaga S Vásquez P Fogg L Yang Z . Regular Latin dancing and health education may improve cognition of late middle-aged and older Latinos. J Aging Phys Act. (2017) 25:482–9. doi: 10.1123/japa.2016-0049, 28095105 PMC5515671

[86] Martín-Valero R Cuesta-Vargas AI Labajos-Manzanares MT. Effectiveness of the physical activity promotion programme on the quality of life and the cardiopulmonary function for inactive people: randomized controlled trial. BMC Public Health. (2013) 13:127. doi: 10.1186/1471-2458-13-127, 23399032 PMC3575357

[87] Oh SL Kim DY Bae JH Lim JY. Effects of rural community-based integrated exercise and health education programs on the mobility function of older adults with knee osteoarthritis. Aging Clin Exp Res. (2021) 33:3005–14. doi: 10.1007/s40520-020-01474-7, 32020485

[88] Oh SL Kim HJ Woo S Cho BL Song M Park YH . Effects of an integrated health education and elastic band resistance training program on physical function and muscle strength in community-dwelling elderly women: healthy aging and happy aging II study. Geriatr Gerontol Int. (2017) 17:825–33. doi: 10.1111/ggi.12795, 27138245

[89] Piedra LM Andrade FCD Hernandez R Trejo L Prohaska TR Sarkisian CA. Let's walk! Age reattribution and physical activity among older Hispanic/Latino adults: results from the ¡Caminemos! Randomized trial. BMC Public Health. (2018) 18:964. doi: 10.1186/s12889-018-5850-6, 30075709 PMC6090751

[90] Quach J Theou O Pérez-Zepeda MU Godin J Rockwood K Kehler DS. Effect of a physical activity intervention and frailty on frailty trajectory and major mobility disability. J Am Geriatr Soc. (2022) 70:2915–24. doi: 10.1111/jgs.17941, 35779276

[91] Sheffield C Smith CA Becker M. Evaluation of an agency-based occupational therapy intervention to facilitate aging in place. Gerontologist. (2013) 53:907–18. doi: 10.1093/geront/gns145, 23213082

[92] Smail E Kaufmann C Anton S Manini T. Older adults with clinically relevant depressive symptoms have equal mobility benefit from a chronic physical activity intervention. Ment Health Phys Act. (2023) 25:100549. doi: 10.1016/j.mhpa.2023.100549

[93] Taylor SJ Carnes D Homer K Kahan BC Hounsome N Eldridge S . Novel three-day, community-based, nonpharmacological group intervention for chronic musculoskeletal pain (COPERS): a randomised clinical trial. PLoS Med. (2016) 13:e1002040. doi: 10.1371/journal.pmed.1002040, 27299859 PMC4907437

[94] Vaz Fragoso CA Miller ME King AC Kritchevsky SB Liu CK Myers VH . Effect of structured physical activity on sleep-wake behaviors in sedentary elderly adults with mobility limitations. J Am Geriatr Soc. (2015) 63:1381–90. doi: 10.1111/jgs.13509, 26115386 PMC4892176

[95] Wolf SL Barnhart HX Kutner NG McNeely E Coogler C Xu T. Reducing frailty and falls in older persons: an investigation of tai chi and computerized balance training. Atlanta FICSIT group. Frailty and injuries: cooperative studies of intervention techniques. J Am Geriatr Soc. (1996) 44:489–97. doi: 10.1111/j.1532-5415.1996.tb01432.x, 8617895

[96] Yang C Zhao F Xie C Zhang Y Dou Z Wei X. Community-based group rehabilitation program for stroke patients with dysphagia on quality of life, depression symptoms, and swallowing function: a randomized controlled trial. BMC Geriatr. (2023) 23:876. doi: 10.1186/s12877-023-04555-0, 38124046 PMC10731687

[97] Bae S Lee S Lee S Jung S Makino K Harada K . The effect of a multicomponent intervention to promote community activity on cognitive function in older adults with mild cognitive impairment: a randomized controlled trial. Complement Ther Med. (2019) 42:164–9. doi: 10.1016/j.ctim.2018.11.011, 30670238

[98] Morone NE Greco CM Moore CG Rollman BL Lane B Morrow LA . A mind-body program for older adults with chronic Low Back pain: a randomized clinical trial. JAMA Intern Med. (2016) 176:329–37. doi: 10.1001/jamainternmed.2015.8033, 26903081 PMC6361386

[99] Shake MC Crandall KJ Mathews RP Falls DG Dispennette AK. Efficacy of Bingocize(®): a game-centered Mobile application to improve physical and cognitive performance in older adults. Games Health J. (2018) 7:253–61. doi: 10.1089/g4h.2017.0139, 30089018

[100] Chao J Wang Y Xu H Yu Q Jiang L Tian L . The effect of community-based health management on the health of the elderly: a randomized controlled trial from China. BMC Health Serv Res. (2012) 12:449. doi: 10.1186/1472-6963-12-449, 23217036 PMC3537545

[101] Estebsari F Dastoorpoor M Mostafaei D Khanjani N Khalifehkandi ZR Foroushani AR . Design and implementation of an empowerment model to prevent elder abuse: a randomized controlled trial. Clin Interv Aging. (2018) 13:669–79. doi: 10.2147/CIA.S158097, 29713151 PMC5909776

[102] Jeon YH Simpson JM Low LF Woods R Norman R Mowszowski L . A pragmatic randomised controlled trial (RCT) and realist evaluation of the interdisciplinary home-bAsed Reablement program (I-HARP) for improving functional independence of community dwelling older people with dementia: an effectiveness-implementation hybrid design. BMC Geriatr. (2019) 19:199. doi: 10.1186/s12877-019-1216-x, 31357949 PMC6664757

[103] Eklund K Sjöstrand J Dahlin-Ivanoff S. A randomized controlled trial of a health-promotion programme and its effect on ADL dependence and self-reported health problems for the elderly visually impaired. Scand J Occup Ther. (2008) 15:68–74. doi: 10.1080/11038120701442963, 17852958

[104] Markle-Reid M Weir R Browne G Roberts J Gafni A Henderson S. Health promotion for frail older home care clients. J Adv Nurs. (2006) 54:381–95. doi: 10.1111/j.1365-2648.2006.03817.x, 16629922

[105] Parial LL Kor PPK Sumile EF Leung AYM. Dual-task Zumba Gold for improving the cognition of people with mild cognitive impairment: a pilot randomized controlled trial. Gerontologist. (2023) 63:1248–61. doi: 10.1093/geront/gnac081, 35679826

[106] Song D Yu D Liu T Wang J. Effect of an aerobic dancing program on sleep quality for older adults with mild cognitive impairment and poor sleep: a randomized controlled trial. J Am Med Dir Assoc. (2024) 25:494–9. doi: 10.1016/j.jamda.2023.09.020, 39492163

[107] Bevilacqua R Soraci L Stara V Riccardi GR Corsonello A Pelliccioni G . A systematic review of multidomain and lifestyle interventions to support the intrinsic capacity of the older population. Front Med (Lausanne). (2022) 9:929261. doi: 10.3389/fmed.2022.929261, 35911409 PMC9335156

[108] Motamed-Jahromi M Kaveh MH. Effective interventions on improving elderly's independence in activity of daily living: a systematic review and logic model. Front Public Health. (2021) 8:516151. doi: 10.3389/fpubh.2020.516151 33659228 PMC7917261

[109] Jadczak AD Makwana N Luscombe-Marsh N Visvanathan R Schultz TJ. Effectiveness of exercise interventions on physical function in community-dwelling frail older people: an umbrella review of systematic reviews. JBI Database System Rev Implement Rep. (2018) 16:752–75. doi: 10.11124/JBISRIR-2017-003551, 29521871

[110] Sherrington C Fairhall NJ Wallbank GK Tiedemann A Michaleff ZA Howard K . Exercise for preventing falls in older people living in the community. Cochrane Database Syst Rev. (2019) 1:Cd012424. doi: 10.1002/14651858.CD012424.pub2, 30703272 PMC6360922

[111] Ngandu T Lehtisalo J Solomon A Levälahti E Ahtiluoto S Antikainen R . A 2 year multidomain intervention of diet, exercise, cognitive training, and vascular risk monitoring versus control to prevent cognitive decline in at-risk elderly people (FINGER): a randomised controlled trial. Lancet. (2015) 385:2255–63. doi: 10.1016/s0140-6736(15)60461-5, 25771249

[112] Crocker TF Ensor J Lam N Jordão M Bajpai R Bond M . Community based complex interventions to sustain independence in older people: systematic review and network meta-analysis. BMJ. (2024) 384:e077764. doi: 10.1136/bmj-2023-077764, 38514079 PMC10955723

[113] Elhag S Niechcial MA Potter L Gow AJ. Exploring the impact of community-based interventions on healthy older adults' physical health, psychological wellbeing, and social connections: a systematic review. Ageing Res Rev. (2025) 111:102784. doi: 10.1016/j.arr.2025.102784, 40480291

