# Supplementary material for: Community-based interventions to support aging in place and functional independence in older adults: a systematic review of randomized controlled trials
Source: Front Public Health. 2026 May 15;14:1828271. doi: 10.3389/fpubh.2026.1828271 (PMC13219341; doi:10.3389/fpubh.2026.1828271)
Supplement: Supplementary file 1 [file Table_1.DOCX]

**Supplementary Table 1. Detailed characteristics of included randomized controlled trials.**

| **Author(s), Year, Country** | **Aim of the Study** | **Study Design** | **Population** | **Sample Size** | **Interventions** |
| --- | --- | --- | --- | --- | --- |
| Acton et al., 2016 United Kingdom | To examine the effect of a home visit–based visual rehabilitation intervention on self-reported visual function, depression, well-being, loneliness, adjustment to visual loss, and generic health-related quality of life. | Individual RCT (parallel), assessor-masked | Elderly individuals with low vision | N randomized = 67 (I = 35; C = 32) | **Name:** Home visit–based visual rehabilitation  **Category:** Occupational therapy, home modification, or reablement  **Description:** Functional vision training, emotional support, personal hygiene, medication management, kitchen safety, household tasks, and mobility.  **Intervention duration:** 6 months  **Follow-up:** 6 months |
| Arai et al., 2007 Japan | To evaluate the effects of a short-term exercise intervention on falls self-efficacy and its relationship with changes in physical function in older people. | Individual RCT (parallel), assessor-blinded | Community-dwelling Japanese older adults aged 65+ | N randomized = 171 (I = 85; C = 86) | **Name:** Short-term exercise intervention  **Category:** Physical activity or exercise  **Description:** 3-month program focusing on strength, balance, and functional training with warm-up and cool-down.  **Intervention duration:** 3 months  **Follow-up:** 3 months |
| Bae et al., 2019 Japan | To examine the effectiveness of a multicomponent intervention combining physical, cognitive, and social activities in improving cognitive function in older adults with MCI. | Individual RCT (parallel), assessor-blinded | Community-dwelling Japanese older adults with MCI, aged 65+ | N randomized = 83 (I = 41; C = 42) | **Name:** Multicomponent intervention program  **Category:** Multidomain or multicomponent  **Description:** 24-week group sessions including walking, TC, cognitive games, arts and crafts, and social events.  **Intervention duration:** 6 months  **Follow-up:** 6 months |
| Bann et al., 2016 United States | To evaluate whether the benefit of a structured PA intervention on reducing mobility disability differs by education or income. | Multicenter individual RCT (parallel) | Community-living older adults aged 70–89 at risk of mobility disability | N randomized = 1,635 [Included as a separate report from the LIFE trial due to distinct primary outcome.] | **Name:** LIFE Study Intervention  **Category:** Physical activity or exercise  **Description:** Moderate-intensity walking, strength, flexibility, and balance training.  **Intervention duration:** 31.2 months  **Follow-up:** 31 months |
| Brown et al., 2020 United States | To identify trajectories of SPPB changes and evaluate their predictive validity for future MMD. | Multicenter individual RCT (parallel), assessor-blinded | Community-dwelling older adults aged 70–89 | N randomized = 1,635 [Included as a separate report from the LIFE trial due to distinct primary outcome.] | **Name:** LIFE Study PA Intervention  **Category:** Physical activity or exercise  **Description:** Center-based and home-based walking, strength, flexibility, and balance training.  **Intervention duration:** 31 months  **Follow-up:** 31 months |
| Chao et al., 2012 China | To evaluate the effects of community-based health management on the health of the elderly. | Individual RCT (parallel) | Elderly individuals aged 60+ in Nanjing, China | N randomized = 2,400 | **Name:** Community-based Health Management Program  **Category:** Multidomain or multicomponent  **Description:** 18-month program including diet advice, psychological support, exercise, education, and skills training.  **Intervention duration:** 18 months  **Follow-up:** 18 months |
| Chen et al., 2021 Taiwan | To investigate the impact of TC exercise on functional fitness in community-dwelling older adults with mild knee OA. | Individual RCT (parallel) | Community-dwelling older adults with mild knee OA | N randomized = 68 (I = 36; C = 32) | **Name:** TC Exercise  **Category:** Mind-body interventions including Tai Chi, mindfulness, or dance  **Description:** Twice weekly for 12 weeks; Sun-style TC for balance, strength, and flexibility.  **Intervention duration:** 3 months  **Follow-up:** 3 months |
| Clark et al., 1997 United States | To evaluate the effectiveness of preventive OT services tailored for multiethnic independent-living older adults. | Individual RCT (parallel) | Independent-living, culturally diverse older adults aged 60+ | N randomized = 361 (OT = 122; Social = 120; Control = 119) | **Name:** Preventive OT  **Category:** Occupational therapy, home modification, or reablement  **Description:** Group and individual sessions on health-promoting activities, safety, exercise, and adaptive strategies.  **Intervention duration:** 9 months  **Follow-up:** 9 months |
| Clark et al., 2002 United States | To investigate the relative effectiveness of a multiple-behavior intervention versus single-behavior interventions in older adults. | Factorial RCT (2×2) | Community-dwelling older adults aged 65+ in East Providence, Rhode Island | N randomized = 1,300 | **Name:** SENIOR Project  **Category:** Health education or self-management  **Description:** Stage-tailored manuals, newsletters, and telephone coaching over 12 months promoting exercise and nutrition.  **Intervention duration:** 12 months  **Follow-up:** Outcomes assessed at 12 and 24 months |
| Cwirlej-Sozanska et al., 2018 Poland | To evaluate the effect of a 16-week multifactorial exercise and health education program on functional fitness and postural stability of low-income elderly. | Individual RCT (parallel) | Low-income elderly aged 65–75 living in the community | N randomized = 50; N analyzed = 44 (I = 21; C = 23) | **Name:** Multifactorial Exercise and Health Education Program (MEE)  **Category:** Multidomain or multicomponent  **Description:** 16-week endurance, balance, resistance, and functional exercises combined with health education.  **Intervention duration:** 4 months  **Follow-up:** 4 months |
| Ekelund & Eklund, 2015 Sweden | To evaluate longitudinal effects of a Continuum of Care intervention on self-determination in daily life for frail elderly people. | Individual RCT (parallel) | Community-dwelling frail older persons aged 65+ with at least one chronic disease and dependence in at least one daily activity | N randomized = 158 | **Name:** Continuum of Care for Frail Elderly  **Category:** Integrated or person-centred care  **Description:** 12-month multi-professional team collaboration: frailty screening, geriatric assessment, care planning, and case manager follow-up.  **Intervention duration:** 12 months  **Follow-up:** 12 months |
| Eklund et al., 2008 Sweden | To compare a health-promotion program versus individual program on ADL dependence and self-reported health in elderly with visual impairment. | Individual RCT (parallel) | Elderly individuals with AMD living at home, aged 65+ | N randomized = 229; N analyzed = 131 | **Name:** Health-promotion program for elderly with visual impairment  **Category:** Health education or self-management  **Description:** 28-month group sessions led by occupational therapists on self-care, mobility, and communication.  **Intervention duration:** 28 months  **Follow-up:** 28 months |
| Endevelt et al., 2011 Israel | To determine the impact of an intensive nutritional intervention on health and nutritional status of malnourished community-dwelling older adults. | Individual RCT (parallel) | Community-dwelling older adults at nutritional risk | N randomized = 127 | **Name:** Intensive Dietary Intervention  **Category:** Nutritional intervention  **Description:** Individualized dietitian sessions over 6 months focusing on tailored nutritional treatment and dietary improvements.  **Intervention duration:** 6 months  **Follow-up:** 6 months |
| Estebsari et al., 2018 Iran | To design and implement an empowerment educational intervention to prevent elder abuse. | Individual RCT (parallel) | Older adults aged 60+ in Tehran, Iran | N randomized = 464 | **Name:** Empowerment Educational Intervention  **Category:** Health education or self-management  **Description:** 6-month group-based sessions to improve self-efficacy, social support, and health-promoting behaviors.  **Intervention duration:** 6 months  **Follow-up:** 18 months |
| Evans et al., 2021 United Kingdom | To evaluate the impact of short-term integrated palliative and supportive care for older people with chronic non-cancer conditions and frailty. | Individual RCT (parallel), assessor-blinded; mixed-methods | Older adults (≥75 years) with moderate to severe frailty and chronic non-cancer conditions | N randomized = 50 (Intervention = 24; Usual care = 26) | **Name:** Short-term Integrated Palliative and Supportive Care  **Category:** Integrated or person-centred care  **Description:** 12-week multidisciplinary team (GPs and community nurses) intervention focusing on symptom management.  **Intervention duration:** 3 months  **Follow-up:** 3 months |
| Feng et al., 2020 Singapore | To examine the effect of choral singing on cognitive decline in aging. | Individual RCT (parallel) | Community-living elderly aged 60–84 years | N randomized = 93 | **Name:** Choral Singing Intervention  **Category:** Cognitive training [Note: also involves social and mind-body components]  **Description:** 24-month group singing sessions promoting cognitive exercises, social interaction, and musical training.  **Intervention duration:** 24 months  **Follow-up:** 24 months |
| Fielding et al., 2017 United States | To examine the effect of PA dose on physical functioning and onset of MMD in older adults with functional limitations. | Multicenter individual RCT (parallel), assessor-blinded | Sedentary men and women aged 70–89 with functional limitations | N randomized = 1,635 (LIFE trial cohort; analyzed as a distinct report with a separate primary outcome) | **Name:** Lifestyle Interventions and Independence for Elders (LIFE) Physical Activity Intervention  **Category:** Physical activity or exercise  **Description:** 24-month structured walking, resistance, and flexibility training with supervised classes and home-based goals.  **Intervention duration:** 24 months  **Follow-up:** 31 months |
| Giné-Garriga et al., 2013 Spain | To evaluate the effect of a 12-week functional circuit training program on fear of falling and health status in physically frail older individuals. | Individual RCT (parallel) | Physically frail community-dwelling older individuals aged 80–90 | N randomized = 51 (I = 26; C = 25) | **Name:** Functional Circuit Training (FCT)  **Category:** Physical activity or exercise  **Description:** Twice-weekly sessions for 12 weeks focusing on balance, strength, and functional exercises.  **Intervention duration:** 3 months  **Follow-up:** 9 months |
| Gitlin et al., 2006 United States | To test the efficacy of a multicomponent intervention to reduce functional difficulties, fear of falling, and home hazards, and enhance self-efficacy in older adults with chronic conditions. | Individual RCT (parallel) | Community-living adults aged 70+ with difficulty in one or more ADLs | N randomized = 319 | **Name:** Multicomponent Home-Based Intervention  **Category:** Occupational therapy, home modification, or reablement  **Description:** 6-month OT and physical therapy home visits covering home modification, problem-solving, energy conservation, fall recovery, and strength training.  **Intervention duration:** 6 months  **Follow-up:** 6 months |
| González-Guerrero et al., 2014 Spain | To evaluate the effectiveness of a disease management program for elderly HF patients after hospital discharge. | Individual RCT (parallel) | Elderly patients with acute HF discharged from the geriatric service | N randomized = 117 | **Name:** Disease Management Program (DMP)  **Category:** Integrated or person-centred care  **Description:** 12-month multidisciplinary follow-up in a geriatric day care hospital including health education, therapeutic control, and close follow-up.  **Intervention duration:** 12 months  **Follow-up:** 12 months |
| Groessl et al., 2016 United States | To examine the cost-effectiveness of a PA intervention in preventing MMD compared to a health education intervention. | Individual RCT (parallel) | Older adults aged 70–89 at risk for mobility disability | N randomized = 1,635 [Included as a separate report from the LIFE trial due to distinct primary outcome.] | **Name:** LIFE Study  **Category:** Physical activity or exercise  **Description:** Center-based walking, strength, flexibility, and balance training supplemented by home-based activity.  **Intervention duration:** 31.2 months  **Follow-up:** 31 months |
| Guerrero et al., 2020 United States | To evaluate the effectiveness of the Aging Mastery Program in improving quality of life, physical and mental health, and patient activation. | Wait-list RCT | Older adults attending five senior centers in Los Angeles | N randomized = 180 | **Name:** Aging Mastery Program (AMP)  **Category:** Health education or self-management  **Description:** 10-week weekly in-person classes on sleep, exercise, nutrition, medication management, and social engagement.  **Intervention duration:** 2.5 months  **Follow-up:** 2.5 months |
| Hernandez et al., 2019 United States | To examine the effects of an exercise intervention with or without attribution retraining on depressive symptoms in older Hispanic/Latino adults. | Individual RCT (parallel), double-blinded | Older Hispanic/Latino adults aged 60+ at senior centers in Los Angeles | N randomized = 572 | **Name:** ¡Caminemos!  **Category:** Physical activity or exercise  **Description:** 24-month group exercise classes with socialization and optional attribution retraining at senior centers.  **Intervention duration:** 24 months  **Follow-up:** 24 months |
| Janevic et al., 2022 United States | To assess the feasibility and preliminary effects of a culturally tailored chronic pain self-management intervention led by community health workers for African American older adults. | Pilot/feasibility RCT | African American older adults (mean age 72 years) with chronic musculoskeletal pain | N randomized = 46 (I = 22; C = 24) | **Name:** Positive STEPS  **Category:** Health education or self-management  **Description:** 7-week telephone sessions, web-based videos, and positive psychology activities (life review, goal setting).  **Intervention duration:** 1.75 months  **Follow-up:** 2 months |
| Johnson et al., 2018 Canada | To examine the impact of a home-based nutrition and exercise intervention on functional capacity to prevent falls among rural seniors. | Individual RCT (parallel) | Rural seniors aged 60+ | N randomized = 134 (Exercise = 40; Nutrition = 32; Exercise + Nutrition = 33; C = 29) | **Name:** Home Support Exercise Program with Ensure® supplementation  **Category:** Multidomain or multicomponent  **Description:** 6-month physiotherapist-delivered exercise and dietitian-delivered nutritional supplementation.  **Intervention duration:** 6 months  **Follow-up:** 6 months |
| Jones et al., 2019 Canada | To explore the impact of group exercise, socialization/health education, and audiological rehabilitation on physical function and loneliness in older adults with hearing loss. | Pilot/feasibility RCT | Ambulatory adults aged 65+ with self-reported hearing loss | N randomized = 66 (I = 35; C = 31) | **Name:** Walk, Talk and Listen (WTL)  **Category:** Physical activity or exercise  **Description:** 10-week YMCA program combining strength, resistance, communication strategies, and audiological rehabilitation.  **Intervention duration:** 2.5 months  **Follow-up:** 2.5 months |
| Keall et al., 2017 New Zealand | To assess the economic benefits of home modifications in preventing fall injuries. | Cluster RCT | Residents of owner-occupied homes built before 1980 with at least one occupant holding a community services card | N randomized = 1,850 households | **Name:** Home Injury Prevention Intervention  **Category:** Occupational therapy, home modification, or reablement  **Description:** 36-month installation of handrails, slip-resistant surfaces, and improved lighting to prevent falls.  **Intervention duration:** 36 months  **Follow-up:** 36 months |
| Khodneva et al., 2021 United States | To test the effect of a peer-led CBT intervention on pain self-efficacy, pain intensity, and pain-related functional limitations in adults with diabetes and chronic pain. | Cluster RCT | Rural adults with diabetes and chronic pain | N randomized = 230 | **Name:** Living Healthy Peer Support CBT Program  **Category:** Health education or self-management  **Description:** 12-week telephone-delivered peer CBT targeting pain and stress management integrated with diabetes self-management.  **Intervention duration:** 3 months  **Follow-up:** 12 months |
| Kim et al., 2013 Japan | To determine the effects of exercise with or without thermal therapy on knee pain, physical function, and quality of life in elderly women with chronic knee pain. | Individual RCT (parallel) | Community-dwelling elderly women aged ≥75 with non-specific knee pain | N randomized = 150; N analyzed = 137 (post-intervention) | **Name:** Exercise + Heat/Steam Generating Sheet (Ex + HSGS)  **Category:** Physical activity or exercise  **Description:** 3-month resistance and balance training combined with heat therapy using steam-generating sheets.  **Intervention duration:** 3 months  **Follow-up:** 3 months |
| Kim et al., 2016 Japan | To investigate the effects of exercise and/or nutritional supplementation on body composition, physical function, and blood components in elderly Japanese women with sarcopenic obesity. | Individual RCT (parallel) | Community-dwelling elderly Japanese women aged 70+ with sarcopenic obesity | N randomized = 139 | **Name:** Exercise and Nutrition (Ex + N)  **Category:** Multidomain or multicomponent  **Description:** 3-month progressive resistance and aerobic exercise with leucine-enriched amino acids and tea catechins.  **Intervention duration:** 3 months  **Follow-up:** 3 months |
| King et al., 2007 United States | To evaluate the effectiveness of telephone-based PA advice delivered by human counselors versus an automated system in promoting PA in older adults. | Individual RCT (parallel) | Initially inactive adults aged 55+ | N randomized = 218 | **Name:** Community Health Advice by Telephone (CHAT) Trial  **Category:** Physical activity or exercise  **Description:** 12-month individualized PA advice via human counselors or automated telephone system.  **Intervention duration:** 12 months  **Follow-up:** 12 months |
| King et al., 2017 United States | To evaluate the effects of neighborhood compactness on changes in routine outdoor activities among older adults. | Individual RCT (parallel) | Older adults aged 70–89 at increased mobility disability risk | N randomized = 400 | **Name:** Endurance-Strengthening PA Program  **Category:** Physical activity or exercise  **Description:** 12-month center-based and home-based aerobic walking, strength, flexibility, and balance training.  **Intervention duration:** 12 months  **Follow-up:** 12 months |
| King et al., 2021 United States | To test the effectiveness of combining a citizen science neighborhood intervention with a standard PA program in promoting PA and health equity in vulnerable communities. | Cluster RCT | Insufficiently active adults aged 40+ in or around affordable senior public housing | N randomized = 300 (ALED + Health Education = 140; ALED + Our Voice citizen science = 160) | **Name:** Our Voice + ALED  **Category:** Physical activity or exercise  **Description:** 24-month multi-level PA program using mobile app data collection, neighborhood assessments, and community advocacy.  **Intervention duration:** 24 months  **Follow-up:** 12 months |
| Kohn et al., 2023 United States | To compare the effectiveness of 12 weeks of TC versus health education on health and well-being in older adults with hypertension during COVID-19. | Individual RCT (parallel) | Community-dwelling older adults with hypertension | N randomized = 182; N analyzed = 131 | **Name:** Tai Chi (TC)  **Category:** Mind-body interventions including Tai Chi, mindfulness, or dance  **Description:** 12-week in-person group TC focusing on balance, flexibility, and mental health.  **Intervention duration:** 3 months  **Follow-up:** 3 months |
| Lamb et al., 2020 United Kingdom | To assess the clinical and cost-effectiveness of falls-risk screening followed by exercise or multifactorial intervention in older adults. | Pragmatic cluster RCT | Community-dwelling older adults aged 70+ | N randomized = 9,803 | **Name:** Otago Exercise Program and Multifactorial Fall Prevention  **Category:** Physical activity or exercise  **Description:** 6-month program with physical therapists delivering exercise and nurses delivering multifactorial fall prevention.  **Intervention duration:** 6 months  **Follow-up:** 18 months |
| Lee et al., 2023 Japan | To determine the impact of a multidomain program using dual-task exercise and social activities on cognitive function in older adults with mild to moderate cognitive decline. | Individual RCT (parallel) | Community-dwelling older adults aged 71–91 with mild to moderate cognitive decline | N randomized = 280 | **Name:** Dual-task Exercise and Social Activity Program  **Category:** Multidomain or multicomponent  **Description:** 10-month program combining cognitive tasks with physical exercises and social activities at community sports facilities.  **Intervention duration:** 10 months  **Follow-up:** 10 months |
| Liang et al., 2021 Taiwan | To investigate the efficacy of a community-based multidomain intervention among older adults with possible cognitive decline (PCD). | Cluster RCT | Community-dwelling older adults aged ≥65 with PCD across Taiwan | N randomized = 733 | **Name:** Multidomain Intervention  **Category:** Multidomain or multicomponent  **Description:** 12-month community-based program covering physical exercise, cognitive training, nutritional advice, and health education.  **Intervention duration:** 12 months  **Follow-up:** 12 months |
| Liao et al., 2018 China | To evaluate the effect of combined music and TC on depressive symptoms among community-dwelling older persons. | Cluster RCT | Community-dwelling older persons with mild to moderate depressive symptoms | N randomized = 107 (I = 55; C = 52) | **Name:** Combined Music and TC Therapy  **Category:** Mind-body interventions including Tai Chi, mindfulness, or dance  **Description:** 3-month program combining TC movements with relaxing Chinese folk music to promote mental and physical well-being.  **Intervention duration:** 3 months  **Follow-up:** 3 months |
| Loh et al., 2015 Malaysia | To evaluate the effectiveness of the CERgAS multicomponent exercise and lifestyle intervention in improving physical performance and independent living among older urban-poor adults. | Cluster RCT | Older adults aged 60+ from urban poor settings in Malaysia | N randomized = 164 (8 clusters) | **Name:** CERgAS  **Category:** Multidomain or multicomponent  **Description:** 6-week strength, balance, and flexibility exercises with nutrition education and oral care in low-cost public housing.  **Intervention duration:** 1.5 months  **Follow-up:** 6 months |
| Lu et al., 2015 China | To evaluate the effectiveness of community-based health education strategies in managing hypertensive patients with low socioeconomic status. | Individual RCT (parallel), non-blinded | Hypertensive patients aged 40–75 from Dongguan City, China | N randomized = 360 | **Name:** Interactive Education Workshop  **Category:** Health education or self-management  **Description:** 2-year interactive workshops with visual aids to improve hypertension knowledge, medication adherence, and lifestyle change.  **Intervention duration:** 24 months  **Follow-up:** 24 months |
| Marconcin et al., 2022 Portugal | To assess the effectiveness of a 12-week self-management and exercise intervention to improve self-efficacy in older individuals with KOA. | Individual RCT (parallel) | Individuals aged ≥60 with clinical and radiographic KOA | N randomized = 80 | **Name:** PLE2NO Self-Management and Exercise Programme  **Category:** Health education or self-management  **Description:** 12-week self-management strategies with physical fitness training to manage symptoms and promote healthy living.  **Intervention duration:** 3 months  **Follow-up:** 3 months |
| Markle-Reid et al., 2006 Canada | To evaluate the effects and costs of a proactive nursing health promotion intervention added to usual home care for older people. | Individual RCT (parallel), assessor-blinded | Older people aged ≥75 eligible for personal support services through home care | N randomized = 288; N analyzed = 242 (I = 120; C = 122); | **Name:** Proactive Nursing Health Promotion  **Category:** Health education or self-management  **Description:** 6-month home visits and telephone contacts by a registered nurse to provide health education and coordinate community services.  **Intervention duration:** 6 months  **Follow-up:** 6 months |
| Marquez et al., 2014 United States | To test the efficacy of a 4-month Latin dance program for improving lifestyle PA and health outcomes in older Latinos. | Individual RCT (parallel) | Latino/Hispanic older adults aged 55+ with low PA levels at risk for disability | N randomized = 332 (I = 166; C = 166) | **Name:** BAILAMOS©  **Category:** Mind-body interventions including Tai Chi, mindfulness, or dance  **Description:** 4-month twice-weekly Latin dance sessions (Merengue, Cha Cha Cha, Bachata, Salsa) with bi-monthly dance parties.  **Intervention duration:** 4 months  **Follow-up:** 4 months |
| Marquez et al., 2017 United States | To examine the impact of a culturally appropriate Latin dance program on cognitive function in older Latinos compared to health education. | Individual RCT (parallel) | Inactive older Latinos aged 55+ | N randomized = 57 | **Name:** BAILAMOS© Latin Dance Program  **Category:** Mind-body interventions including Tai Chi, mindfulness, or dance  **Description:** 4-month bilingual Latin dance program.  **Intervention duration:** 4 months  **Follow-up:** 4 months |
| Martín-Valero et al., 2013 Spain | To evaluate the effectiveness of the Physical Activity Promotion Program on quality of life and cardiopulmonary function in inactive individuals. | Individual RCT (parallel) | Inactive individuals aged 55+ with CV risk factors | N randomized = 100; N analyzed = 75 | **Name:** Physical Activity Promotion Program (PAPP)  **Category:** Physical activity or exercise  **Description:** 12-week twice-weekly structured exercise sessions tailored to individual capacity.  **Intervention duration:** 3 months  **Follow-up:** 3 months |
| Meng et al., 2024 China | To investigate the effectiveness of multidomain interventions based on a life-course model of modifiable dementia risk factors in at-risk Chinese older adults. | Individual RCT (parallel) | Community-dwelling at-risk adults aged 60+ | N randomized = 96 (I = 48; C = 48) | **Name:** Multidomain Intervention based on a Life-Course Model  **Category:** Multidomain or multicomponent  **Description:** 6-month community-based program: dementia literacy, PA, cognitive training, social activity, and optional modules.  **Intervention duration:** 6 months  **Follow-up:** 6 months |
| Metzner et al., 2023 Germany | To evaluate the effectiveness of the LoChro-Care intervention on physical, psychological, and social health in older adults with chronic diseases. | Individual RCT (parallel) | Older adults aged 65+ with chronic conditions | N randomized = 491 (I = 244; C = 247) | **Name:** LoChro-Care  **Category:** Integrated or person-centred care  **Description:** 12-month individualized care plans and self-management support through local, collaborative, stepped care.  **Intervention duration:** 12 months  **Follow-up:** 18 months |
| Mitchell et al., 2006 United States | To examine the effects of a nutrition education module on herbal and dietary supplement use among limited-resource older adults. | Cluster RCT | Low-income older adults (mean age 77 years) in Congregate Nutrition sites | N randomized = 1,006 (Intervention = 425; Control = 581); N analyzed = 703 (Intervention = 280; Control = 423) | **Name:** Pills, Potions, and Powders  **Category:** Nutritional intervention  **Description:** 5-session nutrition education module at community sites focusing on safe use of dietary supplements and herbal remedies.  **Intervention duration:** 1.25 months  **Follow-up:** 2.25 months |
| Moore-Harrison et al., 2008 United States | To compare the effects of a walking intervention and nutrition education on functional performance in low-socioeconomic older adults. | Individual RCT (parallel) | Low-socioeconomic older adults aged 60+ | N randomized = 26; N analyzed = 24 | **Name:** 16-Week Walking Program  **Category:** Physical activity or exercise  **Description:** 16-week supervised walking sessions focusing on endurance, balance, flexibility, and strength.  **Intervention duration:** 4 months  **Follow-up:** 4 months |
| Morone et al., 2016 United States | To determine the effectiveness of a mind-body program at increasing function and reducing pain in older adults with chronic low back pain. | Individual RCT (parallel) | Community-dwelling older adults aged 65+ with chronic low back pain and functional limitations | N randomized = 282 | **Name:** Mindfulness-Based Stress Reduction Program  **Category:** Mind-body interventions including Tai Chi, mindfulness, or dance  **Description:** 8-week active mindfulness program with monthly booster sessions focusing on pain management, mindfulness, and stress reduction.  **Intervention duration:** 8 weeks (active phase)  **Follow-up:** 6 months |
| Murphy et al., 2008 United States | To examine the effects of activity strategy training on pain and PA in older adults with knee or hip OA. | Pilot/feasibility RCT | Older adults aged 62+ with symptomatic knee or hip OA | N randomized = 54 | **Name:** Activity Strategy Training (AST)  **Category:** Occupational therapy, home modification, or reablement  **Description:** 8 sessions delivered over approximately 7 months of group and individual OT: activity pacing, joint protection, and personalized in-home strategies.  **Intervention duration:** 8 sessions (~7 months)  **Follow-up:** 6 months |
| Ng et al., 2017 Singapore | To investigate the effect of multi-domain lifestyle interventions on reducing depressive symptoms among frail and pre-frail older persons. | Individual RCT (parallel) | Frail and pre-frail community-dwelling older adults aged 65+ | N randomized = 246 | **Name:** Multi-Domain Lifestyle Interventions  **Category:** Multidomain or multicomponent  **Description:** 24-week program combining nutritional supplementation, physical exercise (strength and balance), and cognitive training.  **Intervention duration:** 6 months  **Follow-up:** 12 months |
| Nikolaus & Bach, 2003 Germany | To evaluate the effect of a multidisciplinary home intervention on reducing falls in frail older adults. | Individual RCT (parallel) | Frail community-dwelling older adults (mean age 81.5 years) | N randomized = 360 | **Name:** Home Intervention Team (HIT)  **Category:** Occupational therapy, home modification, or reablement  **Description:** 12-month multidisciplinary home visits to assess environmental hazards and provide technical aids to prevent falls.  **Intervention duration:** 12 months  **Follow-up:** 12 months |
| Oh et al., 2017 South Korea | To determine the effect of integrated health education and elastic band resistance training on physical function, muscle strength, and quality of life in elderly women. | Individual RCT (parallel) | Community-dwelling elderly women aged 70+ at a senior center | N randomized = 38 | **Name:** Healthy Aging and Happy Aging II (HAHA II)  **Category:** Physical activity or exercise  **Description:** 18-week supervised elastic band resistance training followed by self-directed exercises and health education.  **Intervention duration:** 4.5 months  **Follow-up:** 4.5 months |
| Oh et al., 2021 South Korea | To examine the effects of a rural community-based integrated exercise and health education program on mobility function in older adults with knee OA. | Individual RCT (parallel) | Older adults with knee OA in rural areas | N randomized = 60 (I = 40; C = 20); N analyzed = 32 (I = 21; C = 11) | **Name:** Rural Community-Based Integrated Exercise and Health Education Program  **Category:** Physical activity or exercise  **Description:** 5-month self-directed resistance training combined with monthly health education sessions.  **Intervention duration:** 5 months  **Follow-up:** 5 months |
| Pahor et al., 2006 United States | To assess the effect of a comprehensive PA intervention on the SPPB and other physical performance measures. | Multicenter individual RCT (parallel), assessor-blinded | Sedentary older adults aged 70–89 at risk for disability | N randomized = 424 | **Name:** Lifestyle Interventions and Independence for Elders Pilot (LIFE-P)  **Category:** Physical activity or exercise  **Description:** Center-based and home-based walking, strength, flexibility, and balance training.  **Intervention duration:** 12 months  **Follow-up:** 12 months |
| Parial et al., 2023 Philippines | To assess the preliminary efficacy of dual-task Zumba Gold on cognition, quality of life, and mobility in people with MCI. | Pilot/feasibility RCT | People with MCI aged ≥55 years | N randomized = 60 (I = 30; C = 30) | **Name:** Dual-Task Zumba Gold (DTZ)  **Category:** Mind-body interventions including Tai Chi, mindfulness, or dance  **Description:** 12-week, thrice-weekly Zumba Gold sessions incorporating simultaneous cognitive tasks to target executive function, mobility, and quality of life.  **Intervention duration:** 3 months  **Follow-up:** 3 months |
| Park et al., 2011 South Korea | To examine the effectiveness of an integrated health education and exercise program for community-dwelling older adults with hypertension. | Individual RCT (parallel) | Older adults aged ≥65 with hypertension | N randomized = 40 (I = 18; C = 22) | **Name:** HAHA Program  **Category:** Multidomain or multicomponent  **Description:** 12-week health education, individual counseling, and tailored exercise sessions.  **Intervention duration:** 3 months  **Follow-up:** 3 months |
| Piedra et al., 2018 United States | To assess the effect of an attribution-retraining program on walking behavior among older Hispanic/Latino adults. | Individual RCT (parallel) | Older Hispanic/Latino adults aged ≥60 | N randomized = 572 | **Name:** ¡Caminemos!  **Category:** Physical activity or exercise  **Description:** 24-month exercise classes combined with attribution retraining to modify negative aging beliefs.  **Intervention duration:** 24 months  **Follow-up:** 24 months |
| Piette et al., 2023 United States | To evaluate the feasibility and preliminary effects of the SPEAK! program on perceived cognitive change in older adults. | Pilot/feasibility RCT | Neurologically mixed sample of older adults | N randomized = 38 (I = 17; C = 21) | **Name:** SPEAK! (Seniors Promoting English Acquisition and Knowledge)  **Category:** Cognitive training  **Description:** 8-week weekly webcam conversations between community volunteers and English language learners.  **Intervention duration:** 2 months  **Follow-up:** 2 months |
| Quach et al., 2022 Canada | To explore the impact of a 2-year PA intervention on frailty trajectory and MMD among older adults. | Individual RCT (parallel) | Community-dwelling older adults aged 70–89 | N randomized = 1,635 | **Name:** PA Program  **Category:** Physical activity or exercise  **Description:** 2-year center-based and home-based walking, strength, flexibility, and balance training.  **Intervention duration:** 24 months  **Follow-up:** 24 months |
| Reed et al., 2018 Australia | To determine whether a clinician-led chronic disease self-management support program improves self-rated health in older Australians with multiple chronic conditions. | Individual RCT (parallel) | Older adults aged 60+ with at least two chronic conditions in Adelaide, Australia | N randomized = 254 | **Name:** Chronic Disease Self-Management Support (CDSMS) Program  **Category:** Health education or self-management  **Description:** 6-month clinician-led home visits and telephone consultations with individualized care plans.  **Intervention duration:** 6 months  **Follow-up:** 6 months |
| Reid et al., 2019 United States | To evaluate the safety, feasibility, and preliminary effectiveness of translating the LIFE PA intervention to a real-world community-based setting. | Pilot/feasibility RCT | Community-dwelling older adults aged 65–89 with severe lower extremity functional limitations | N randomized = 40 | **Name:** ENGAGE Program  **Category:** Physical activity or exercise  **Description:** 24-week group-based multimodal PA program at a senior center: aerobic walking, strength, flexibility, and balance.  **Intervention duration:** 6 months  **Follow-up:** 6 months |
| Rejeski et al., 2017 United States | To evaluate the effectiveness of community-based weight loss interventions with and without PA on mobility and strength in older overweight/obese adults. | Individual RCT (parallel) | Older adults aged 60–79 with CVD or metabolic syndrome, overweight or obese | N randomized = 249 | **Name:** Weight Loss (WL) / WL + Aerobic Training / WL + Resistance Training  **Category:** Physical activity or exercise  **Description:** 18-month YMCA group sessions combining dietary advice with aerobic or resistance training.  **Intervention duration:** 18 months  **Follow-up:** 18 months |
| Rubenstein et al., 1994 United States | To assess the effects of home-based comprehensive geriatric assessment with follow-up, health education, and preventive care on health and functional status. | Individual RCT (parallel) | Community-dwelling elderly aged 75+ | N randomized = 414 (I = 215; C = 199) | **Name:** Home-based Comprehensive Geriatric Assessment (CGA)  **Category:** Integrated or person-centred care  **Description:** 3-year annual home visits by geriatric nurse practitioners providing comprehensive medical and social assessments.  **Intervention duration:** 36 months  **Follow-up:** 36 months |
| Shake et al., 2018 United States | To evaluate the efficacy of Bingocize, a game-centered mobile app, in improving physical and cognitive performance in older adults. | Cluster RCT | Community-dwelling older adults (mean age 73 years) | N randomized = 105 (I = 60; C = 45) | **Name:** Bingocize  **Category:** Multidomain or multicomponent  **Description:** 10-week cardiovascular, strength, and flexibility exercises with health education delivered through a bingo game format to promote physical and cognitive engagement.  **Intervention duration:** 2.5 months  **Follow-up:** 2.5 months |
| Sheffield et al., 2012 United States | To evaluate an agency-based OT intervention to facilitate aging in place among at-risk older adults. | Individual RCT (parallel) | Community-dwelling older adults with significant ADL impairments | N randomized = 60 (I = 31; C = 29) | **Name:** Aging in Place  **Category:** Occupational therapy, home modification, or reablement  **Description:** 9-hour restorative OT: home assessments, assistive devices, and home modifications to improve safety and independence.  **Intervention duration:** Not reported  **Follow-up:** 3 months |
| Shumway-Cook et al., 2007 United States | To evaluate the effectiveness of a 12-month community-based intervention on falls and fall-risk factors in community-living older adults. | Individual RCT (parallel) | Community-dwelling older adults aged 65+ | N randomized = 453 | **Name:** Community-Based Multifactorial Fall Prevention Program  **Category:** Physical activity or exercise  **Description:** 12-month group exercise classes, fall prevention education, and individual fall risk assessments.  **Intervention duration:** 12 months  **Follow-up:** 12 months |
| Shvedko et al., 2020 United Kingdom | To examine the feasibility of a PA intervention for loneliness in community-dwelling older adults at risk of loneliness. | Pilot/feasibility RCT | Community-dwelling older adults aged 60+, sedentary, at risk of loneliness | N randomized = 25 (I = 12; C = 13) | **Name:** Physical Activity Intervention for Loneliness (PAIL)  **Category:** Physical activity or exercise  **Description:** 12-week once-weekly group walks combined with health education workshops to promote social interaction and reduce loneliness.  **Intervention duration:** 3 months  **Follow-up:** 3 months |
| Smail et al., 2023 United States | To determine whether depressive symptoms moderate the relationship between a PA intervention and incident MMD among sedentary older adults. | Individual RCT (parallel); secondary data analysis | Older adults aged 70–89, sedentary, with or without clinically relevant depressive symptoms | N randomized = 1,545 | **Name:** PA Program  **Category:** Physical activity or exercise  **Description:** Center-based and home-based aerobic, strength, flexibility, and balance training.  **Intervention duration:** 26.4 months  **Follow-up:** 26 months |
| Smith-Ray et al., 2014 United States | To assess the efficacy of a community-based cognitive training program on balance and gait in Black older adults. | Individual RCT (parallel) | Community-dwelling Black older adults aged 65+ with a history of falls | N randomized = 45 | **Name:** Computer-based Cognitive Training Program  **Category:** Cognitive training  **Description:** 10-week classroom-based computer games targeting executive function (EF) and cognitive training.  **Intervention duration:** 2.5 months  **Follow-up:** 2.5 months |
| Song & Yu, 2019 China | To evaluate the effects of a moderate-intensity aerobic exercise program on cognitive function and quality of life in elderly with MCI. | Individual RCT (parallel) | Community-dwelling elderly aged 60+ with MCI | N randomized = 120 | **Name:** Moderate-intensity aerobic exercise program  **Category:** Physical activity or exercise  **Description:** 16-week stepping exercises combined with upper limb movements to improve cognitive function and physical fitness.  **Intervention duration:** 4 months  **Follow-up:** 4 months |
| Song et al., 2024 China and Hong Kong | To investigate the effects of an aerobic dancing program on sleep quality and cognitive function in older adults with MCI and poor sleep. | Individual RCT (parallel) | Community-dwelling adults aged 60+ with MCI and poor sleep | N randomized = 89 | **Name:** Aerobic Dancing Program  **Category:** Physical activity or exercise  **Description:** 16-week group aerobic dancing with warm-up, aerobic exercises, and cool-down targeting fitness, sleep quality, and cognition.  **Intervention duration:** 4 months  **Follow-up:** 4 months |
| Spoorenberg et al., 2018 Netherlands | To evaluate the effects of the Embrace integrated care service on health, well-being, and self-management among community-living older adults. | Individual RCT (parallel) | Community-living older adults aged 75+ | N randomized = 1,456 (Embrace = 747; Usual Care = 709) | **Name:** Embrace  **Category:** Integrated or person-centred care  **Description:** 12-month multidisciplinary elderly care team: health maintenance, proactive care, and self-management support.  **Intervention duration:** 12 months  **Follow-up:** 12 months |
| Stuck et al., 1995 United States | To evaluate the effect of annual in-home comprehensive geriatric assessments on preventing disability and reducing nursing home admissions. | Individual RCT (parallel) | Elderly individuals aged 75+ living at home | N randomized = 414 (I = 215; C = 199) | **Name:** In-Home Comprehensive Geriatric Assessments  **Category:** Integrated or person-centred care  **Description:** 3-year annual in-home assessments by gerontologic nurse practitioners with telephone follow-up contacts.  **Intervention duration:** 36 months  **Follow-up:** 36 months |
| Stuck et al., 2000 Switzerland | To assess the effectiveness of in-home preventive visits with multidimensional geriatric assessments in delaying disability and reducing nursing home admissions. | Individual RCT (parallel) | Community-dwelling elderly aged 75+ | N randomized = 791 | **Name:** In-Home Preventive Visits  **Category:** Integrated or person-centred care  **Description:** 3-year public health nurse multidimensional assessments and follow-up visits to prevent ADL dependence and nursing home admissions.  **Intervention duration:** 36 months  **Follow-up:** 36 months |
| Sugiyama et al., 2015 United States | To assess the impact of a community-based DSME program on mental health-related quality of life in adults with poorly controlled diabetes. | Individual RCT (parallel) | Adults aged 55+ with HbA1c ≥ 8.0% | N randomized = 516 | **Name:** Diabetes Self-Management Empowerment (DSME) Program  **Category:** Health education or self-management  **Description:** 6-week group-based self-management education with goal setting and problem-solving for diabetes care and mental health.  **Intervention duration:** 1 month  **Follow-up:** 6 months |
| Szanton et al., 2011 United States | To determine the effect size and acceptability of a multicomponent behavior and home repair intervention for low-income disabled older adults. | Pilot/feasibility RCT | Low-income older adults with difficulties in ADLs or IADLs | N randomized = 40 | **Name:** CAPABLE  **Category:** Occupational therapy, home modification, or reablement  **Description:** 6-month in-home visits from OTs, nurses, and handyman services addressing functional and environmental needs.  **Intervention duration:** 6 months  **Follow-up:** 6 months |
| Szanton et al., 2014 United States | To evaluate the effectiveness of a multicomponent bio-behavioral-environmental intervention to reduce disability among low-income older adults. | Individual RCT (parallel) | Low-income older adults aged 65+ with self-care disability | N randomized = 300 | **Name:** CAPABLE  **Category:** Occupational therapy, home modification, or reablement  **Description:** 4-month nursing care, OT, and handyman services to improve functional independence and home safety.  **Intervention duration:** 4 months  **Follow-up:** 5 months |
| Taylor et al., 2016 United Kingdom | To assess the effectiveness and cost-effectiveness of the COPERS intervention for chronic musculoskeletal pain. | Individual RCT (parallel) | Adults with chronic musculoskeletal pain | N randomized = 703 | **Name:** COPERS  **Category:** Health education or self-management  **Description:** 3-day group cognitive-behavioral pain management with follow-up: pain education, communication skills, relaxation, and social integration.  **Intervention duration:** Not reported  **Follow-up:** 12 months |
| Uemura et al., 2018 Japan | To investigate the effects of active learning education on health literacy, cognitive and physical function, PA, and dietary habits in older adults. | Individual RCT (parallel) | Individuals aged 65+ in a rural community in Japan | N randomized = 84 | **Name:** Active Learning Program  **Category:** Health education or self-management  **Description:** 24-week weekly 90-minute group sessions on exercise, diet, nutrition, and cognitive activities.  **Intervention duration:** 6 months  **Follow-up:** 6 months |
| Vaz Fragoso et al., 2015 United States | To evaluate the effect of structured PA on sleep–wake behaviors in sedentary elderly adults with mobility limitations. | Individual RCT (parallel) | Community-dwelling persons aged 70–89, sedentary, with mobility limitations | N randomized = 1,635 | **Name:** Structured PA  **Category:** Physical activity or exercise  **Description:** Walking, strength, flexibility, and balance training to improve physical function and prevent mobility disability.  **Intervention duration:** 24 months  **Follow-up:** 24 months |
| Wang et al., 2016 China | To evaluate the effectiveness of an osteoporosis club as a health education and management model for osteoporosis prevention. | Individual RCT (parallel) | Patients with osteoporosis (139 males; 297 females) | N randomized = 436 | **Name:** Osteoporosis Club  **Category:** Health education or self-management  **Description:** Monthly health education lectures, exercise programs, and yearly social events over 4 years to promote bone health and lifestyle change.  **Intervention duration:** 48 months  **Follow-up:** 48 months |
| Wolf et al., 1996 United States | To evaluate the effects of TC and computerized balance training on biomedical, functional, and psychosocial indicators of frailty and falls in older adults. | Individual RCT (parallel) | Community-dwelling individuals aged 70+ | N randomized = 200 | **Name:** TC and Computerized Balance Training  **Category:** Mind-body interventions including Tai Chi, mindfulness, or dance  **Description:** 15-week group TC sessions and individual computerized balance training to improve fall prevention and psychosocial well-being.  **Intervention duration:** 3.75 months  **Follow-up:** 4.3 months |
| Wong et al., 2020 Hong Kong | To evaluate the effectiveness of a community-based health-social partnership program for discharged non-frail older adults. | Individual RCT (parallel) | Discharged community-dwelling non-frail older adults | N randomized = 75 (I = 37; C = 38) | **Name:** Community-based Health-Social Partnership (CHSP) Program  **Category:** Integrated or person-centred care  **Description:** 12-week nurse case manager and social worker home visits and telephone support post-discharge.  **Intervention duration:** 3 months  **Follow-up:** 3 months |
| Wong et al., 2022 Hong Kong | To evaluate the effects of an interactive mHealth program supported by a health-social partnership team on quality of life among community-dwelling older adults. | Individual RCT (parallel) | Community-dwelling older adults aged 60+ with chronic pain, hypertension, or diabetes | N randomized = 221 (C = 76; mHealth = 71; mHealth + Interaction = 74) | **Name:** mHealth with Interactivity (mHealth+I)  **Category:** Health education or self-management  **Description:** 3-month mobile app health monitoring supported by proactive nurse calls and biweekly case conferences.  **Intervention duration:** 3 months  **Follow-up:** 3 months |
| Woo et al., 2024 Taiwan | To improve physical fitness in prefrail older adults through resistance band and TC exercise. | Individual RCT (parallel) | Prefrail older adults in Veterans' housing in Taiwan | N randomized = 34 (I = 16; C = 18) | **Name:** TC with Resistance Band  **Category:** Mind-body interventions including Tai Chi, mindfulness, or dance  **Description:** 12-week TC movements combined with resistance band training to improve muscle strength, balance, and physical fitness.  **Intervention duration:** 3 months  **Follow-up:** 3 months |
| Wu et al., 2019 Taiwan | To investigate whether a self-efficacy–based community intervention could improve self-care behaviors and health outcomes in older adults at cardiometabolic risk. | Individual RCT (parallel) | Adults aged 50+ at cardiometabolic risk | N randomized = 84 (I = 43; C = 41) | **Name:** Self-Efficacy Health Promotion Program  **Category:** Health education or self-management  **Description:** 4-week in-person group sessions on self-efficacy, blood pressure management, and healthy lifestyle behaviors.  **Intervention duration:** 1 month  **Follow-up:** 6 months |
| Xu et al., 2020 China | To assess the impact of an integrated health care intervention on HRQoL and well-being among older adults with hypertension in rural China. | Individual RCT (parallel) | Older adults aged 60+ with hypertension in rural China | N randomized = 789 (I = 395; C = 394) | **Name:** Integrated Health Care Program  **Category:** Integrated or person-centred care  **Description:** 12-month multidisciplinary health education with periodic follow-ups and psychological counseling.  **Intervention duration:** 12 months  **Follow-up:** 12 months |
| Yang et al., 2023 China | To evaluate the effect of a community-based group rehabilitation program on quality of life, depressive symptoms, and swallowing function in stroke patients with dysphagia. | Individual RCT (parallel) | Stroke patients with dysphagia aged 60+ in China | N randomized = 64 (I = 32; C = 32) | **Name:** Community-based Group Rehabilitation Program  **Category:** Multidomain or multicomponent [Note: includes swallowing rehabilitation, health education, and biofeedback]  **Description:** 8-week swallowing training, health education, game-based biofeedback, and individualized feeding training.  **Intervention duration:** 2 months  **Follow-up:** 2 months |
| Zhao et al., 2023 China | To evaluate the efficacy of the P-bM-tACT program on executive function in older adults with MCI. | Individual RCT (parallel) | Community-dwelling older adults aged 60+ with MCI | N randomized = 92 (I = 46; C = 46) | **Name:** P-bM-tACT  **Category:** Cognitive training  **Description:** 10-week Montessori wooden toy cognitive training targeting EF improvement.  **Intervention duration:** 2.5 months  **Follow-up:** 3 months |

**Abbreviations:** ADL, activities of daily living; IADL, instrumental activities of daily living; HRQoL, health-related quality of life; MCI, mild cognitive impairment; MMD, major mobility disability; OT, occupational therapy; PA, physical activity; RCT, randomized controlled trial; SPPB, Short Physical Performance Battery; TC, Tai Chi.

**LIFE trial note:** Bann et al. (2016), Brown et al. (2020), Fielding et al. (2017), Groessl et al. (2016), Quach et al. (2022), Smail et al. (2023), and Vaz Fragoso et al. (2015) derive from the Lifestyle Interventions and Independence for Elders (LIFE) randomized controlled trial cohort and were included as separate reports because they examined distinct primary outcomes. Differences in reported intervention and follow-up duration reflect outcome-specific definitions and assessment windows across secondary analyses of the LIFE trial cohort.

Study design terminology was standardized across trials. Masking is specified where reported.
