# Supplementary material for: Community-based interventions to support aging in place and functional independence in older adults: a systematic review of randomized controlled trials
Source: Front Public Health. 2026 May 15;14:1828271. doi: 10.3389/fpubh.2026.1828271 (PMC13219341; doi:10.3389/fpubh.2026.1828271)
Supplement: Supplementary file 2 [file Table_2.DOCX]

**Supplementary Table 2. Characteristics of Included Community-Based Interventions and Reported Outcomes**

*Total included studies: 91 publications representing 85 independent randomized controlled trials.*

| **Author(s), Year, Country** | **Delivery Format** | **Key Components** | **Cultural Adaptations** | **Comparison Group** | **Reported Outcomes** |
| --- | --- | --- | --- | --- | --- |
| **Acton et al., 2016, United Kingdom** | Home visits by trained visual rehabilitation officers | Assessment of individual needs; training in use of low vision aids; provision of non-optical aids; mobility training | Tailored to individual needs | Wait-list control with low-vision optometric assessments | Improved visual function (P = 0.031; effect size = 0.55). No improvement in secondary outcomes. |
| **Arai et al., 2007, Japan** | Group sessions in institutional gymnastic rooms and halls led by interdisciplinary team | Progressive resistance training; balance training; functional training; warm-up and cool-down | Adapted to Japanese older adults' physical abilities | Health education (successful aging, cognitive function, fall prevention) | Significant improvements in physical function in participants with lower baseline falls self-efficacy. No significant overall improvement in falls self-efficacy (FES). |
| **Bae et al., 2019, Japan** | Group sessions twice weekly at community resource locations | Physical activities (walking, Tai Chi); cognitive activities (games, arts and crafts); social activities (group events) | Activities tailored to participant preferences and community cultural context | Health education (oral care and nutrition) | Improved spatial working memory. Maintained physical activity levels. No significant impact on other cognitive domains. |
| **Bann et al., 2016, United States** | Supervised sessions at clinical centers and home-based activities; free transportation provided | Walking; strength training; balance; flexibility | Intervention intensity tailored to individual capability | Health education workshops (non-physical activity topics) | Reduced incidence of major mobility disability (MMD). Larger effect sizes observed in participants with higher education levels. |
| **Brown et al., 2020, United States** | Center-based sessions combined with home-based activities; frequent visits and assistance provided | Walking; strength training; flexibility and balance exercises | Not reported | Health education workshops (non-physical activity topics) | Significant reduction in risk of MMD in participants with lower baseline SPPB scores. |
| **Chao et al., 2012, China** | Monthly sessions delivered by trained community health service staff | Health record establishment; health evaluation; tailored exercise programs; health self-management education; telephone consultations; health lectures | Not reported | Usual care without additional health management | Improved health indices and reduced outpatient visits. No significant effect on hospital admissions. |
| **Chen et al., 2021, Taiwan** | Group sessions twice weekly in community-based settings | Sun-style Tai Chi; warm-up; main program; cool-down | Tailored to older adults with knee osteoarthritis | Health education only | Significant improvements in balance, strength, and flexibility in older adults with knee osteoarthritis compared with controls. |
| **Clark et al., 1997, United States** | Group sessions (2 hours/week) and individual sessions (9 hours total) in government-subsidized apartment complexes | Education on health-promoting activities; safety; exercise; adaptive strategies | Adapted for Mandarin-speaking participants | Social activity group; no-treatment control | Significant improvements in quality of life, physical functioning, and mental health. |
| **Clark et al., 2002, United States** | Stage-tailored manuals, newsletters, expert system assessments, and telephone coaching in East Providence community | TTM-based individualized feedback; exercise promotion; nutrition education | Adapted for multicultural population (Portuguese, Cape Verdean, Hispanic) | Single-behavior interventions; control group | Combined intervention group showed superior exercise and nutrition outcomes compared with single-behavior interventions. |
| **Ćwirlej-Sozańska et al., 2018, Poland** | Group sessions at community facility led by physiotherapists | Endurance; balance; resistance; functional exercise; stretching; health education on physical activity, nutrition, and oral care | Not reported | Health education only | Significant improvements in functional fitness, balance, and mobility compared with control group. |
| **Ekelund & Eklund, 2015, Sweden** | Multi-professional team with case manager; home-based care planning and follow-up | Frailty screening at emergency department; brief geriatric assessment; care planning at home; ongoing case manager follow-up | Not reported | Usual care | Slowed decline in self-determination in home activities and social relationships compared with control. |
| **Eklund et al., 2008, Sweden** | Group sessions led by occupational therapists; group discussions and strategy learning | Eight occupational themes including self-care, mobility, and communication | Not reported | Individual program with standard low-vision aids | Health-promotion group maintained ADL levels and reported fewer health problems compared with individual program group. |
| **Endevelt et al., 2011, Israel** | Individual sessions with a dietitian; individualized dietary counseling | Tailored nutritional treatment; education on food supplements; dietary intake evaluation; dietary quality recommendations | Not reported | Medical treatment with standard care booklet; untreated nutrition group | Improved cognitive function, depression scores, and dietary intake. Reduced healthcare costs. |
| **Estebsari et al., 2018, Iran** | Group-based educational sessions (20 sessions of 45-60 minutes each) | Self-efficacy improvement; social support; health-promoting behaviors; physical activity; mental health; elder abuse prevention | Tailored educational materials including CDs, booklets, and pamphlets | Usual care | Significant improvements in elder abuse knowledge, self-efficacy, and social support. Reduced risk of elder abuse. |
| **Evans et al., 2021, United Kingdom** | Face-to-face assessments with telephone or in-person follow-up by general practitioners and community nurses | Multidimensional assessment; regular review; multidisciplinary team management; advance care planning | Not reported | Usual care from primary and community healthcare | Reduced symptom distress with a medium effect size. Lower costs compared with usual care. |
| **Feng et al., 2020, Singapore** | Weekly group choral sessions in community settings | Musical training; social interaction; cognitive exercises through singing; performance events; vocal production techniques | Not reported | Health education program | Improved cognitive function (CCTS) compared with health education. No differences in brain MRI markers. |
| **Fielding et al., 2017, United States** | Supervised sessions at community centers; home-based activity goals | Walking; lower extremity resistance training; balance exercises; stretching | Not reported | Health education program | Dose-dependent increase in physical activity associated with improved physical function and reduced onset of MMD. |
| **Giné-Garriga et al., 2013, Spain** | Twice-weekly sessions at indoor primary care facility; visits to community resources to encourage continued activity | Balance activities; lower-body strength exercises; functional tasks; individualized progression | Not reported | Health education meetings | Reduced fear of falling and improved self-reported health. Effects sustained at 36 weeks. |
| **Gitlin et al., 2006, United States** | Occupational and physical therapy home visits and telephone contacts | Education and problem-solving; home modifications; energy-conserving techniques; balance and muscle strengthening; fall-recovery techniques | Not reported | No-treatment control | Reduced functional difficulties, enhanced self-efficacy, reduced fear of falling, and fewer home hazards. Benefits sustained at 12 months. |
| **González-Guerrero et al., 2014, Spain** | In-person follow-up and telephone contact by multidisciplinary team | Health education; therapeutic control; follow-up assessments; personalized care plan; telephone support | Not reported | Standard healthcare (no structured follow-up) | Reduced hospital readmissions by 30%. Improved quality of life. Extended event-free survival. |
| **Groessl et al., 2016, United States** | Center-based sessions twice weekly supplemented by home-based activities | Walking; strength; flexibility; balance training; behavioral monitoring | Not reported | Health education (successful aging topics, no structured physical activity) | Physical activity intervention associated with lower incidence of mobility disability and higher QALYs compared with health education. Higher intervention cost than health education. |
| **Guerrero et al., 2020, United States** | Weekly in-person classes at local senior centers | Education on sleep, exercise, nutrition, medication management, social engagement | Not reported | Wait-list control | As-treated analysis showed improvements in mental health and social activity satisfaction. No significant effects in intention-to-treat analyses. |
| **Hernandez et al., 2019, United States** | Group-based classes at community senior centers | Strength training; endurance; balance; flexibility; attribution retraining to counter negative aging beliefs | Adapted for older Hispanic/Latino adults; bilingual materials | Generic health education with exercise | Both groups showed reductions in depressive symptoms over 24 months. No significant differences between groups. |
| **Janevic et al., 2022, United States** | Weekly telephone sessions with community health workers; web-based videos; participant workbook; wearable activity trackers | Pain self-management skills; positive psychology activities (Life Review, Savoring, Gratitude Jar, Music as Medicine); goal setting; physical activity encouragement | Culturally relevant examples and language; race-concordant video presenters; activities tailored to local context | Usual care | Significant improvements in pain interference, self-efficacy, and global functioning. |
| **Johnson et al., 2018, Canada** | Home-based sessions delivered by physiotherapists and dietitians with home support worker involvement | Progressive home exercises (10 exercises); nutritional supplementation (Ensure, 2 cans/day) | Exercises adapted to home settings; supplement gluten- and lactose-free | No-treatment control | Exercise improved functional mobility and psychological well-being. Combined exercise and nutrition improved mobility and strength. |
| **Jones et al., 2019, Canada** | Group sessions at YMCA in partnership with YMCA Okanagan | Strength and resistance exercise; hearing education; communication strategies; psychosocial support | Not reported | Group audiological rehabilitation (GAR) only | Significant improvements in functional fitness. Reduced loneliness, with greater reduction in participants attending 80% or more of sessions. |
| **Keall et al., 2017, New Zealand** | Home modifications performed by qualified builders through community program | Installation of handrails, grab rails, slip-resistant surfacing, lighting improvements | Not reported | No home modifications | Reduced injury costs by 33%. High benefit-cost ratio, particularly for older adults. |
| **Khodneva et al., 2021, United States** | Telephone-delivered sessions by trained peer advisors targeting rural communities | CBT principles; diabetes self-management; goal setting; stress management | Culturally adapted for rural African American adults | General health education (no CBT or diabetes-specific content) | Significant improvements in pain self-efficacy, pain intensity, and functional limitations over 12 months. |
| **Kim et al., 2013, Japan** | Twice-weekly group exercise sessions combined with daily heat application | Progressive resistance exercises; balance training; heat therapy using heated suit (HSGS) | Adapted to Japanese elderly women | Health education only | Combination of exercise and thermal therapy reduced pain and improved physical function compared with individual interventions. |
| **Kim et al., 2016, Japan** | Twice-weekly in-person group exercise classes with daily nutritional supplementation | Progressive resistance and aerobic exercise; leucine-enriched amino acid supplementation; tea catechins | Chair exercises adapted for elderly participants | Health education only | Significant improvements in body fat mass, walking speed, and vitamin D levels compared with controls. |
| **King et al., 2007, United States** | Telephone-based intervention (human counselors or automated system) | Individualized physical activity advice; goal-setting; self-monitoring; behavioral strategies | Not reported | Health education control | Both human and automated interventions increased physical activity. Human-delivered advice associated with greater well-being improvements. |
| **King et al., 2017, United States** | Center-based and home-based activities with community outreach recruitment | Aerobic exercise (primarily walking); lower-extremity strengthening; flexibility; balance exercises | Not reported | Health education | Increased exercise and leisure walking. Reduced walking for errands, particularly in less compact neighborhoods. |
| **King et al., 2021, United States** | Group sessions and mobile app data collection with neighborhood advocacy; residents engaged as citizen scientists | Behavioral change strategies (ALED); neighborhood assessments; data-driven advocacy | Materials provided in English and Spanish; adapted for diverse populations | ALED plus health education | Adding the Our Voice intervention increased and sustained physical activity. Promoted neighborhood-level changes. |
| **Kohn et al., 2023, United States** | In-person group sessions in community-based settings (2 sessions/week, 60 minutes each) | Eight prescribed Tai Chi movements | Not reported | Health education program (HAP-E) | Tai Chi group showed smaller declines in mental health and resilience during COVID-19 compared with health education group. |
| **Lamb et al., 2020, United Kingdom** | Exercise sessions by physical therapists; multifactorial interventions by nurses, GPs, and geriatricians through NHS provider network | Home exercises and balance training; comprehensive fall risk assessment; home modifications; medication review | Falls-risk screening adapted for mail administration | Advice by mail only | Small improvements in quality of life. Neither exercise nor multifactorial fall prevention reduced fracture rates. Intervention was cost-saving compared with mail advice. |
| **Lee et al., 2023, Japan** | Weekly sessions at community sports facilities | Dual-task training combining cognitive tasks with physical exercise; social activities | Not reported | Health education (no exercise or cognitive health content) | Modest improvements in cognitive and physical function. Increased step count and moderate-to-vigorous physical activity. |
| **Liang et al., 2021, Taiwan** | Group sessions and health education at community-based sites | Physical fitness activities (strength, balance, flexibility); cognitive training (reasoning, memory); nutritional advice; chronic disease prevention education | Not reported | Conventional health education via telephone | Improved cognitive performance, handgrip strength, and frailty indicators. Particularly beneficial in participants with physio-cognitive decline syndrome. |
| **Liao et al., 2018, China** | Group therapy sessions in community settings | Tai Chi movements accompanied by relaxing Chinese folk music | Tai Chi and music tailored to cultural preferences | Routine health education | Significant reduction in depressive symptoms in intervention group compared with control. |
| **Loh et al., 2015, Malaysia** | Group sessions twice weekly with additional home-based exercises in low-cost public housing | Strength; balance; flexibility exercises; nutrition education; oral care; ongoing support | Adapted for urban poor Malaysian settings | General health education (no physical activity or lifestyle counseling) | Significant improvements in physical performance and quality of life. Reduced disability risk. |
| **Lu et al., 2015, China** | Monthly interactive workshops with visual aids in community settings for hypertensive patients | Hypertension knowledge; lifestyle changes; medication adherence; culturally relevant visual aids (cartoons, animations) | Simplified language and culturally relevant educational tools | Self-learning reading; regular lectures | Interactive education workshops most effective in improving blood pressure control, hypertension knowledge, and lifestyle changes compared with other formats. |
| **Marconcin et al., 2022, Portugal** | Group sessions (90 minutes) combining self-management and exercise in community settings | Self-management principles; symptom management; exercise; communication skills; healthy eating; medication management; physical fitness | Not reported | Educational programme only | Significant improvements in self-efficacy, physical activity, and balance. No significant changes in health-related quality of life. |
| **Markle-Reid et al., 2006, Canada** | Home visits and telephone contacts by Registered Nurse | Health assessment; health education; coordination of community services; empowerment strategies | Not reported | Usual home care services | Improved mental health, reduced depression, and enhanced social support. No additional cost compared with usual care. |
| **Marquez et al., 2014, United States** | Twice-weekly dance sessions and bi-monthly dance parties at community senior center | Latin dance styles (Merengue, Cha Cha Cha, Bachata, Salsa); social cognitive framework discussion sessions | Delivered in Spanish; tailored for older Latino adults; peer support model | Health education control with Spanish-language materials | Significant improvements in physical activity, self-efficacy, physical and cognitive function, and disability scores. |
| **Marquez et al., 2017, United States** | In-person dance classes at community senior center with Latino population involvement | Latin dance styles (Merengue, Cha Cha Cha, Bachata, Salsa) | Bilingual instructors and materials; culturally appropriate for Latino community | Health education program | Dance group showed greater improvement in episodic memory compared with health education. Both groups improved in global cognition. |
| **Martin-Valero et al., 2013, Spain** | Sessions by health specialist at Sports and Physical Medical Center; recruited from Primary Healthcare Centers | Structured exercise sessions (warm-up, aerobic phase, cooling-stretch) tailored to individual capacity | Not reported | Health education and routine activities | Significant improvement in quality of life (EQ-5D) in male participants. No significant changes in cardiopulmonary function. |
| **Meng et al., 2024, China** | Online (WeChat) and face-to-face activities with community worker engagement and one-on-one volunteer services | Dementia literacy; physical activity; cognitive training; social activity; optional modules (nutrition, COVID-19 prevention, tobacco cessation, depression, diabetes management) | Adapted to Chinese older adults and local community context | Health education | Reduced dementia risk and improved cognitive function. Reduced loneliness. Improved dementia literacy. |
| **Metzner et al., 2023, Germany** | In-person and telephone sessions with chronic care managers integrated with local healthcare providers | Comprehensive assessment; individualized care plans; self-management support; modules for mild depression and diabetes; care plan monitoring | Not reported | Usual care | No significant effect on primary or secondary outcomes. Both groups showed decline in functional health over follow-up. |
| **Mitchell et al., 2006, United States** | Sessions by Cooperative Extension agents at Congregate Nutrition sites for low-income older adults | Education on herbal and dietary supplement use; risks; self-efficacy building; personal action plan | Not reported | Health education on weight management and exercise | Increased multivitamin use, label reading, and carrying medication lists. No significant improvement in calcium use. |
| **Moore-Harrison et al., 2008, United States** | Supervised walking classes (3 times/week) at public housing complex | Endurance; balance; flexibility; strength through walking and exercises | Not reported | Nutrition education (fruits and vegetables) | Significant improvements in peak aerobic capacity (18.9%) and physical function (25%) compared with controls. |
| **Morone et al., 2016, United States** | Group sessions with in-person facilitation | Mindfulness techniques: body scan, sitting practice, walking meditation, mindful stretching | Not reported | Health education program (10 Keys to Healthy Aging) | Short-term improvements in function and pain. Sustained pain reduction for current and severe pain. No sustained improvement in functional outcomes. |
| **Murphy et al., 2008, United States** | Group sessions and individualized home visits at senior housing facilities and senior center | Activity strategy training (AST): activity pacing, joint protection, body mechanics; personalized in-home strategies | Not reported | Exercise plus health education (pain, exercise, diet, medication management) | AST group showed significantly higher peak physical activity at post-test. Trends toward improved pain and physical function. |
| **Ng et al., 2017, Singapore** | Group sessions and home-based exercises for community-dwelling older adults | Nutritional supplementation; physical exercise (strength and balance); cognitive training (memory, attention, reasoning) | Not reported | Usual care with access to standard services | Combination intervention significantly reduced depressive symptoms at 6 and 12 months. Nutritional intervention alone also showed effects at 12 months. |
| **Nikolaus & Bach, 2003, Germany** | Home visits by multidisciplinary team with family involvement | Assessment of home for environmental hazards; advice on modifications; provision of technical and mobility aids | Not reported | Comprehensive geriatric assessment with usual home care | Reduced falls by 31%, particularly in participants with a history of recurrent falls. |
| **Oh et al., 2017, South Korea** | Supervised sessions followed by self-directed home-based exercises at senior center | Health education; elastic band resistance training (supervised and self-directed); progressive intensity; behavioral change counseling | Not reported | Low-intensity stretching program | Significant improvements in SPPB, leg strength, and muscle quality compared with controls. |
| **Oh et al., 2021, South Korea** | Self-directed home-based resistance training with monthly supervised visits and weekly telephone follow-up | Monthly health education; self-directed resistance training with guidance; exercise logbook; telephone follow-up | Not reported | Health education only | Significant improvements in mobility function. Maintained pain and stiffness levels. |
| **Pahor et al., 2006, United States** | Center-based sessions transitioning to home-based exercises; community center and counseling session engagement | Walking; lower extremity strength training; balance exercises; flexibility; group-based behavioral counseling | Not reported | Health education program (successful aging topics, minimal physical activity) | Significant improvements in physical performance (SPPB, 400-meter walk speed). Reduced incidence of MMD. |
| **Parial et al., 2023, Philippines** | In-person sessions at community center with community leader and local physician involvement | Dual-task Zumba Gold: integration of cognitive tasks with physical exercises | Adapted popular English and Filipino songs for participant engagement | Health education (lifestyle modification and self-reported physical activities) | Significant improvements in cognition, executive function, memory, quality of life, and mobility. Moderate to large effect sizes reported. |
| **Park et al., 2011, South Korea** | Group health education, individual counseling, and exercise sessions at senior center | Weekly health education; mid-program counseling; bi-weekly elastic band exercise sessions | Exercise tailored based on individual fitness level | Usual care | Significant reduction in systolic blood pressure. Improved exercise self-efficacy and quality of life. |
| **Piedra et al., 2018, United States** | Weekly group sessions transitioning to monthly/bi-monthly sessions at community senior centers | Attribution retraining; exercise classes; fotonovela educational tool | Bilingual health educators; culturally tailored materials including fotonovela | Generic health education with exercise classes | Increased walking behavior at 12 months. No sustained effect at 24 months. |
| **Piette et al., 2023, United States** | Webcam-based conversations between community volunteers and English language learners | Semi-structured conversations based on Cognitive Stimulation Therapy principles | Pairings designed to address language and cultural diversity | Wait-list control | Improved perceived cognitive change, particularly in participants with better baseline cognition. |
| **Quach et al., 2022, Canada** | Center-based and home-based sessions for community-dwelling older adults | Walking; lower extremity strength training; flexibility exercises; balance training | Not reported | Health education program | Physical activity reduced MMD and mortality in frailer participants. Similar frailty trajectories across both groups. |
| **Reed et al., 2018, Australia** | Clinician-led home visits and telephone calls | Goal setting; individualized care plans; problem-solving; self-management assessment tools (Partners in Health scale, Cue and Response interview, Problems and Goals assessment) | Not reported | Attention-control (health education and positive clinician attention without structured self-management support) | Participants reported greater self-rated health improvements. No significant differences in secondary outcomes. |
| **Reid et al., 2019, United States** | Group-based sessions at senior center in partnership with local Council on Aging | Multimodal physical activity: moderate-intensity aerobic walking, strength training, flexibility, balance; safety and adherence monitoring | Not reported | Health education with biweekly sessions and light stretching | Significant improvements in SPPB and executive function. Trends toward improved quality of life and reduced falls. |
| **Rejeski et al., 2017, United States** | Group sessions at YMCA supervised by YMCA staff trained by researchers | Weight loss sessions (diet: 20-25% proteins, 25-30% fats, 45-55% carbohydrates); aerobic training (walking); resistance training | Not reported | Weight loss alone; weight loss with aerobic training; weight loss with resistance training | Weight loss plus aerobic training and weight loss plus resistance training improved 400-meter walk time more than weight loss alone. Both preserved knee strength; weight loss alone led to decline. |
| **Rubenstein et al., 1994, United States** | In-home visits by geriatric nurse practitioners with quarterly follow-up and telephone contacts | Comprehensive medical and social history; routine physical exams; screening tests; home safety evaluations; personalized recommendations | Tailored to individual needs | Usual care | Better compliance with health recommendations in the intervention group. Full outcome data pending final analysis. |
| **Shake et al., 2018, United States** | Mobile app sessions on tablets led by trained staff at senior centers | Cardiovascular, strength, and flexibility exercises; health education on osteoarthritis and fall risks; Bingocize game | Not reported | Health education plus bingo game (no exercise) | High adherence (>93%). Significant improvements in upper and lower body strength and cognitive performance. |
| **Sheffield et al., 2013, United States** | In-home assessment, provision of assistive devices, and home modifications | Adaptive equipment; home safety improvements; training in medication management | Not reported | Usual care (case management, periodic contacts, possible in-home aide services) | Improved home safety, quality of life, and reduced fear of falling. No significant improvement in functional status or fall reduction. |
| **Shumway-Cook et al., 2007, United States** | Exercise classes (3 times/week) and education sessions (6 hours total) at community centers, senior facilities, and recreation programs | Exercise classes; fall prevention education; fall risk assessment feedback to healthcare providers | Not reported | Written materials on fall prevention | Significant improvements in balance, leg strength, and mobility. Reduction in falls (25%) was not statistically significant in intention-to-treat analysis. |
| **Shvedko et al., 2020, United Kingdom** | Once-weekly group walks and workshops; social support built through group discussions | Group walks; health education; social interaction | Not reported | Wait-list control | Moderate improvements in psychosocial outcomes. Recruitment and adherence challenges identified. |
| **Smail et al., 2023, United States** | Center-based sessions and home exercises tailored to participant ability | Aerobic exercise (>150 min/week); strength, flexibility, and balance training | Not reported | Health education (non-physical activity topics) | Reduced risk of MMD. No difference in outcomes between participants with or without depressive symptoms. |
| **Smith-Ray et al., 2014, United States** | Classroom sessions at community senior centers | Cognitive training targeting executive function using computerized games (Road Tour, Jewel Diver, Sweep Seeker) | Not reported | Measurement-only control | Significant improvements in balance (BBS) and gait speed. No significant improvement in distracted gait speed. |
| **Song & Yu, 2019, China** | Group-based sessions at community healthcare centers | Warm-up; stepping exercises (moderate intensity per Borg scale); upper limb movements; cool-down; adherence-enhancing motivational strategies | Culturally relevant movements incorporated | Health education program (16 weeks, bi-weekly sessions on general health topics) | Significant improvements in cognitive function, quality of life, depressive symptoms, and sleep quality. |
| **Song et al., 2024, China and Hong Kong** | Group-based sessions with community engagement | 60-minute sessions: warm-up, aerobic dancing, cool-down | Not reported | Health education sessions | Significant improvements in sleep quality (PSQI) and cognitive function (MoCA). |
| **Spoorenberg et al., 2018, Netherlands** | Multidisciplinary Elderly Care Team with self-management support; regular case manager contact and community meetings | Individual care plans; proactive and preventive care; health maintenance education | Not reported | Usual care | Marginal improvement in self-management knowledge. Clinically relevant deterioration in ADL. No significant differences in well-being. |
| **Stuck et al., 1995, United States** | In-home visits by gerontologic nurse practitioners with 3-monthly follow-up and telephone contact | Annual comprehensive assessments; personalized recommendations; monitoring; health education | Not reported | Usual medical care | Significantly delayed disability. Reduced permanent nursing home admissions. |
| **Stuck et al., 2000, Switzerland** | In-home visits by public health nurses with regular follow-up and geriatrician consultation | Comprehensive geriatric assessment; follow-up; health education; problem identification; adherence facilitation | Not reported | Usual care (no preventive visits) | Reduced ADL dependence and nursing home admissions in low-risk elderly. No benefit observed in high-risk elderly. |
| **Sugiyama et al., 2015, United States** | In-person group sessions facilitated by health educators; self-management challenges and personal experiments | Empowerment model; personalized self-care plans; problem-solving; group discussions | Conducted in English and Spanish with culturally relevant materials | Six weekly lectures on unrelated geriatric topics | Improved mental health-related quality of life (HRQoL) independent of glycemic control, social support, or perceived empowerment. |
| **Szanton et al., 2011, United States** | In-home visits by occupational therapists, nurses, and handyman services | Occupational therapy for ADL/IADL support; nursing care for health management; home safety modifications | Tailored to needs of low-income, predominantly African American participants | Attention-control (sedentary activities of choice) | Significant improvements in ADL/IADL, quality of life, and falls efficacy. Effect sizes ranged from 0.55 to 0.89. |
| **Szanton et al., 2014, United States** | In-home visits by nurses, occupational therapists, and handymen with individualized goal-setting | Home safety modifications; ADL/IADL support; pain and depression management; medication review; fall prevention; patient activation | Tailored to cultural and individual needs of low-income older adults | Attention-control (sedentary activities including reminiscence therapy and card games) | Significant improvements in ADL/IADL, SPPB, quality of life, and home safety. Potential healthcare cost savings reported. |
| **Taylor et al., 2016, United Kingdom** | Facilitated group sessions in community settings | Cognitive behavioral approaches; self-efficacy improvement; pain education; communication skills; relaxation techniques; social integration activities | Not reported | Usual care plus relaxation CD | Significant reduction in pain-related disability. Significant improvements in depression and social integration at 12 months. Lower costs than usual care. |
| **Uemura et al., 2018, Japan** | Weekly 90-minute sessions led by licensed physical therapists and physical education teachers in rural community settings | Exploratory learning; group work; self-planning for behavioral change; health promotion covering exercise, diet, nutrition, and cognitive activity | Not reported | No-treatment control | Significant improvements in health literacy, memory, gait speed, balance, physical activity, and dietary variety. No adverse events reported. |
| **Vaz Fragoso et al., 2015, United States** | Center-based sessions and home-based activities | Walking; strength training (ankle weights); balance training; flexibility exercises | Not reported | Health education (weekly workshops for first 26 weeks, then monthly) | Physical activity reduced likelihood of poor sleep quality (PSQI >5) by 20%. No significant effects on ISI or ESS scores. |
| **Wang et al., 2016, China** | Monthly health education lectures and exercise programs with yearly communication events; integrated specialist team | Systematic health education; exercise programs; communication events; digital database management | Adapted for Chinese population | Self-management group with no organized education or exercise | Significant improvements in osteoporosis knowledge, health beliefs, compliance, quality of life, pain, and bone mineral density (BMD). |
| **Wolf et al., 1996, United States** | Group Tai Chi sessions and individual computerized balance training; Tai Chi groups continued informally post-study | Tai Chi: slow rhythmic movements, reduced base of support, body rotation, reciprocal arm movements; computerized balance feedback training | Not reported | Education group (weekly meetings with gerontological nurse) | Tai Chi reduced risk of multiple falls by 47.5%. Improved grip strength, reduced systolic blood pressure, and improved psychosocial well-being. |
| **Wong et al., 2020, Hong Kong** | Home visits and telephone calls by nurse case managers and social workers | Comprehensive assessment; health education; goal empowerment; care coordination | Not reported | Usual discharge care with monthly social call | Significant improvements in mental HRQoL, ADL, and reduction in depressive symptoms. |
| **Wong et al., 2022, Hong Kong** | Mobile app with proactive nurse calls and biweekly case conferences; health-social partnership integrating nurses, social workers, and GPs | Self-management support; health monitoring; individualized education; case management | Not reported | No mHealth app or health-social care services; mHealth app only | Improved self-efficacy, systolic blood pressure, pain levels, and health service utilization in mHealth plus integrated care group. No significant improvement in quality of life. |
| **Woo et al., 2024, Taiwan** | Instructor-led group sessions at Veterans' housing | Resistance band exercises targeting specific muscle groups; Tai Chi for flexibility and stability | Not reported | Health education on frailty | Significant improvements in frailty index, physical fitness, IADL scores, quality of life, and heart rate variability (HRV). |
| **Wu et al., 2019, Taiwan** | In-person group sessions with follow-up during community health screenings | Health promotion skills; self-care activities; blood pressure management; diet and exercise guidance; psychological support | Not reported | Conventional health education | Significant improvements in BMI, blood pressure, HDL-C, and self-efficacy compared with controls. |
| **Xu et al., 2020, China** | Multidisciplinary team sessions providing health education and periodic follow-up at county hospitals, township health centers, and village clinics | Health education; periodic follow-ups; annual physical exams; psychological counseling | Not reported | Standard care | Significant improvements in health-related quality of life (HRQoL). Reduced anxiety and depression. Improved health knowledge. |
| **Yang et al., 2023, China** | Group training sessions with speech-language pathologists (5 days/week, 60 minutes/day) in community public spaces; family members and caregivers included | Swallowing function training; health education; game-based biofeedback; experience sharing; individualized feeding training | Not reported | Standard care (individual rehabilitation training and educational handbooks) | Significant improvements in swallowing function, depressive symptoms, and quality of life. |
| **Zhao et al., 2023, China** | Small group sessions (maximum 6 participants) supervised by trained community nurses at community healthcare centers | Adaptive cognitive training using Montessori wooden toys targeting executive function | Tasks adapted to individual cognitive performance level | Wait-list control with health education on MCI | Significant improvements in executive function, working memory, and psychomotor speed. Benefits sustained at 3-month follow-up. |

**Abbreviations:** ADL = Activities of Daily Living; BBS = Berg Balance Scale; BMD = Bone Mineral Density; BMI = Body Mass Index; CBT = Cognitive Behavioral Therapy; CCTS = Cognitive Change and Treatment Scale; EQ-5D = EuroQol Five Dimension Questionnaire; FES = Falls Efficacy Scale; GP = General Practitioner; HDL-C = High-Density Lipoprotein Cholesterol; HRQoL = Health-Related Quality of Life; HRV = Heart Rate Variability; IADL = Instrumental Activities of Daily Living; ISI = Insomnia Severity Index; MCI = Mild Cognitive Impairment; MMD = Major Mobility Disability; MoCA = Montreal Cognitive Assessment; OA = Osteoarthritis; PSQI = Pittsburgh Sleep Quality Index; QALYs = Quality-Adjusted Life Years; SPPB = Short Physical Performance Battery; TTM = Transtheoretical Model.

**Note:** Entries are ordered alphabetically by first author surname. Where cultural adaptations were not described in the original publication, this is indicated as 'Not reported.' Findings are presented as reported by study authors without interpretive judgment.
