# Supplementary material for: Community-based interventions to support aging in place and functional independence in older adults: a systematic review of randomized controlled trials
Source: Front Public Health. 2026 May 15;14:1828271. doi: 10.3389/fpubh.2026.1828271 (PMC13219341; doi:10.3389/fpubh.2026.1828271)
Supplement: Supplementary file 3 [file Table_3.DOCX]

**Supplementary Table 3. Outcome Measures of Community-Based Interventions**

*Total included studies: 91 publications representing 85 unique randomized controlled trials (multiple publications reported outcomes from some trials).*

| **Author(s), Year, Country** | **Outcome Domains / Measures** | **Primary or Main Reported Outcomes** | **Additional Reported Outcomes** | **Main Reported Results** | **Independence-Related Outcomes (Direct and Proxy)** |
| --- | --- | --- | --- | --- | --- |
| **Acton et al., 2016, United Kingdom** | Visual function (VA LV VFQ-48); depression (PHQ-9) | Visual function | Depression, loneliness | Improvement in visual function (p = 0.031; effect size = 0.55); no significant change in depression or loneliness | Visual function improved; no direct independence measure reported |
| **Arai et al., 2007, Japan** | Falls self-efficacy (FES); physical function (balance, walking velocity, strength) | Falls self-efficacy (FES) | Walking velocity, lower-extremity strength, balance | FES: no significant change; walking velocity and lower-extremity strength improved (p < 0.05) | Walking velocity and strength improved; FES: no significant change; no direct independence measure reported |
| **Bae et al., 2019, Japan** | Cognitive function (spatial working memory); physical activity; physical function (grip strength, walking speed); depressive symptoms | Spatial working memory | Grip strength, walking speed, depressive symptoms | Significant improvement in spatial working memory (p < 0.05); physical activity levels maintained relative to controls at follow-up (p < 0.05) | Spatial working memory improved; no significant change in ADL/IADL |
| **Bann et al., 2016, United States** | Mobility disability (MMD, persistent mobility disability) | Major mobility disability (MMD) | Persistent mobility disability | Reduced incidence of MMD (p < 0.05); no significant modifying effect of education or income | MMD incidence reduced |
| **Brown et al., 2020, United States** | Physical performance (SPPB); mobility disability (MMD) | Major mobility disability (MMD) | SPPB score change | Secondary analysis of the LIFE trial cohort: decline in SPPB score was associated with increased subsequent risk of MMD (HR = 4.76, p < 0.05) | Secondary analysis of the LIFE trial cohort: SPPB decline was associated with an increased subsequent risk of MMD |
| **Chao et al., 2012, China** | Health knowledge; diet quality; health service utilization | Health knowledge; diet quality | Outpatient visit frequency | Significant improvement in diet quality and health knowledge (p < 0.01); reduced outpatient visits (p < 0.01) | Health knowledge and diet quality improved; outpatient visits reduced |
| **Chen et al., 2021, Taiwan** | Functional fitness (balance, flexibility, endurance, lower-body strength) | Functional fitness | Balance, flexibility, endurance | Significant improvement in functional fitness (p < 0.01) | Functional fitness improved |
| **Clark et al., 1997, United States** | Functional status; life satisfaction; health perception | Functional status | Life satisfaction, health perception | Significant improvements across multiple health domains (p < 0.05 to p < 0.01) | Functional status improved |
| **Clark et al., 2002, United States** | Physical activity levels; dietary behavior; functional ability | Physical activity levels | Dietary behavior, general health outcomes | Significant improvements in physical activity and dietary behavior (p < 0.05) | Physical activity increased; dietary behavior improved |
| **Ćwirlej-Sozańska et al., 2018, Poland** | Mobility (TUG); functional reach (FRT); fitness (SFT); static balance | Functional fitness, mobility | Static balance | Significant improvements in mobility and functional fitness (p < 0.001); static balance: no significant change | Mobility and functional fitness improved (TUG, FRT, SFT); static balance: no significant change |
| **Ekelund & Eklund, 2015, Sweden** | Self-determination in daily activities and social relationships | Self-determination in daily activities | None | Significant improvement in self-determination at home and in social relationships (p < 0.05) | Self-determination in daily activities improved |
| **Eklund et al., 2008, Sweden** | ADL dependence; self-reported health problems | ADL dependence | Self-reported health problems | Health-promotion group maintained ADL levels vs. individual program group (p < 0.05) | ADL levels maintained |
| **Endevelt et al., 2011, Israel** | Cognitive function; depression; nutritional intake; healthcare costs | Cognitive function; depression | Nutritional intake, healthcare costs | Significant improvements in cognitive function and depression scores (p < 0.05) | Cognitive function improved; economic outcome reported; no direct independence measure |
| **Estebsari et al., 2018, Iran** | Elder abuse knowledge; self-efficacy; social support; health-promoting behaviors | Elder abuse knowledge; self-efficacy | Social support, health-promoting behaviors | Significant improvements in knowledge, self-efficacy, and social support (p < 0.001) | Self-efficacy and social support improved |
| **Evans et al., 2021, United Kingdom** | Symptom distress (IPOS); health-related quality of life (EQ-5D); caregiver burden | Symptom distress (IPOS) | Health-related quality of life (EQ-5D), caregiver burden | Reduced symptom distress compared with usual care at 12 weeks (mean difference −1.20; 95% CI −2.37 to −0.027; medium effect size). No significant differences in secondary outcomes (EQ-5D or caregiver burden). Economic analysis indicated cost-effectiveness. | Symptom distress reduced; no direct independence measure reported |
| **Feng et al., 2020, Singapore** | Cognitive function (composite cognitive test score, CCTS); brain MRI markers; biomarkers | Cognitive test score (CCTS) | Brain MRI markers, biomarkers | Significant improvement in cognitive score (p < 0.05); brain MRI markers: no significant change | Cognitive test score (CCTS) improved |
| **Fielding et al., 2017, United States** | Gait speed; physical performance (SPPB); mobility disability (MMD); physical activity | Gait speed; major mobility disability (MMD) | SPPB score, physical activity | Dose-dependent improvements in gait speed and SPPB (p < 0.001); reduced MMD incidence | MMD reduced; gait speed and SPPB improved |
| **Giné-Garriga et al., 2013, Spain** | Fear of falling (ABC scale); health status (SF-12 physical and mental composite) | Fear of falling (ABC scale) | Physical and mental composite scores (SF-12) | Significant reduction in fear of falling and improved health status (p < 0.001) | Fear of falling reduced; SF-12 health status improved |
| **Gitlin et al., 2006, United States** | ADL/IADL difficulty; fear of falling; self-efficacy; home hazards | ADL/IADL difficulty | Fear of falling, home hazards, self-efficacy | Significant reduction in ADL/IADL difficulty (p < 0.05); improved self-efficacy (p < 0.05) | ADL/IADL difficulty reduced; self-efficacy improved |
| **González-Guerrero et al., 2014, Spain** | Event-free survival; functional capacity; quality of life (HRQoL) | Event-free survival | Functional capacity, quality of life | 30% reduction in adverse events; improved quality of life (p < 0.05) | Functional capacity improved; HRQoL improved |
| **Groessl et al., 2016, United States** | Mobility disability (MMD); quality-adjusted life years (QALYs) | Major mobility disability (MMD) | Quality-adjusted life years (QALYs) | Reduction in MMD (5.43%, p < 0.05); significant QALY gains (p < 0.05) | MMD reduced (5.43%); economic outcome reported (QALYs) |
| **Guerrero et al., 2020, United States** | Mental health; physical health; patient activation | Mental health | Physical health, patient activation | Significant improvement in mental health scores (p < 0.01) in as-treated analysis; no significant effect in intention-to-treat analysis | Mental health scores improved (as-treated analysis) |
| **Hernandez et al., 2019, United States** | Depressive symptoms (GDS) | Depressive symptoms (GDS) | None | Significant reduction in depressive symptoms at 24 months (p < 0.001) | No direct independence measure reported |
| **Janevic et al., 2022, United States** | Pain interference; pain self-efficacy; physical function; social participation; resilience | Pain interference | Social participation, resilience, pain self-efficacy | Significant reduction in pain interference (p < 0.05); improvement in pain self-efficacy (p < 0.01) | Pain interference reduced; pain self-efficacy improved |
| **Johnson et al., 2018, Canada** | Functional mobility (Functional Reach, TUG, 6-minute walk test); balance confidence; psychological well-being | Functional mobility | Balance confidence, psychological well-being | Significant improvements in functional mobility and balance confidence (p < 0.05) | Functional mobility and balance confidence improved |
| **Jones et al., 2019, Canada** | Functional fitness (gait speed, Sit-to-Stand); loneliness; hearing-related quality of life; depression | Functional fitness | Hearing-related quality of life, loneliness, depression | Significant improvements in functional fitness and hearing-related quality of life (p < 0.05); loneliness reduced | Functional fitness improved; loneliness reduced |
| **Keall et al., 2017, New Zealand** | Fall-related injury costs; disability-adjusted life years (DALYs); social costs | Injury costs; social costs | Disability-adjusted life years (DALYs) | Significant reduction in fall-related injury costs and DALYs (p < 0.05) | Economic outcome reported; no direct independence measure |
| **Khodneva et al., 2021, United States** | Pain self-efficacy; pain intensity; functional limitations | Pain self-efficacy; pain intensity | Functional limitations | Significant improvements in pain self-efficacy and functional limitations (p < 0.05) | Pain self-efficacy improved; functional limitations reduced |
| **Kim et al., 2013, Japan** | Knee pain (VAS); knee function (JKOM); muscle strength; functional mobility | Knee pain (VAS) | Functional mobility, quality of life | Significant reduction in knee pain (VAS, p < 0.05); significant improvement in functional mobility (p < 0.05) | Knee pain (VAS) reduced; functional mobility improved |
| **Kim et al., 2016, Japan** | Body composition (body fat mass); physical function (walking speed, grip strength); vitamin D levels | Body fat mass; walking speed | Grip strength, vitamin D levels | Significant reductions in body fat mass; increased walking speed (p < 0.05) | Body fat mass reduced; walking speed increased |
| **King et al., 2007, United States** | Physical activity (PAR, CHAMPS); well-being (Vitality Plus Scale); fitness satisfaction | Physical activity levels | Well-being, fitness satisfaction | Significant increases in physical activity and well-being in both groups (p < 0.05) | Physical activity increased; well-being improved |
| **King et al., 2017, United States** | Physical activity (CHAMPS: walking, gardening, yard work) | Exercise and leisure walking | Gardening, yard work | Significant increase in exercise and leisure walking (p < 0.05); gardening activities maintained | Walking minutes increased; gardening activities maintained |
| **King et al., 2021, United States** | Walking minutes; physical activity; quality of life; physical function | Walking minutes | Quality of life, physical function | Significant increase in walking and physical activity (p < 0.05) | Walking minutes and physical activity increased |
| **Kohn et al., 2023, United States** | Depressive symptoms; anxiety; resilience; sleep disturbances | Depressive symptoms; anxiety | Resilience, sleep disturbances | Significant improvements in depressive symptoms and anxiety; greater resilience (p < 0.05) | Depressive symptoms and anxiety improved; resilience increased |
| **Lamb et al., 2020, United Kingdom** | Fracture rates; falls; health-related quality of life (EQ-5D) | Fracture rates | Falls, health-related quality of life (EQ-5D) | Significant improvement in HRQoL (EQ-5D, p < 0.05); fracture rates: no significant change; falls: no significant change | EQ-5D HRQoL improved; fracture rates: no significant change; falls: no significant change |
| **Lee et al., 2023, Japan** | Cognitive function (logical memory); physical function; social engagement; step count | Logical memory | Physical function, social engagement | Significant improvements in memory, physical function, and social engagement (p < 0.05) | Logical memory, physical function, and social engagement improved |
| **Liang et al., 2021, Taiwan** | Cognitive function (MoCA); frailty score; handgrip strength; gait speed | Cognitive function (MoCA); frailty score | Handgrip strength, gait speed | Significant improvement in MoCA scores and frailty score (p < 0.05) | MoCA scores and the frailty index improved |
| **Liao et al., 2018, China** | Depressive symptoms (GDS) | Depressive symptoms (GDS) | None reported | Significant improvement in GDS scores (p < 0.001) | No direct independence measure reported |
| **Loh et al., 2015, Malaysia** | Physical performance; quality of life (HRQoL); depression | Physical performance | Quality of life, depression | Significant improvements in physical performance and quality of life (p < 0.05) | Physical performance improved; HRQoL improved |
| **Lu et al., 2015, China** | Blood pressure; hypertension knowledge; lifestyle compliance | Blood pressure | Hypertension knowledge, lifestyle compliance | Significant improvements in blood pressure control and lifestyle adherence (p < 0.001) | Blood pressure controlled; lifestyle adherence improved |
| **Marconcin et al., 2022, Portugal** | Self-efficacy; physical activity; balance; agility; quality of life | Self-efficacy | Balance, agility, quality of life | Significant improvements in self-efficacy, balance, and physical activity (p < 0.05); quality of life: no significant change | Self-efficacy and balance improved; physical activity increased |
| **Markle-Reid et al., 2006, Canada** | Mental health (SF-36); depressive symptoms (CES-D); social support (PRQ) | Mental health (SF-36) | Depressive symptoms, social support | Significant improvements in mental health and social support (p < 0.05) | Mental health (SF-36) and social support improved |
| **Marquez et al., 2014, United States** | Physical activity; self-efficacy; physical function; cognitive function; disability | Physical activity | Physical function, cognitive function, and self-efficacy | Significant improvements in physical activity, self-efficacy, and cognitive function (p < 0.05) | Physical activity increased; cognitive function improved |
| **Marquez et al., 2017, United States** | Cognitive function (episodic memory, global cognition, executive function) | Episodic memory | Global cognition, executive function | Significant improvement in episodic memory (p < 0.05); global cognition improved in both groups | Episodic memory improved |
| **Martín-Valero et al., 2013, Spain** | Health-related quality of life (EQ-5D); cardiopulmonary function | Health-related quality of life (EQ-5D) | Cardiopulmonary function | Significant improvement in quality of life in men (p = 0.05); cardiopulmonary function: no significant change | EQ-5D HRQoL improved (men only) |
| **Meng et al., 2024, China** | Dementia risk score; cognitive composite Z score; social isolation; dementia literacy | Dementia risk score; cognitive composite Z score | Social isolation, dementia literacy | Significant reduction in dementia risk score and improvement in cognitive composite Z score (p < 0.001); loneliness reduced | Dementia risk score reduced; cognitive composite Z score improved |
| **Metzner et al., 2023, Germany** | Functional health (WHODAS 2.0); depressive symptoms (PHQ-9); HRQoL; life satisfaction | Functional health (WHODAS 2.0); depressive symptoms (PHQ-9) | HRQoL, life satisfaction | Functional health (WHODAS) declined; depressive symptoms worsened (p < 0.001); no significant between-group difference in primary outcomes | WHODAS functional health declined; depressive symptoms worsened; no significant intervention effect |
| **Mitchell et al., 2006, United States** | Supplement use (multivitamin, calcium); medication management | Multivitamin use | Calcium supplement use, medication management | Significant improvement in multivitamin use and medication management (p < 0.05); calcium use: no significant change | Medication management improved; no direct independence measure reported |
| **Moore-Harrison et al., 2008, United States** | Aerobic capacity (peak VO2); physical function | Peak aerobic capacity; physical function | Self-reported physical function | Significant improvement in aerobic capacity (+18.9%) and physical function (+25%) compared with controls (p < 0.05) | Aerobic capacity and physical function improved |
| **Morone et al., 2016, United States** | Disability (Roland-Morris Disability Questionnaire); pain scale; quality of life; pain self-efficacy | Disability (Roland-Morris); pain | Quality of life, pain self-efficacy | Significant improvement in disability and pain at 8 weeks (p < 0.05); sustained pain reduction at follow-up | Disability (Roland-Morris) and pain scale scores improved |
| **Murphy et al., 2008, United States** | Physical activity; pain; arthritis self-efficacy; physical function | Physical activity; pain | Arthritis self-efficacy, physical function | Significant increase in physical activity in AST group (p < 0.05); trend toward improved pain and physical function | Physical activity increased; arthritis self-efficacy improved |
| **Ng et al., 2017, Singapore** | Depressive symptoms; frailty components (gait speed, energy) | Depressive symptoms | Frailty components (gait speed, energy) | Significant reduction in depressive symptoms at 6 months (p < 0.05); frailty components: no significant change | Frailty components: no significant change; no direct independence measure reported |
| **Nikolaus & Bach, 2003, Germany** | Fall rates; compliance with home modifications | Fall rates | Compliance with modifications | Significant reduction in fall rates (p < 0.05) | Fall rates reduced |
| **Oh et al., 2017, South Korea** | Physical performance (SPPB); muscle quality; gait speed; flexibility | SPPB score; muscle strength | Gait speed, flexibility | Significant improvements in SPPB, muscle quality, and gait speed (p < 0.05) | SPPB, muscle quality, and gait speed improved |
| **Oh et al., 2021, South Korea** | Mobility function; body composition; pain and stiffness (WOMAC) | Mobility function | WOMAC pain, body composition | Significant improvements in mobility function (p < 0.05); WOMAC pain was maintained | Mobility function improved; WOMAC pain maintained |
| **Pahor et al., 2006, United States** | Physical performance (SPPB); walking speed (400-m); mobility disability (MMD) | SPPB score | 400-m walk speed, major mobility disability (MMD) | Significant improvements in SPPB and 400-m walking speed (p < 0.001); reduced MMD incidence | SPPB and 400-m walk speed improved; MMD incidence reduced |
| **Parial et al., 2023, Philippines** | Global cognition (MoCA); executive function; mobility; quality of life (HRQoL) | Global cognition (MoCA) | Executive function, mobility, quality of life | Significant improvements in cognition and quality of life (p < 0.05); moderate to large effect sizes | MoCA scores improved; HRQoL improved |
| **Park et al., 2011, South Korea** | Systolic blood pressure; exercise self-efficacy; quality of life | Systolic blood pressure | Exercise self-efficacy, quality of life | Significant reduction in blood pressure; improvements in self-efficacy (p < 0.05) | Blood pressure reduced; self-efficacy improved |
| **Piedra et al., 2018, United States** | Pedometer-measured step counts; physical activity; self-efficacy | Pedometer-measured steps | Self-efficacy, expectations about aging | Significant increase in step counts at 12 months (p < 0.05); no sustained effect at 24 months | Step counts increased at 12 months; no sustained effect at 24 months |
| **Piette et al., 2023, United States** | Perceived cognitive change (Cognitive Change Index) | Cognitive Change Index | Session satisfaction | Significant improvement in Cognitive Change Index (p < 0.05) | Cognitive Change Index improved |
| **Quach et al., 2022, Canada** | Mobility disability (MMD); frailty trajectory | Major mobility disability (MMD) | Frailty trajectory | Significant reduction in MMD in frail participants (p < 0.05); frailty trajectories similar across groups | MMD risk reduced |
| **Reed et al., 2018, Australia** | Self-rated health; health behavior | Self-rated health | Health status, health behavior | Significant improvement in self-rated health (p < 0.05); no significant differences in secondary outcomes | Self-rated health improved |
| **Reid et al., 2019, United States** | Physical performance (SPPB); executive function; grip strength; quality of life | SPPB score | Executive function, grip strength, quality of life | Significant improvements in SPPB and executive function (p < 0.05) | SPPB improved; executive function improved |
| **Rejeski et al., 2017, United States** | 400-m walk time; knee strength; BMI; physical activity | 400-m walk time | Knee strength, BMI, and physical activity | Significant improvement in 400-m walk time in combined groups (p < 0.001); knee strength was preserved in exercise groups | 400-m walk time improved; knee strength preserved |
| **Rubenstein et al., 1994, United States** | Health status; functional status; health service utilization | Health status; functional status | Psychosocial parameters | Improvements in health and functional status were described in the source publication; however, no statistical estimates were available for extraction. | Functional status improved |
| **Shake et al., 2018, United States** | Functional performance (upper and lower body strength, gait speed); cognition; health knowledge | Upper body strength; lower body strength | Gait speed, health knowledge, and cognitive performance | Significant improvements in upper and lower body strength and cognitive performance (p < 0.05) | Upper and lower body strength and cognitive function improved |
| **Sheffield et al., 2013, United States** | Home safety; functional status; fear of falling; HRQoL | Home safety; functional status | Fear of falling, HRQoL | Significant improvements in home safety and reduced fear of falling (p < 0.05); functional status: no significant improvement | Home safety improved; fear of falling reduced; functional status: no significant change |
| **Shumway-Cook et al., 2007, United States** | Falls incidence; mobility; balance; leg strength | Falls incidence | Balance, leg strength, mobility | Significant improvements in balance, leg strength, and mobility (p < 0.05); falls reduced by 25% (not statistically significant in intention-to-treat analysis) | Balance, leg strength, and mobility improved; falls reduced (non-significant in ITT analysis) |
| **Shvedko et al., 2020, United Kingdom** | Loneliness; physical activity; social support; depression; self-efficacy | Loneliness | Social support, depression, and self-efficacy | Improvements in social networks and self-efficacy were observed; effects were not statistically significant | Social network and self-efficacy improvements observed; results not statistically significant |
| **Smail et al., 2023, United States** | Mobility disability (MMD); depressive symptoms | Major mobility disability (MMD) | Depressive symptoms | Significant reduction in MMD risk (p < 0.05); no differential effect by depressive symptom status | MMD risk reduced |
| **Smith-Ray et al., 2014, United States** | Balance (BBS); gait speed (10MGS); distracted gait speed | Balance (BBS); gait speed (10MGS) | Distracted gait speed | Significant improvements in balance and gait speed (p < 0.05); distracted gait speed: no significant change | Balance (BBS) and gait speed (10MGS) improved |
| **Song & Yu, 2019, China** | Cognitive function (MoCA-C); quality of life; depressive symptoms; sleep quality | Cognitive function (MoCA-C) | Health-related quality of life, depressive symptoms, and sleep quality | Significant improvements in cognitive function and quality of life (p < 0.001) | Cognitive function (MoCA-C) improved; quality of life improved |
| **Song et al., 2024, China and Hong Kong** | Sleep quality (PSQI); cognitive function (MoCA) | Sleep quality (PSQI) | Cognitive function (MoCA) | Significant improvements in sleep quality (p < 0.05) and cognitive function (p < 0.05) | Sleep quality (PSQI) and cognitive function (MoCA) improved |
| **Spoorenberg et al., 2018, Netherlands** | Health (EQ-5D-3L); well-being; self-management knowledge | Health (EQ-5D-3L) | Well-being, self-management knowledge | Improvement in self-management knowledge (p < 0.01); EQ-5D and well-being: no significant change | Self-management knowledge improved; EQ-5D and well-being: no significant change |
| **Stuck et al., 1995, United States** | ADL dependence; nursing home admissions; hospital admissions | Disability prevention (ADL dependence) | Nursing home admissions, hospital admissions | Significant reduction in need for assistance with ADLs (p < 0.05) | ADL dependence reduced |
| **Stuck et al., 2000, Switzerland** | ADL dependence; nursing home admissions; health status; medication use | ADL dependence | Health status, medication use, and nursing home admissions | Significant reduction in ADL dependence in low-risk participants (p < 0.05); no benefit in high-risk elderly | ADL dependence reduced (low-risk participants) |
| **Sugiyama et al., 2015, United States** | Mental health (MCS-12); HbA1c; social support | Mental health (MCS-12) | HbA1c, social support | Significant improvement in mental health (p < 0.05); independent of glycemic control and social support | Mental health (MCS-12) improved |
| **Szanton et al., 2011, United States** | ADL/IADL difficulty; quality of life; falls efficacy | ADL/IADL difficulty | Quality of life, falls efficacy | Significant improvements in ADL/IADL difficulty and quality of life (p < 0.05); effect sizes 0.55–0.89 | ADL/IADL difficulty reduced; quality of life improved |
| **Szanton et al., 2014, United States** | ADL/IADL difficulty; physical performance (SPPB); home safety; healthcare utilization | ADL/IADL difficulty | SPPB, home safety, healthcare utilization | Significant improvements in ADL/IADL difficulty and SPPB (p < 0.05); home safety improved | ADL/IADL difficulty reduced; SPPB improved; home safety improved |
| **Taylor et al., 2016, United Kingdom** | Pain-related disability; depression; anxiety; self-efficacy; social integration | Pain-related disability | Depression, anxiety, self-efficacy, social integration | Significant improvement in pain-related disability (p < 0.05); significant improvements in depression and social integration at 12 months | Pain-related disability improved; no direct ADL/IADL or SPPB measure reported |
| **Uemura et al., 2018, Japan** | Health literacy (HLS-14); cognitive function; physical function (gait speed, balance); physical activity; dietary habits | Health literacy (HLS-14) | Physical function, dietary habits, cognitive function | Significant improvements in health literacy, cognitive function, gait speed, balance, and physical activity (p < 0.05) | Health literacy (HLS-14) and cognitive function improved; physical activity increased |
| **Vaz Fragoso et al., 2015, United States** | Sleep quality (PSQI); insomnia severity (ISI); daytime drowsiness (ESS) | Poor sleep quality (PSQI > 5) | Insomnia severity (ISI), daytime drowsiness (ESS) | Physical activity associated with lower likelihood of poor sleep quality (OR ≈ 0.80, p < 0.05); ISI and ESS: no significant change | Sleep quality (PSQI) improved; no direct independence measure reported |
| **Wang et al., 2016, China** | Bone mineral density (BMD); osteoporosis knowledge; health beliefs; quality of life; medication compliance; pain | Bone mineral density (BMD) | Quality of life, medication compliance, pain, health beliefs | Significant improvements in BMD and quality of life (p < 0.001); medication compliance and health knowledge improved | BMD improved; quality of life improved; medication compliance increased |
| **Wolf et al., 1996, United States** | Falls incidence; fear of falling; grip strength; flexibility; blood pressure; well-being | Fear of falling; grip strength | Flexibility, blood pressure, body composition, well-being | Tai Chi reduced the risk of multiple falls by 47.5% (p < 0.05); grip strength and systolic blood pressure improved (p < 0.05) | Fear of falling reduced; grip strength and flexibility improved |
| **Wong et al., 2020, Hong Kong** | Health-related quality of life (HRQoL; mental component); ADL; depressive symptoms | HRQoL (mental component) | ADL, depressive symptoms | Significant improvements in mental HRQoL and ADL (p < 0.05) | ADL scores improved; mental HRQoL improved |
| **Wong et al., 2022, Hong Kong** | Quality of life; self-efficacy; pain levels; systolic blood pressure; depression | Quality of life | Self-efficacy, blood pressure, pain levels, depression | Significant improvements in self-efficacy and pain levels in mHealth plus integrated care group (p < 0.05); quality of life: no significant change | Self-efficacy improved; pain levels reduced |
| **Woo et al., 2024, Taiwan** | Frailty index; physical fitness; IADL; quality of life; heart rate variability (HRV) | Frailty index | IADL, quality of life, HRV | Significant reductions in frailty index and improved physical fitness (p < 0.05); IADL and quality of life improved | Frailty index reduced; physical fitness improved; IADL improved |
| **Wu et al., 2019, Taiwan** | Self-efficacy; BMI; blood pressure; blood glucose; cholesterol | Self-efficacy; BMI | Blood glucose, cholesterol | Significant improvements in self-efficacy, BMI, and blood pressure (p < 0.05) | Self-efficacy and health markers improved |
| **Xu et al., 2020, China** | Health-related quality of life (EQ-5D-3L); anxiety; depression; health knowledge; blood pressure | HRQoL (EQ-5D-3L) | Health knowledge, lifestyle compliance, anxiety, depression | Significant improvements in HRQoL and health knowledge (p < 0.05); reduced anxiety and depression | EQ-5D HRQoL improved; health knowledge improved |
| **Yang et al., 2023, China** | Swallowing function; quality of life; depressive symptoms | Swallowing function | Depressive symptoms, quality of life | Significant improvements in swallowing function and quality of life (p < 0.05); depressive symptoms reduced | Swallowing function improved; quality of life improved |
| **Zhao et al., 2023, China** | Executive function; working memory; psychomotor speed | Executive function | Working memory, psychomotor speed | Significant improvements in executive function, working memory, and psychomotor speed (p < 0.05); benefits sustained at 3-month follow-up | Executive function, working memory, and psychomotor speed improved |

**Note:** Some randomized controlled trials were reported in multiple publications; therefore, the number of publications (n = 91) exceeds the number of unique trials (n = 85). Some publications reported secondary analyses of previously conducted randomized trials. These were retained because they reported additional intervention outcomes relevant to the review objectives. Independence-related outcomes include direct measures of functional independence (e.g., ADL/IADL, mobility disability, SPPB, MMD) where reported. When direct measures were not reported, closely related functional performance indicators (e.g., gait speed, functional fitness, balance) and determinants closely linked to independent living (e.g., self-efficacy, self-determination) are summarized as reported in the source publication. Proxy outcomes were included only when they have established empirical associations with functional independence in gerontological research. Main reported results are summarized at the individual study level as reported by study authors and should not be interpreted as pooled effect estimates. This approach allowed consistent interpretation of heterogeneous outcome measures in relation to the overarching concept of functional independence in later life.

**Abbreviations:** ABC = Activities-specific Balance Confidence Scale; ADL = activities of daily living; AST = activity strategy training; BBS = Berg Balance Scale; BMD = bone mineral density; BMI = body mass index; CCTS = Composite Cognitive Test Score; CES-D = Center for Epidemiologic Studies Depression Scale; CHAMPS = Community Healthy Activities Model Program for Seniors; DALY = disability-adjusted life year(s); EQ-5D = EuroQol 5-Dimension questionnaire; ESS = Epworth Sleepiness Scale; FES = Falls Efficacy Scale; FRT = Functional Reach Test; GDS = Geriatric Depression Scale; HLS-14 = Health Literacy Scale-14; HR = hazard ratio; OR = odds ratio; peak VO2 = peak oxygen uptake; HRQoL = health-related quality of life; HRV = heart rate variability; IADL = instrumental activities of daily living; IPOS = Integrated Palliative Outcome Scale; ISI = Insomnia Severity Index; ITT = intention-to-treat; JKOM = Japanese Knee Osteoarthritis Measure; MCS-12 = Mental Component Summary-12; MMD = major mobility disability; MoCA = Montreal Cognitive Assessment; PAR = Physical Activity Recall; PHQ-9 = Patient Health Questionnaire-9; PRQ = Personal Resource Questionnaire; PSQI = Pittsburgh Sleep Quality Index; QALY = quality-adjusted life year(s); QoL = quality of life; SF-12/SF-36 = Short Form Health Survey (12-item/36-item versions); SFT = Senior Fitness Test; SPPB = Short Physical Performance Battery; TUG = Timed Up and Go test; 10MGS = 10-Metre Gait Speed; VAS = Visual Analogue Scale; WHODAS = World Health Organization Disability Assessment Schedule; WOMAC = Western Ontario and McMaster Universities Osteoarthritis Index.
