# Supplementary material for: Community-based interventions to support aging in place and functional independence in older adults: a systematic review of randomized controlled trials
Source: Front Public Health. 2026 May 15;14:1828271. doi: 10.3389/fpubh.2026.1828271 (PMC13219341; doi:10.3389/fpubh.2026.1828271)
Supplement: Supplementary file 4 [file Table_4.DOCX]

**Supplementary Table 4. Scalability, Sustainability, and Practice Implications of Community-Based Public Health Interventions**

*Total included studies: 91 publications representing 85 independent randomized controlled trials.*

| **Author(s), Year, Country** | **Scalability and Sustainability Factors** | **Potential Challenges** | **Community Involvement** | **Implications for Practice** | **Future Research Directions and Recommendations** |
| --- | --- | --- | --- | --- | --- |
| Acton et al., 2016, United Kingdom | Training of visual rehabilitation officers may support broader scalability; long-term sustainability may require continued funding and integration with health services. | Shortage of trained professionals; lack of statutory requirements; funding constraints. | Home visits by visual rehabilitation officers; delivery in participants' homes. | Home visit–based visual rehabilitation may be considered for further evaluation as a component of integrated care for individuals with low vision. | Larger trials are needed to assess mental health components and conduct cost-effectiveness analysis. |
| Arai et al., 2007, Japan | Minimal equipment requirements may facilitate broader scalability; continued personnel and resource support may be needed for long-term sustainability. | Maintaining participant engagement in long-term programs; adapting exercises to varying physical abilities. | Recruitment from local communities; collaboration with community institutions. | Short-term exercise interventions targeting individuals with lower baseline falls self-efficacy showed potential benefits in this trial. | Research should investigate long-term effects on falls self-efficacy, subgroup benefits, and psychological aspects of falls prevention. |
| Bae et al., 2019, Japan | Use of non-professional staff and existing community resources may facilitate scalability; high participant satisfaction observed in the trial may contribute to long-term sustainability. | Maintaining consistent engagement in long-term programs; ensuring adequate cognitive load in activities. | Delivery in familiar community spaces; participant engagement in program activities. | The multicomponent intervention reported potential benefits for spatial working memory and physical activity maintenance in older adults with MCI. | Further research should assess long-term impacts on dementia prevention and refine activities to provide sufficient cognitive and physical challenge. |
| Bann et al., 2016, United States | Use of supervised clinical centers and home-based activities may support broader scalability; long-term sustainability may require ongoing community resources. | Maintaining consistent engagement across socioeconomic groups; sustaining long-term motivation. | Access to local clinical centers; transportation services provided to participants. | Structured physical activity programs reported potential benefits for reducing mobility disability in older adults. | Future studies should evaluate long-term impacts and explore adjustments to better address socioeconomic disparities in effectiveness. |
| Brown et al., 2020, United States [Secondary analysis of the LIFE trial cohort] | SPPB-based assessment requires minimal equipment and may facilitate scalability of monitoring approaches if adopted in routine practice; this row reflects a secondary analysis, not a primary implementation trial. | Maintaining motivation in higher-functioning participants; safety monitoring requirements during physical activity delivery. | Intervention delivered in accessible community locations as part of the LIFE trial infrastructure. | Secondary analysis reported that SPPB decline was associated with increased subsequent risk of MMD; findings may inform routine physical function monitoring in older adults. | Research should examine SPPB tracking for early identification of mobility decline and assess the impact of concurrent health events on mobility outcomes. |
| Chao et al., 2012, China | Existing community health infrastructure may support scalability; sustainability may be supported by ongoing staff training and participant engagement. | Sustaining participant engagement; managing resource allocation over time. | Delivery through regular health check-ups and educational sessions in community settings. | Community-based health management programs reported potential benefits for health outcomes and reduction in outpatient visits in older adults. | Long-term studies are needed to assess effects on hospital admissions, mortality, and the most effective intervention components. |
| Chen et al., 2021, Taiwan | Low equipment requirements may facilitate scalability; potential sustainability requires trained instructors and regular sessions. | Maintaining adherence to the exercise program. | Delivery through local health centers; group-based exercise sessions. | Tai Chi reported potential benefits for functional fitness in older adults with mild knee osteoarthritis. | Future research should investigate long-term effects of Tai Chi on functional fitness, quality of life, and psychosocial health. |
| Clark et al., 1997, United States | Scalability may require trained occupational therapists; long-term sustainability may require continued participant engagement. | Maintaining participant adherence and engagement. | Delivery through apartment complexes; therapist-led home visits. | Preventive occupational therapy reported potential benefits for health and well-being in independent-living older adults. | Research should explore occupational therapy in diverse living situations and socioeconomic contexts. |
| Clark et al., 2002, United States | Technology-based delivery may support broad scalability; potential sustainability requires ongoing community partnerships. | Overcoming barriers related to literacy and language; sustaining engagement. | Local community partnerships and advisory committees. | TTM-based interventions reported potential for promoting multiple health behaviors in older adults. | Further research should assess long-term sustainability and effectiveness across diverse populations and settings. |
| Ćwirlej-Sozańska et al., 2018, Poland | Low-cost equipment (Thera-Bands) may facilitate scalability if implemented in routine practice; sustainability may be supported by availability of trained physiotherapists. | Maintaining long-term participant adherence; securing funding for sustained programs. | Involvement of the local community and public health institutions. | Multifactorial exercise combined with health education reported potential benefits for functional fitness and balance in older adults. | Long-term studies should assess sustained impact, including delivery in low-income communities and via home-based formats. |
| Ekelund & Eklund, 2015, Sweden | Multi-professional team structure may be adaptable to other settings; long-term sustainability may require sustained team engagement. | Maintaining long-term case manager support and follow-up. | Older people are actively involved in decision-making within the program. | Person-centered integrated care reported potential for slowing the decline in self-determination in frail older adults. | Research should explore transferability to other settings and populations, including those with cognitive impairments or dementia. |
| Eklund et al., 2008, Sweden | Adaptable program format may support scalability across settings; sustainability may be supported by ongoing facilitator training. | Participant retention challenges during follow-up. | Participant engagement through group discussions and strategy learning. | Group-based health-promotion programs reported potential benefits for ADL independence in older adults with visual impairments. | Future studies should use longer follow-up periods, robust methods to minimize dropout, and blinded outcome assessment. |
| Endevelt et al., 2011, Israel | Dietitian-led format may support scalability across healthcare settings; potential sustainability requires ongoing training. | Participant retention challenges during follow-up. | Involvement of family members and caregivers in dietary counseling sessions. | Intensive dietary intervention reported potential benefits for cognitive function and nutritional status in older adults. | Larger, long-term studies are needed to confirm findings and assess the sustained impact of dietary interventions. |
| Estebsari et al., 2018, Iran | Urban health center infrastructure may support scalability; long-term sustainability may require reinforced education and training. | Maintaining participant engagement over time; ensuring consistent program delivery. | Structured educational sessions; limited participant involvement beyond program attendance. | Empowerment-focused educational interventions reported potential benefits for preventing elder abuse and promoting healthy behaviors. | Further studies should focus on long-term outcomes, cultural adaptations, and strategies for sustaining intervention effects. |
| Evans et al., 2021, United Kingdom | Integration with existing health services may support scalability; potential sustainability requires collaboration between care teams and nursing staff. | Lack of electronic health record sharing between services; potential high resource demand. | Multidisciplinary collaboration between nursing, palliative care, and community care teams. | SIPScare reported reduced symptom distress and potential cost-effectiveness; findings should be interpreted within the context of the specific trial setting and population. | Future trials should include ethnically diverse populations, expand to more care homes, and explore mechanisms to enhance integration and sustainability. |
| Feng et al., 2020, Singapore | Singing programs may be feasible in community settings; potential sustainability could be supported by integration with existing health programs. | Sustaining participant engagement; maintaining program quality in community settings without specialized music training. | Active participation in group singing sessions. | Choral singing reported potential benefits for cognitive outcomes in older adults; larger trials are needed before broader conclusions can be drawn. | Larger-scale RCTs are needed to confirm effectiveness, explore long-term cognitive impact, and identify aging biomarkers. |
| Fielding et al., 2017, United States | Standardized physical activity programs may support scalability; sustainability may be supported by sustained participant engagement. | Maintaining long-term participant adherence and motivation. | Not reported in the source publication. | Structured physical activity programs reported potential benefits for reducing disability and improving physical function in older adults. | Future studies should explore long-term sustainability and strategies to improve adherence to community-based physical activity programs. |
| Giné-Garriga et al., 2013, Spain | Primary care setting may support scalability; some evidence of long-term improvements may inform sustainability in routine practice. | Adherence; sustaining engagement across diverse community settings. | Visits to community resources to facilitate continued physical activity. | Functional circuit training reported potential benefits for fear of falling and health status in frail older adults. | Further research should assess long-term adherence and applicability to broader older adult populations. |
| Gitlin et al., 2006, United States | Low-cost delivery may support scalability; benefits sustained at 12 months may inform sustainability in routine practice. | Scaling to diverse communities may require cultural adaptation and additional training. | Home visits by occupational and physical therapists; community-based delivery. | The intervention reported potential benefits for reducing functional decline and improving quality of life in older adults. | Future research should assess long-term cost-effectiveness, include objective performance measures, and evaluate differential subgroup benefits. |
| González-Guerrero et al., 2014, Spain | Geriatric day care hospital setting may support scalability; potential sustainability informed by patient satisfaction and program feasibility. | Adaptation may be needed across different healthcare settings. | Multidisciplinary team including geriatricians, nurses, and social workers. | Disease management programs in geriatric day care settings reported potential benefits for reducing hospital readmissions and improving quality of life in patients with heart failure. | Future studies should assess long-term cost-effectiveness, broader population applicability, and the role of specialist involvement. |
| Groessl et al., 2016, United States | Multi-center delivery format may support scalability; replicable program design may contribute to long-term sustainability. | Higher transportation-related costs associated with physical activity intervention. | Participants engaged through regular physical activity sessions and home-based activities. | Physical activity intervention reported potential cost-effectiveness for preventing mobility disability and may inform future implementation planning. | Research should explore strategies to reduce costs and assess long-term sustainability in broader settings. |
| Guerrero et al., 2020, United States | Multi-site delivery may support scalability; long-term sustainability may require ongoing local facilitator engagement. | Recruitment and retention difficulties; protocol adherence challenges. | Partnership with local senior centers and community facilitators. | AMP reported potential improvements in mental health and social satisfaction; positive findings were in as-treated rather than intention-to-treat analysis and should be interpreted with caution. | Process evaluations are needed to improve fidelity and explore cultural adaptations for diverse populations. |
| Hernandez et al., 2019, United States | Senior center infrastructure may support scalability; sustainability may be supported by continued staff and facilitator engagement. | Participant retention challenges; sustaining engagement across 24 months. | Partnership with local senior centers and staff; delivery in community settings. | Both groups reported improvements in depressive symptoms; between-group differences were not statistically significant; findings may be context-dependent. | Future research should explore modifications to attribution retraining to enhance impact on depressive symptoms, particularly in frailer populations. |
| Janevic et al., 2022, United States | CHW-led and telehealth delivery may support scalability; integration with Medicaid may enhance potential sustainability. | Maintaining participant engagement; training and supporting CHWs; adapting to different community contexts. | CHWs recruited from participant communities; culturally tailored program content. | Positive STEPS reported potential benefits for chronic pain management in African American older adults. | Larger efficacy trials are needed to confirm findings, explore long-term outcomes, and test combined positive activity and pain self-management approaches. |
| Johnson et al., 2018, Canada | Home care infrastructure may support scalability; long-term sustainability may require continued support worker training and monitoring. | Recruitment challenges in rural areas; complex intervention coordination. | Partnership with the VON program and local health authority; home-based delivery. | Home-based exercise intervention reported potential benefits for functional capacity and well-being in frail rural older adults. | Research should explore long-term effects and compare outcomes between rural and urban settings. |
| Jones et al., 2019, Canada | Partnerships with community organizations may support scalability; use of non-audiologists may enhance accessibility. | Sustaining participant engagement; scaling beyond initial delivery settings. | Participant involvement in design and recruitment; partnership with community organizations. | Intervention combining exercise and GAR reported potential benefits for fitness and loneliness reduction in older adults with hearing loss. | Larger, long-term RCTs should assess enduring effects of GAR, particularly with more focused balance training and psychosocial components. |
| Keall et al., 2017, New Zealand | Low-cost format may support broader scalability; cost savings from injury reduction may contribute to long-term sustainability. | High upfront costs for national rollout; variability in housing stock. | Involvement of eligible households; community-based delivery. | Home modifications reported potential cost-effectiveness for reducing fall-related injuries. | Research should target high-risk groups, assess long-term impacts, and explore integration into national public health strategies. |
| Khodneva et al., 2021, United States | Peer-delivered format may support scalability in rural settings; cultural adaptation may enhance potential sustainability. | Maintaining participant engagement; training and supporting peer supporters. | Peer supporters recruited from participant communities; culturally adapted content. | Peer-delivered CBT reported potential benefits for pain management and functional outcomes in rural adults with chronic pain. | Future studies should explore long-term impacts on diabetes management, expansion to other chronic conditions, and methods to sustain peer support. |
| Kim et al., 2013, Japan | Ease of delivery may facilitate scalability if implemented in routine practice; potential sustainability requires community engagement and resources. | Maintaining adherence and participant motivation. | Delivery through local community centers and health promotion teams. | Combined exercise and thermal therapy reported potential benefits for pain reduction and functional mobility in older women with knee pain. | Research should investigate long-term effects of non-pharmacologic pain management and compare approaches. |
| Kim et al., 2016, Japan | Low-cost delivery via community centers may support scalability; sustainability may be supported by access to regular classes and nutritional supplements. | Adherence to exercise programs; access to nutritional supplements. | Regular participation in community exercise classes. | Combined exercise and nutritional intervention reported potential benefits for physical function and body composition in older adults with sarcopenic obesity. | Long-term studies should assess effects on sarcopenic obesity, separate impacts of exercise versus nutrition, and implementation in diverse populations. |
| King et al., 2007, United States | Automated telephone format may support low-cost scalability; long-term sustainability remains uncertain without further evidence. | Technological issues with the automated system; long-term adherence to physical activity. | No specific community involvement reported. | Automated telephone-based physical activity advice reported potential for promoting activity; human interaction may further enhance outcomes. | Further research should assess long-term sustainability, cost-effectiveness, and combined human–automated delivery approaches. |
| King et al., 2017, United States | Delivery in urban and suburban settings may support scalability with neighborhood adjustments; potential sustainability requires adaptable community engagement. | Reductions in active transport were observed in less compact neighborhoods; additional targeting strategies may be needed. | Participants were recruited from community settings; engagement through outreach activities. | Structured physical activity programs reported increased leisure walking; compensatory reductions in routine activity were also observed. | Research should explore the integration of active transport promotion with structured physical activity to address compensatory activity reductions. |
| King et al., 2021, United States | Community partnership and housing site structure may support scalability; resident empowerment as citizen scientists may contribute to long-term sustainability. | Maintaining participant engagement over time; resource constraints. | Residents actively involved in identifying environmental issues and advocating for neighborhood changes. | Citizen science initiative reported potential for complementing physical activity programs and supporting health behavior change. | Future studies should assess the broader applicability of citizen science models, with a focus on long-term impacts on health equity. |
| Kohn et al., 2023, United States | Minimal equipment requirements may facilitate scalability if implemented in routine practice; high adherence observed in the trial may inform sustainability in routine practice. | Need for qualified instructors and sustained participant engagement. | Group-based sessions in community settings. | Tai Chi reported potential benefits for mental health and resilience in older adults; findings should be interpreted within the specific implementation context of the crisis-period trial setting. | Research should explore the remote delivery of Tai Chi and investigate long-term physical and mental health impacts. |
| Lamb et al., 2020, United Kingdom | NHS integration may support scalability; existing infrastructure may contribute to long-term sustainability. | Low uptake of multifactorial components; delays in intervention start times. | Delivery within existing general practice settings. | Exercise intervention reported potential benefits for quality of life and potential cost-effectiveness; fracture outcomes were not significantly improved in this trial. | Future studies should explore more intensive interventions, integrate bone health strategies with fall prevention, and assess long-term adherence. |
| Lee et al., 2023, Japan | Community-based delivery format may support scalability; high adherence observed in the trial may inform sustainability in routine practice. | Difficulty maintaining engagement over longer periods. | Participant engagement in exercise and social activities within local community settings. | Multidomain intervention reported potential benefits for preventing cognitive decline in older adults. | Further research is needed on the long-term effects of dementia prevention, including consideration of genetic risk factors such as the apolipoprotein E ε4 genotype. |
| Liang et al., 2021, Taiwan | Community health program integration may support scalability; high adherence observed in the trial may inform sustainability in routine practice. | Maintaining long-term participant engagement. | Delivery through community centers; group-based program activities. | Multidomain intervention reported potential benefits for cognitive and physical function in older adults. | Future studies should assess large-scale implementation for older adults with PCDS and evaluate long-term effects across diverse populations. |
| Liao et al., 2018, China | Group-based, low-cost format may support scalability; cultural relevance may contribute to long-term sustainability. | Limited availability of trained Tai Chi instructors. | Culturally adapted group delivery; participant engagement in program activities. | Combined music and Tai Chi reported potential benefits for depressive symptoms in older adults. | Long-term studies should explore broader applicability across diverse populations and implementation contexts. |
| Pahor et al., 2006, United States | Multicenter trial structure may inform scalability in routine practice settings; transition to home-based exercise may contribute to long-term sustainability. | Long-term adherence to physical activity; safety monitoring requirements during program delivery. | Community centers used for initial program phases; ongoing engagement through counseling. | Structured physical activity programs reported potential improvements in physical performance and the potential for reducing mobility disability in older adults. | Larger, longer-term RCTs are needed to assess the impact on major mobility disability and broader health outcomes. |
| Loh et al., 2015, Malaysia | Community-based and home-exercise format may support scalability; ongoing support may contribute to long-term sustainability. | Maintaining long-term adherence; limited resources in target communities. | Community engagement facilitated through local leaders; participant involvement in program activities. | CERgAS reported potential benefits for physical performance and independence in older adults in urban poor settings. | Future research should explore long-term impacts and adaptation for low-resource settings in Malaysia and similar regions. |
| Lu et al., 2015, China | Community health resources may support scalability; ongoing facilitator involvement may contribute to long-term sustainability. | Maintaining long-term participant engagement; preventing attrition. | Interactive, patient-tailored educational workshops delivered in community settings. | Interactive education workshops reported potential benefits for hypertension management and may inform chronic disease programs in low-resource settings. | Research should focus on integrating this approach with other chronic disease programs and evaluating long-term sustainability. |
| Marconcin et al., 2022, Portugal | Multi-setting delivery may support scalability; long-term sustainability may require continued trainer support. | Maintaining adherence; addressing health constraints during the program. | Group-based sessions; direct participant involvement in program activities. | PLE2NO reported potential benefits for self-efficacy, physical activity, and balance in older adults with arthritis. | Long-term studies are needed to assess adherence, effects, and integration into broader chronic disease management strategies. |
| Markle-Reid et al., 2006, Canada | Home care infrastructure may support scalability; continuity of nursing care may contribute to long-term sustainability. | Participant retention challenges among lower-functioning older adults. | Coordination with community services; nursing-led home care delivery. | Nursing health promotion services reported potential benefits for quality of life in frail older home care clients without increasing costs. | Future trials with larger samples and economic evaluations are needed to assess long-term cost-effectiveness and scalability. |
| Marquez et al., 2014, United States | Peer-led model with community leaders may support scalability; cultural relevance may contribute to long-term sustainability. | Maintaining retention and engagement over 8 months. | Delivery through local senior centers; peer-led program facilitation. | BAILAMOS reported potential benefits for physical activity and health outcomes in older Latino adults. | Multi-site trials are needed to evaluate the program in other urban Latino populations and validate findings across regions. |
| Marquez et al., 2017, United States | Community-based format may support scalability in Latino communities; cultural relevance may contribute to long-term sustainability. | Participant retention challenges due to transportation issues, caregiving responsibilities, and employment changes. | Delivery at a community senior center; bilingual instructor involvement. | BAILAMOS dance program reported potential benefits for cognitive function in older Latinos. | Larger trials should assess long-term sustainability and explore mechanisms underlying cognitive improvements. |
| Martín-Valero et al., 2013, Spain | Primary healthcare center format may support scalability; long-term sustainability may require ongoing professional support. | Participant retention over follow-up; differing engagement patterns across participant groups. | Recruitment and delivery through Primary Healthcare Centers. | PAPP reported potential benefits for quality of life in older inactive individuals, with greater improvement observed in men. | Further studies should explore long-term effects and gender differences, particularly in cardiopulmonary outcomes. |
| Meng et al., 2024, China | WeChat-based and community service delivery may support scalability; low-cost format may contribute to long-term sustainability. | Adherence to cognitive training; need for family support; long-term effectiveness not assessed. | Community worker involvement, volunteer services, and group activity participation. | Multidomain intervention reported potential benefits for dementia prevention in at-risk older adults. | Future studies should assess long-term effects, expand to all age groups, and include family support strategies for cognitive training adherence. |
| Metzner et al., 2023, Germany | Standardized care planning may support scalability; interdisciplinary support may contribute to long-term sustainability. | High care burden on older adults; severe impairments may limit self-management benefits. | Integration of local healthcare providers and formal and informal support networks. | LoChro-Care did not demonstrate significant effects in this trial; modifications may be needed, particularly for patients with severe functional impairments. | Research should explore earlier intervention initiation, more active case management, and adaptation for specific patient needs. |
| Mitchell et al., 2006, United States | Extension agent network may support scalability; integration into existing programs may contribute to long-term sustainability. | Staff turnover; need for consistent implementation fidelity; maintaining participant engagement across delivery sites. | Delivery in community nutrition sites (CN sites) already serving the target population. | Nutrition education intervention reported potential benefits for health behavior change in low-income older adults. | Long-term research is needed to examine effects across multiple interventions and assess behavior change and health outcomes over time. |
| Moore-Harrison et al., 2008, United States | Low-cost equipment requirements may support scalability; high adherence observed in the trial may inform sustainability in routine practice. | Participant retention; limited delivery capacity in a single community site. | Delivery in a community-based public housing complex. | Walking program reported potential benefits for physical function in older adults with low socioeconomic status. | Larger, long-term studies with diverse populations are needed to confirm findings and assess long-term benefits. |
| Morone et al., 2016, United States | Group-based format may support scalability; ongoing support may be required to sustain improvements. | Difficulty maintaining long-term functional improvement without continuous intervention; low booster session attendance. | High participant engagement and satisfaction; group-based delivery. | Mindfulness-based intervention reported potential short-term benefits for function and pain management in older adults with chronic low back pain. | Future research should focus on improving durability of functional improvements and exploring methods to increase long-term engagement. |
| Murphy et al., 2008, United States | Senior housing program infrastructure may support scalability; follow-up sessions may contribute to long-term sustainability. | Maintaining participation in senior housing settings; adaptation needed for broader community settings. | Group sessions and individualized home visits within senior housing settings. | AST combined with exercise reported potential benefits for physical activity and pain in older adults with osteoarthritis. | Larger, long-term trials are needed to validate findings and assess broader applicability for osteoarthritis management. |
| Ng et al., 2017, Singapore | Community program integration may support scalability; home-based exercise component may contribute to long-term sustainability. | Maintaining consistent adherence; sustaining participant motivation over time. | Group sessions combined with home-based exercise; community program delivery. | Multidomain lifestyle intervention reported potential benefits for depressive symptoms in frail older adults. | Research should assess long-term effects of multimodal interventions on depression and physical outcomes in higher-risk frail populations. |
| Nikolaus & Bach, 2003, Germany | Cost-effectiveness findings may support scalability; compliance with home modifications may contribute to long-term sustainability. | Maintaining adherence to modification recommendations; participant motivation. | Family involvement in home modification process. | Multidisciplinary home-based intervention reported potential benefits for fall reduction in frail older adults. | Long-term research should explore sustainability of home modifications and their impact across larger, more diverse populations. |
| Oh et al., 2017, South Korea | Community center–based, low-cost delivery may support scalability; sustainability may be supported by adequate adherence across supervised and self-directed phases. | Participant retention challenges during the self-directed phase, particularly in adverse weather conditions. | Delivery through existing community senior centers; healthcare professional involvement. | HAHA II reported potential benefits for physical function and muscle quality in older women. | Studies should explore long-term adherence to self-directed exercise programs and account for environmental factors such as weather. |
| Oh et al., 2021, South Korea | Low-cost rural delivery may support scalability; ongoing education and follow-up may contribute to long-term sustainability. | Participant retention challenges due to seasonal farming activities; low adherence to home-based exercises. | Delivery through local public health centers; partnership with community organizations. | Integrated exercise and health education program reported potential benefits for mobility in older adults with knee OA in rural settings. | Future research should improve adherence to home-based programs during seasonal changes and evaluate long-term effects on mobility and quality of life. |
| Parial et al., 2023, Philippines | Low equipment requirements may facilitate scalability if implemented in routine practice; community and government engagement may contribute to long-term sustainability. | Initial dual-tasking challenges and embarrassment in group sessions may affect adherence. | Collaboration with community leaders and health professionals for recruitment and retention. | DTZ reported potential benefits for cognition and quality of life in older adults with MCI in a low-resource setting. | Larger, long-term studies are needed to assess cost-effectiveness and evaluate the need for booster sessions. |
| Park et al., 2011, South Korea | Multidisciplinary and senior center format may support scalability; high retention observed in the trial may inform sustainability in routine practice. | Maintaining participant engagement beyond short program duration; cultural adaptation may be needed for implementation in different settings. | Delivery through senior centers; engagement by participants and staff. | Integrated health education and exercise program reported potential benefits for hypertension management in older adults. | Research should focus on long-term studies to assess sustained effects and adapt the intervention for different cultural contexts. |
| Piedra et al., 2018, United States | Low-cost community setting may support scalability; high retention observed in the trial may inform sustainability in routine practice. | Maintaining long-term participant engagement; long-term sustainability needs further study. | Delivery in community-based senior centers with local organizational involvement. | Attribution retraining reported potential benefits for physical activity outcomes in older adults, particularly for short- to mid-term follow-up. | Long-term studies should assess effectiveness across various community settings and explore integration into broader public health initiatives. |
| Piette et al., 2023, United States | Platform-based format may facilitate scalability if implemented in routine practice; volunteer engagement may contribute to long-term sustainability. | Maintaining participant engagement over long periods; technology access barriers. | Volunteers recruited from community sources; direct engagement with English language learners (ELLs). | SPEAK! reported potential benefits for cognitive engagement in older adults at risk for cognitive decline. | Larger, long-term trials should confirm cognitive and psychological benefits and explore additional outcomes including mental health and quality of life. |
| Quach et al., 2022, Canada | Large sample and multicenter format may support scalability; long-term follow-up may inform sustainability in routine practice. | Maintaining participant engagement over 2 years. | Community-based recruitment and program delivery. | Physical activity intervention reported potential benefits for reducing major mobility disability and death, particularly in frailer older adults. | Research should investigate more intensive or multi-component physical activity programs, especially for less frail populations. |
| Reed et al., 2018, Australia | Program structure may support scalability; integration with routine care may contribute to long-term sustainability. | High clinician training requirements; limited resources for scaling; integration with existing systems needed. | Delivery involving program participants and clinicians. | CDSMS reported potential benefits for self-rated health in older adults with multiple chronic conditions; further refinement may be needed for broader health outcomes. | Future studies should explore long-term effects and integration with routine care, including enhanced care coordination. |
| Reid et al., 2019, United States | Senior center infrastructure may support scalability; community integration may contribute to long-term sustainability. | Low adherence to optional physical activity sessions; recruitment barriers identified. | Partnership with local Council on Aging; community-based program delivery. | ENGAGE reported potential for translating the LIFE physical activity intervention to community settings, with possible benefits for physical and cognitive outcomes. | Research should explore training community-based personnel, improve recruitment strategies, and assess long-term outcomes in diverse real-world settings. |
| Rejeski et al., 2017, United States | YMCA-based delivery may support scalability; partnership with established organizations may contribute to long-term sustainability. | Maintaining long-term adherence and weight management over 18 months. | Delivery in partnership with YMCA staff. | Community-based weight loss programs combined with aerobic or resistance training reported potential benefits for mobility and muscle strength in older adults. | Studies should explore methods to enhance long-term adherence and investigate protein intake in preserving muscle strength. |
| Rubenstein et al., 1994, United States | Nurse practitioner–led format may support cost-effective scalability; health system integration may contribute to long-term sustainability. | Participant adherence and coordination with primary care physicians. | Regular home visits and personalized care by nursing staff. | In-home CGA reported potential benefits for health outcomes in older adults; findings were described in the source publication without formal statistical reporting. | Continued monitoring beyond the initial study period is needed to assess long-term outcomes and broader implementation potential. |
| Shake et al., 2018, United States | App-based format may facilitate scalability if implemented in routine practice; senior center infrastructure may contribute to long-term sustainability. | Maintaining long-term engagement; ensuring technological accessibility. | Staff trained to support app use; researcher checks to ensure proper delivery. | Bingocize reported potential benefits for physical and cognitive health in older adults. | Further studies with diverse populations are needed to compare game-based interventions with traditional approaches and assess long-term efficacy. |
| Sheffield et al., 2012, United States | Restorative approach may support cost-effective scalability; broad implementation may contribute to long-term sustainability. | Ensuring consistent therapist delivery; need for individual program customization. | Delivery and assessment by occupational therapists in public agency settings. | Restorative occupational therapy intervention reported potential benefits for older adults in public agency settings. | National dissemination should be explored, with research focused on training consistency, effectiveness across settings, and long-term outcomes. |
| Shumway-Cook et al., 2007, United States | Use of existing public health resources may support scalability; public-private partnerships may contribute to long-term sustainability. | Low adherence to exercise classes; health-related dropout. | Implemented through public-private and state-local partnerships. | Multifactorial intervention reported potential benefits for fall risk factors including balance and strength, particularly in higher-risk older adults. | Research should focus on strategies to increase exercise adherence, target higher-risk groups, and involve healthcare providers more actively. |
| Shvedko et al., 2020, United Kingdom | Existing recruitment infrastructure may support scalability; adherence challenges may limit potential sustainability without additional support. | Recruitment difficulties; low attendance; transportation and weather barriers. | Participant engagement during sessions; feedback gathered through focus groups. | Group-based physical activity intervention reported potential benefits for loneliness and psychosocial outcomes; feasibility challenges were identified. | Future studies should address recruitment and transportation barriers, explore walk-level stratification, and assess long-term effects. |
| Smail et al., 2023, United States | Large sample may support scalability; high adherence observed in the trial may inform sustainability in routine practice. | Motivation among participants with depressive symptoms; sustaining engagement. | Structured sessions and home-based exercises; high adherence in both groups. | Physical activity intervention reported potential benefits for reducing mobility disability regardless of depressive symptom status. | Future research should explore whether physical activity can alleviate depressive symptoms over time and assess structured versus unstructured activity on motivation. |
| Smith-Ray et al., 2014, United States | Community center delivery may support scalability; long-term sustainability may require adequate retention and adherence. | Participant retention challenges due to personal and health-related issues. | Delivery in senior and community centers. | Cognitive training intervention reported potential benefits for balance and gait speed in older adults. | Research should investigate long-term maintenance of effects, optimal dosing, and mechanisms linking cognition to mobility. |
| Song & Yu, 2019, China | Community center group format may support scalability; long-term adherence may contribute to long-term sustainability. | Maintaining long-term engagement and adherence beyond the study period. | Delivery in community healthcare centers; motivational strategies used. | Moderate-intensity aerobic exercise reported potential benefits for cognitive function and quality of life in older adults with MCI. | Future studies should assess long-term effects and applicability in other settings, with a focus on psychological and sleep hygiene components. |
| Song et al., 2024, China and Hong Kong | Group format and community health center delivery may support scalability; high adherence observed in the trial may inform sustainability in routine practice. | Participant retention over longer program duration. | Delivery in community health centers; group-based participation. | Aerobic dancing program reported potential benefits for sleep quality and cognitive function in older adults with MCI. | Research should explore mobile health applications to facilitate exercise, extend program duration, and incorporate objective sleep measures. |
| Spoorenberg et al., 2018, Netherlands | Care team training may support scalability; ongoing professional development may contribute to long-term sustainability. | Required cultural change in professional behavior; a high-standard healthcare system may reduce observable benefits. | Community meetings; participant involvement in care planning. | Embrace did not demonstrate clear benefits for patient-reported outcomes in this trial; further refinement is needed before broader implementation. | Longer-term evaluations with sensitive outcome measures are needed to assess applicability across diverse geographical and cultural contexts. |
| Stuck et al., 1995, United States | Healthcare service integration may support scalability and cost-effectiveness; long-term sustainability may require adequate training and funding. | High initial setup costs; need for consistent follow-up. | Participants encouraged to engage with community services; direct community involvement in design and delivery was limited. | In-home comprehensive geriatric assessment reported potential for delaying disability and reducing nursing home admissions; costs and training requirements should be considered in planning. | Research should identify effective intervention components and adapt the approach for different populations and settings. |
| Stuck et al., 2000, Switzerland | Trained public health nurses and geriatricians may support scalability; long-term cost savings may contribute to long-term sustainability. | Variability in nurse performance; careful targeting and monitoring of high-risk older adults; trained professional requirements. | In-home visits facilitated by nurses; engagement with healthcare providers. | Preventive home visits with multidimensional assessment reported potential benefits in low-risk older adults; caution is warranted for high-risk individuals. | Future research should refine beneficiary selection criteria, improve nurse training, and investigate the impact of nurse performance on outcomes. |
| Sugiyama et al., 2015, United States | Non-professional health educator format and cultural adaptation may support scalability; group-based delivery may contribute to long-term sustainability. | Cultural relevance; consistent health educator training; sustaining participant engagement. | Group sessions with culturally relevant content; non-professional health educators. | DSME program reported potential benefits for mental health-related quality of life (HRQoL) in older adults with diabetes. | Research should clarify mechanisms through which DSME improves HRQoL and explore long-term effects in diverse populations. |
| Szanton et al., 2011, United States | Low-cost home modification approach may support scalability; community-based delivery may contribute to long-term sustainability. | Availability of trained professionals and funding for home modifications. | Intervention tailored to individual needs with attention to cultural relevance; delivery through community-based occupational and nursing staff. | CAPABLE reported potential benefits for reducing disability and improving quality of life in low-income older adults. | Larger trials are needed to confirm effectiveness, and future studies should explore long-term impact on nursing home admissions and healthcare transitions. |
| Szanton et al., 2014, United States | Integration into healthcare models may support scalability; cost-effectiveness findings may contribute to long-term sustainability and policy interest. | Scaling may require additional healthcare provider training and securing consistent funding. | Strong partnerships with community organizations and local government agencies. | CAPABLE reported potential cost-effectiveness and may inform the development of future community-based models for reducing disability and improving quality of life in low-income older adults. | Future research should explore long-term sustainability and assess the impact of booster visits on maintaining functional independence. |
| Taylor et al., 2016, United Kingdom | Low-cost format may support scalability; long-term disability outcomes require further evidence. | Maintaining participant engagement; need for longer-term program support. | Community-based delivery; high participant involvement in program activities. | COPERS reported potential benefits for psychological well-being in chronic pain patients with depression; primary disability outcomes were not significantly improved. | Research should assess combining COPERS with exercise programs to improve pain-related disability outcomes, particularly in patients with depression. |
| Uemura et al., 2018, Japan | Low-cost community format may support scalability; community engagement may contribute to long-term sustainability. | Maintaining participant motivation and adherence over time. | Group work and local support center involvement. | Active learning program reported potential benefits for health literacy and behaviors in older adults. | Future studies should include larger samples, longer follow-up, and adaptations for individuals with chronic diseases. |
| Vaz Fragoso et al., 2015, United States | High adherence observed in the trial may support scalability; ongoing monitoring may inform sustainability in routine practice. | Long-term adherence to physical activity; potential for dropout. | Regular attendance at community-based sessions. | Structured physical activity program reported potential benefits for reducing poor sleep quality in sedentary older adults. | Research should explore long-term adherence and the impact on sleep quality and other health outcomes. |
| Wang et al., 2016, China | Digital database management and community education format may support scalability; workshop-based delivery may contribute to long-term sustainability. | Maintaining participant adherence; managing long-term engagement. | Regular community education sessions and communication activities. | Osteoporosis club model reported potential benefits for osteoporosis prevention in this trial. | Further studies should assess long-term effects on fragility fractures with larger samples and additional evaluation indices such as muscle function. |
| Wolf et al., 1996, United States | Low-cost Tai Chi format may support scalability; informal continuation post-study may contribute to long-term sustainability. | Adherence, particularly for balance training which requires specific equipment. | Tai Chi participants continued meeting informally after the trial, indicating strong community engagement. | Tai Chi reported potential benefits for fall reduction and physical and psychosocial well-being in older adults. | Long-term studies should assess impacts on falls and well-being and explore effectiveness across diverse populations with varied intervention intensity. |
| Wong et al., 2020, Hong Kong | Collaboration with community health services may support scalability; professional engagement may contribute to long-term sustainability. | Human resource-intensive; difficulties in maintaining multi-agency collaboration. | Involvement of community health and social services in intervention delivery. | Health-social partnership model reported potential benefits for HRQoL and depressive symptoms in non-frail older adults. | Large-scale studies are needed to test sustainability and cost-effectiveness, and to explore e-health applications to reduce human resource requirements. |
| Wong et al., 2022, Hong Kong | Health-social partnership model may support scalability; professional engagement may contribute to long-term sustainability. | Resource-intensive; challenges in maintaining regular interactions and coordination. | Involvement of community health centers and social services. | mHealth app combined with nurse case management reported potential for enhancing health outcomes; quality of life improvements were not statistically significant in this trial. | Future studies should explore flexible telecare, AI decision support integration, and long-term cost-effectiveness of mHealth+I programs. |
| Woo et al., 2024, Taiwan | Minimal equipment requirements may facilitate scalability if implemented in routine practice; trained instructor involvement may contribute to long-term sustainability. | Adherence may decrease without consistent community support or resources. | Delivery through a Veterans care institution; group-based session format. | Resistance band exercise combined with Tai Chi reported potential benefits for physical fitness and quality of life in prefrail older adults. | Future studies should investigate longer-term interventions (16–24 weeks) and explore effectiveness across diverse populations and settings. |
| Wu et al., 2019, Taiwan | Integration with community health screening may support scalability; ongoing professional support may contribute to long-term sustainability. | Maintaining long-term engagement and follow-up. | Participant involvement in regular community health screenings. | Nurse-led health promotion program reported potential benefits for health outcomes in adults at cardiometabolic risk. | Research should explore digital approaches to enhance adherence, with longer follow-up in larger, more diverse populations. |
| Xu et al., 2020, China | Rural healthcare institution infrastructure may support scalability; team training may contribute to long-term sustainability. | Maintaining consistent participation and follow-up, particularly in adverse weather conditions. | Involvement of county, township, and village-level healthcare institutions. | Integrated healthcare program reported potential benefits for HRQoL and physical health outcomes in older adults with hypertension in rural settings. | Future studies should explore long-term impacts and strategies for sustaining patient engagement post-intervention. |
| Yang et al., 2023, China | Speech pathologist-led format may support scalability; volunteer training may contribute to long-term sustainability. | Maintaining engagement; training sufficient volunteers; ensuring long-term adherence. | Active involvement of SLPs, family members, and caregivers in community settings. | Community-based rehabilitation program reported potential benefits for swallowing function, mood, and quality of life in stroke patients with dysphagia. | Research should assess long-term benefits and feasibility of scaling across communities, with focus on sustainability and diverse clinical outcomes. |
| Zhao et al., 2023, China | Nurse-led format may support scalability; high adherence observed in the trial may inform sustainability in routine practice. | Sustaining caregiver and participant engagement; need for further research on long-term effects. | Delivery by community healthcare providers; engagement of community-dwelling older adults. | P-bM-tACT reported potential benefits for cognitive function in older adults with MCI in community settings. | Long-term studies are needed to confirm sustainability of cognitive benefits and explore transfer effects to other populations. |

Note: Scalability and sustainability assessments were derived from intervention characteristics reported in the source publications and interpreted by the review authors; these factors were not always directly evaluated within the trials themselves. Community involvement refers to participation in recruitment processes, delivery settings, program facilitation, or community partnerships as reported in the original studies; levels and types of engagement varied considerably across studies and should not be assumed to reflect formal co-design or community governance unless explicitly stated. Practice implications are framed cautiously to reflect individual trial findings and should not be interpreted as guideline-level recommendations. Detailed study-level risk of bias assessments are reported in Supplementary Table 5. Some randomized controlled trials were reported in multiple publications; therefore, the number of publications (n = 91) exceeds the number of unique trials (n = 85). Brown et al., 2020 represents a secondary analysis of the LIFE trial cohort and was retained because it reported additional outcomes relevant to functional independence and implementation considerations, not because it is a standalone primary RCT intervention report.

Abbreviations: ADL = activities of daily living; AI = artificial intelligence; AMP = Aging Mastery Program; AST = activity-based strategy training; BAILAMOS = Bailamos Dance Program; CAPABLE = Community Aging in Place — Advancing Better Living for Elders; CBT = cognitive behavioral therapy; CDSMS = chronic disease self-management support; CERgAS = Community Exercise and Rehabilitation for older adults for Getting Active and Strong; CHW = community health worker; CN = congregate nutrition; COPERS = Coping with Persistent Pain, Effectiveness Research in Self-management; CCTS = Composite Cognitive Test Score; DSME = diabetes self-management education; DTZ = dual-task Zumba; ELL = English language learner; GAR = group audiological rehabilitation; HAHA II = Home-based Active Healthy Aging II; HRQoL = health-related quality of life; IADL = instrumental activities of daily living; LoChro-Care = Longitudinal Chronic Care; MCI = mild cognitive impairment; mHealth = mobile health; MMD = major mobility disability; OA = osteoarthritis; PA = physical activity; PAPP = Physical Activity Promotion Programme; PCDS = pre-clinical disability status; PLE2NO = Physical activity and Education to prevent Knee Osteoarthritis; P-bM-tACT = practice-based Mindfulness Training for Alzheimer's Caregiver Transformation; QoL = quality of life; RCT = randomized controlled trial; SLP = speech-language pathologist; SPEAK! = Sharing Personal Experiences and Knowledge; SPPB = Short Physical Performance Battery; TTM = Transtheoretical Model; VON = Victorian Order of Nurses; WL = weight loss; YMCA = Young Men's Christian Association.
