# Supplementary material for: Community-based interventions to support aging in place and functional independence in older adults: a systematic review of randomized controlled trials
Source: Front Public Health. 2026 May 15;14:1828271. doi: 10.3389/fpubh.2026.1828271 (PMC13219341; doi:10.3389/fpubh.2026.1828271)
Supplement: Supplementary file 5 [file Table_5.DOCX]

**Supplementary Table 5. Study-Level Risk of Bias Assessment Across Thirteen Domains According to the Joanna Briggs Institute (JBI) Critical Appraisal Checklist for Randomized Controlled Trials**

| **Study** | **D1** | **D2** | **D3** | **D4** | **D5** | **D6** | **D7** | **D8** | **D9** | **D10** | **D11** | **D12** | **D13** | **Overall Risk of Bias** |
| --- | --- | --- | --- | --- | --- | --- | --- | --- | --- | --- | --- | --- | --- | --- |
| Acton 2016 | Low | Low | Low | High | High | Low | Low | Low | Low | Low | Low | Low | Low | **Low** |
| Arai 2007 | Unclear | Unclear | Low | Unclear | Unclear | Low | Low | Low | Low | Low | Unclear | Low | Low | **Moderate** |
| Bae 2019 | Low | Low | Low | High | High | Low | Low | Low | Low | Low | Low | Low | Low | **Low** |
| Bann 2016 | Low | Unclear | Low | High | High | Low | Unclear | Low | Low | Low | Low | Low | Low | **Moderate** |
| Brown 2020 | Low | Unclear | Low | High | High | Low | Low | Low | Low | Low | Low | Low | Low | **Moderate** |
| Chao 2012 | Low | Low | Low | High | High | Low | Unclear | Low | Low | Low | Low | Low | Low | **Moderate** |
| Chen 2021 | Low | Low | Low | High | High | Low | Low | Low | Low | Low | Low | Low | Low | **Low** |
| Clark 1997 | Low | Low | Low | High | High | Low | Low | Low | Low | Low | Low | Low | Low | **Low** |
| Clark 2002 | Low | Unclear | Low | High | High | Low | Unclear | Low | Low | Unclear | Unclear | Low | Low | **Moderate** |
| Cwirlej-Sozanska et al., 2018 | Low | Low | Low | High | High | Low | Low | Low | Low | Low | Low | Low | Low | **Low** |
| Ekelund & Eklund 2015 | Low | Low | Low | High | High | Low | High | Low | Low | Low | Low | Low | Low | **High** |
| Eklund et al., 2008 | Low | Unclear | Low | High | High | Low | High | Low | Low | Low | Low | Low | Low | **High** |
| Endevelt et al., 2011 | Unclear | Unclear | Low | High | High | Low | High | Low | Low | Low | Low | Low | Low | **High** |
| Estebsari et al., 2018 | Low | Unclear | Low | High | High | Low | High | Low | Low | Low | Low | Low | Low | **High** |
| Evans et al., 2021 | Low | Low | Low | High | High | Low | Low | Low | Low | Low | Low | Low | Low | **Low** |
| Feng et al., 2020 | Low | Low | Low | High | High | Low | Low | Low | Low | Low | Low | Low | Low | **Low** |
| Fielding et al., 2017 | Low | Low | Low | High | High | Low | Low | Low | Low | Low | Low | Low | Low | **Low** |
| Giné-Garriga et al., 2013 | Low | Unclear | Low | High | High | Low | Low | Low | Low | Low | Low | Low | Low | **Moderate** |
| Gitlin et al., 2006 | Low | Low | Low | High | High | Low | Low | Low | Low | Low | Low | Low | Low | **Low** |
| González-Guerrero et al., 2014 | Low | Unclear | Low | High | High | Low | Unclear | Low | Low | Low | Low | Low | Low | **Moderate** |
| Groessl et al., 2016 | Low | Unclear | Low | High | High | Low | Low | Low | Low | Low | Low | Low | Low | **Moderate** |
| Guerrero 2020 | High | High | Low | High | High | Unclear | Unclear | Low | Low | High | High | Low | Unclear | **High** |
| Hernandez 2019 | Low | Unclear | Low | High | High | Low | Unclear | Low | Low | Low | Low | Low | Low | **Moderate** |
| Janevic 2022 | Low | Low | Low | High | High | Low | High | Low | Low | Low | Low | Low | Low | **High** |
| Johnson 2018 | Low | Low | Low | High | High | Low | Low | Low | Low | Low | Low | Low | Low | **Low** |
| Jones 2019 | Low | Unclear | Low | High | High | Low | Unclear | Low | Low | Low | Low | Low | Low | **Moderate** |
| Keall 2017 | Low | Low | Low | High | High | Low | Low | Low | Low | Low | Low | Low | Low | **Low** |
| Khodneva 2021 | Low | Unclear | Low | High | High | Low | Unclear | Low | Low | Low | Low | Low | Low | **Moderate** |
| Kim 2013 | Low | Unclear | Low | High | High | Low | High | Low | Low | Low | Low | Low | Low | **High** |
| Kim 2016 | Low | Unclear | Low | High | High | Low | Unclear | Low | Low | Low | Low | Low | Low | **Moderate** |
| King 2017 | Low | Unclear | Low | High | High | Low | Unclear | Low | Low | Low | Low | Low | Low | **Moderate** |
| King et al. 2021 | Low | Unclear | Low | High | High | Low | Unclear | Low | Low | Unclear | Low | Low | Low | **Moderate** |
| King et al., 2007 | Low | Unclear | Low | High | High | Low | Low | Low | Low | Low | Low | Low | Low | **Moderate** |
| Kohn et al. 2023 | Low | Low | Low | High | High | Low | Low | Low | Low | Low | Low | Low | Low | **Low** |
| Lamb et al. 2020 | Low | Low | Low | High | High | Low | Low | Low | Low | Low | Low | Low | Low | **Low** |
| Lee et al. 2023 | Low | Low | Low | High | High | Low | Low | Low | Low | Low | Low | Low | Low | **Low** |
| Liang et al. 2021 | Low | Unclear | High | High | High | Low | Unclear | Low | Low | Low | Low | Low | Low | **High** |
| Liao et al. 2018 | Low | High | Low | High | High | Low | Low | Low | Low | Low | Low | Low | Low | **High** |
| LIFE Study Investigators 2006 | Low | Low | Low | High | High | Low | Low | Low | Low | Low | Low | Low | Low | **Low** |
| Loh et al. 2015 | Low | Low | Low | High | High | Low | Low | Low | Low | Low | Low | Low | Low | **Low** |
| Lu et al. 2015 | Low | Low | Low | High | High | Low | Low | Low | Low | Low | Low | Low | Low | **Low** |
| Marconcin et al. 2022 | Low | Low | High | High | High | Low | Low | Low | Low | Low | Low | Low | Low | **High** |
| Markle-Reid et al. 2006 | Low | Low | Low | High | High | Low | Low | Low | Low | Low | Low | Low | Low | **Low** |
| Marquez et al. 2014 | Low | Low | Low | High | High | Low | Low | Low | Low | Low | Low | Low | Low | **Low** |
| Marquez et al. 2017 | Low | Low | Low | High | High | Low | Unclear | Low | Low | Low | Low | Low | Low | **Moderate** |
| Martín-Valero et al. 2013 | Unclear | High | Low | High | High | Low | Low | Low | Low | High | Low | Low | Low | **High** |
| Meng et al. 2024 | Low | Low | Low | High | High | Low | Low | Low | Low | Low | Low | Low | Low | **Low** |
| Metzner et al. 2023 | Low | Low | Low | High | High | Low | Unclear | Low | Low | Low | Low | Low | Low | **Moderate** |
| Mitchell et al. 2006 | Low | Low | Low | High | High | Low | Unclear | Low | Low | High | Low | Low | Low | **High** |
| Moore-Harrison et al., 2008 | High | High | Low | High | High | Low | High | Low | Low | High | Low | Low | Low | **High** |
| Morone et al. 2016 | Low | Low | Low | High | High | Low | Low | Low | Low | Low | Low | Low | Low | **Low** |
| Murphy et al. 2008 | Low | High | Low | High | High | Low | Low | Low | Low | Unclear | Low | Low | Low | **High** |
| Ng et al. 2017 | Low | Low | Low | High | High | Low | Low | Low | Low | Low | Low | Low | Low | **Low** |
| Nikolaus & Bach 2003 | Low | Low | Low | High | High | Low | Low | Low | Low | Low | Low | Low | Low | **Low** |
| Oh et al. 2017 | Low | High | Low | High | High | Low | Unclear | Low | Low | High | Unclear | Low | Low | **High** |
| Oh et al. 2021 | Low | Unclear | Low | High | High | Low | Unclear | Low | Low | High | Low | Low | Low | **High** |
| Parial et al., 2023 | Low | Low | Low | High | High | Low | Low | Low | Low | Low | Low | Low | Low | **Low** |
| Park et al. 2011 | Low | Unclear | Low | High | High | Low | Unclear | Low | Low | Low | Low | Low | Low | **Moderate** |
| Piedra et al. 2018 | Low | Low | Low | High | High | Low | Low | Low | Low | Low | Low | Low | Low | **Low** |
| Piette et al. 2023 | Low | Low | Low | High | High | Low | Unclear | Low | Low | Low | Low | Low | Low | **Moderate** |
| Quach et al. 2022 | Low | Low | Low | High | High | Low | Low | Low | Low | Low | Low | Low | Low | **Low** |
| Reed et al. 2018 | Low | Low | Low | High | High | Low | Unclear | Low | Low | Low | Low | Low | Low | **Moderate** |
| Reid et al. 2019 | Low | Low | Low | High | High | Low | Low | Low | Low | Unclear | Low | Low | Low | **Moderate** |
| Rejeski et al. 2017 | Low | Low | Low | High | High | Low | Low | Low | Low | Low | Low | Low | Low | **Low** |
| Rubenstein et al. 1994 | Low | Low | Low | High | High | Low | High | Low | Low | Low | Low | Low | Low | **High** |
| Shake et al. 2018 | High | High | Low | High | High | Low | Unclear | Low | Low | Low | Low | Low | Low | **High** |
| Sheffield et al. 2012 | Low | Unclear | Low | High | High | Low | High | Low | Low | Low | Low | Low | Low | **High** |
| Shumway-Cook et al. 2007 | Low | Low | Low | High | High | Low | Low | Low | Low | Low | Low | Low | Low | **Low** |
| Shvedko et al. 2020 | Low | Low | Low | High | High | Low | High | Low | Low | Low | Low | Low | Low | **High** |
| Smail et al. 2023 | Low | Low | Low | High | High | Low | Unclear | Low | Low | Low | Low | Low | Low | **Moderate** |
| Smith-Ray et al. 2014 | Low | High | Low | High | High | Low | Unclear | Low | Low | Low | Low | Low | Low | **High** |
| Song & Yu 2019 | Low | Low | Low | High | High | Low | Low | Low | Low | Low | Low | Low | Low | **Low** |
| Song et al. 2024 | Low | Low | Low | High | High | Low | Low | Low | Low | Low | Low | Low | Low | **Low** |
| Spoorenberg et al. 2018 | Low | Low | Low | High | High | Low | Low | Low | Low | Low | Low | Low | Low | **Low** |
| Stuck et al. 1995 | Low | Low | Low | High | High | Low | Low | Low | Low | Low | Low | Low | Low | **Low** |
| Stuck et al. 2000 | Low | Low | Low | High | High | Low | Low | Low | Low | Low | Low | Low | Low | **Low** |
| Sugiyama et al. 2015 | Low | Low | Low | High | High | Low | Unclear | Low | Low | Low | Low | Low | Low | **Moderate** |
| Szanton et al. 2011 | Low | Low | Low | High | High | Low | Low | Low | Low | Low | Low | Low | Low | **Low** |
| Szanton et al. 2014 | Low | Low | Low | High | High | Low | Low | Low | Low | Low | Low | Low | Low | **Low** |
| Taylor et al. 2016 | Low | Low | Low | High | High | Low | Unclear | Low | Low | Low | Low | Low | Low | **Moderate** |
| Uemura et al. 2018 | Low | Low | Low | High | High | Low | Low | Low | Low | Low | Low | Low | Low | **Low** |
| Vaz Fragoso et al. 2015 | Low | Low | Low | High | High | Low | Low | Low | Low | Low | Low | Low | Low | **Low** |
| Wang et al. 2016 | Low | Unclear | Low | High | High | Low | Unclear | Low | Low | Low | Low | Low | Low | **Moderate** |
| Wolf et al. 2003 | Low | Low | Low | High | High | Low | Low | Low | Low | Unclear | Low | Low | Low | **Moderate** |
| Wong et al. 2020 | Low | Low | Low | High | High | Low | Low | Low | Low | Low | Low | Low | Low | **Low** |
| Wong et al. 2022 | Low | Low | Low | High | High | Low | Low | Low | Low | Low | Low | Low | Low | **Low** |
| Woo et al. 2024 | Low | Unclear | Low | High | High | Low | Unclear | Low | Low | Low | Low | Low | Low | **Moderate** |
| Wu et al. 2019 | Low | Low | Low | High | High | Low | Unclear | Low | Low | Low | Low | Low | Low | **Moderate** |
| Xu et al. 2020 | Low | High | Low | High | High | Low | Unclear | Low | Low | Low | Low | Low | Low | **High** |
| Yang et al. 2023 | Low | Low | Low | High | High | Low | Low | Low | Low | Low | Low | Low | Low | **Low** |
| Zhao et al. 2023 | Low | Low | Low | High | High | Low | Low | Low | Low | Low | Low | Low | Low | **Low** |

Note. D1 = random sequence generation; D2 = allocation concealment; D3 = baseline similarity; D4 = participant blinding; D5 = personnel blinding; D6 = treatment groups treated identically other than the intervention of interest; D7 = outcome assessor blinding; D8 = outcomes measured in a reliable way; D9 = outcomes measured in the same way for treatment groups; D10 = completeness of follow-up; D11 = intention-to-treat analysis; D12 = appropriateness of statistical analysis; D13 = appropriateness of trial design. Low = low risk of bias; Unclear = unclear risk of bias; High = high risk of bias. Overall risk of bias was determined based on domains D1–D3 and D6–D13. Domains D4 and D5 were not weighted in the overall study-level classification because blinding of participants and personnel is typically not feasible in behavioral and community-based interventions.
