# Supplementary material for: Community-based interventions to support aging in place and functional independence in older adults: a systematic review of randomized controlled trials
Source: Front Public Health. 2026 May 15;14:1828271. doi: 10.3389/fpubh.2026.1828271 (PMC13219341; doi:10.3389/fpubh.2026.1828271)
Supplement: Supplementary file 6 [file Table_6.DOCX]

**Supplementary Table S6. Full electronic search strategy for MEDLINE (PubMed)**

**Database:** MEDLINE via PubMed
**Search date:** October 2024
**Coverage:** Database inception to 30 September 2024
**Limits:** Humans; English; Randomized Controlled Trial

| **Step** | **Search string** |
| --- | --- |
| **1 Population terms** | ("Aged"[MeSH Terms] OR elderly[tiab] OR aged[tiab] OR "older adult*"[tiab] OR "older people"[tiab] OR elder*[tiab] OR geriatric*[tiab] OR "elderly people"[tiab] OR "old people"[tiab] OR seniors[tiab] OR "aging population"[tiab]) |
| **2 Intervention terms** | ("Community Health Services"[MeSH Terms] OR "Health Promotion"[MeSH Terms] OR "public health initiative*"[tiab] OR "social care initiative*"[tiab] OR "community-based"[tiab] OR "community based"[tiab] OR "community-based intervention*"[tiab] OR "health program*"[tiab] OR "aging in place"[tiab] OR "home modification*"[tiab] OR "social support network*"[tiab] OR "health education"[tiab] OR "preventive care program*"[tiab]) |
| **3 Outcome terms** | ("Activities of Daily Living"[MeSH Terms] OR "Independent Living"[MeSH Terms] OR independence[tiab] OR autonomy[tiab] OR "functional independence"[tiab] OR "quality of life"[tiab] OR wellbeing[tiab] OR "well-being"[tiab] OR "well being"[tiab] OR mobility[tiab] OR disability[tiab] OR "cognitive function"[tiab] OR loneliness[tiab] OR "social isolation"[tiab] OR "health outcome*"[tiab]) |
| **4 Study design filter** | Randomized Controlled Trial[pt] |
| **5 Final search** | #1 AND #2 AND #3 AND Randomized Controlled Trial[pt] |

**Abbreviations**

tiab = Title/Abstract
MeSH = Medical Subject Headings
pt = Publication type

**Note:** The same conceptual search structure (population, intervention, and outcome blocks) was applied across Embase, Web of Science, CINAHL, and PsycINFO, using database-specific controlled vocabulary and indexing terms where applicable. In databases without a validated randomized controlled trial filter, study design eligibility was verified during screening.
